# Inferring cancer type-specific patterns of metastatic spread

**DOI:** 10.1101/2024.07.09.602790

**Published:** 2024-09-03

**Authors:** Divya Koyyalagunta, Karuna Ganesh, Quaid Morris

**Affiliations:** 1 Tri-Institutional Graduate Program in Computational Biology and Medicine, Weill Cornell Medicine, New York, NY 10065, USA; 2 Computational and Systems Biology Program, Sloan Kettering Institute, New York, NY 10065, USA.; 3 Department of Medicine, Memorial Sloan Kettering Cancer Center, New York, NY, USA; 4 Molecular Pharmacology Program, Sloan Kettering Institute, Memorial Sloan Kettering Cancer Center, New York, NY, USA

**Keywords:** migration history inference, metastasis, mixed-variable combinatorial optimzation

## Abstract

The metastatic spread of a cancer can be reconstructed from DNA sequencing of primary and metastatic tumours, but doing so requires solving a challenging combinatorial optimization problem. This problem often has multiple solutions that cannot be distinguished based on current maximum parsimony principles alone. Current algorithms use ad hoc criteria to select among these solutions, and decide, a priori, what patterns of metastatic spread are more likely, which is itself a key question posed by studies of metastasis seeking to use these tools. Here we introduce Metient, a freely available open-source tool which proposes multiple possible hypotheses of metastatic spread in a cohort of patients and rescores these hypotheses using independent data on genetic distance of metastasizing clones and organotropism. Metient is more accurate and is up to 50x faster than current state-of-the-art. Given a cohort of patients, Metient can calibrate its parsimony criteria, thereby identifying shared patterns of metastatic dissemination in the cohort. Reanalyzing metastasis in 169 patients based on 490 tumors, Metient automatically identifies cancer type-specific trends of metastatic dissemination in melanoma, high-risk neuroblastoma and non-small cell lung cancer. Metient’s reconstructions usually agree with semi-manual expert analysis, however, in many patients, Metient identifies more plausible migration histories than experts, and further finds that polyclonal seeding of metastases is more common than previously reported. By removing the need for hard constraints on what patterns of metastatic spread are most likely, Metient introduces a way to further our understanding of cancer type-specific metastatic spread.

## Introduction

Metastasis is associated with 90% of cancer deaths, yet its causes and physiology remain poorly understood^1^. It remains unclear how often multiple clones seed metastases, how often metastases are capable of seeding other metastases, and if there is a relationship between seeding clones and organ-specific metastases^2–10^. It is also not known whether metastatic potential is rare, and thus gained once in the same cancer, or common, and thus gained multiple times^11–14^. The answers to all these questions would improve the understanding and clinical management of metastasis, but doing so requires reconstructing migration histories of metastatic clones from clinical sequencing data which, until recently, was very challenging^2–4^.

Recent algorithms have tackled this challenge using maximum parsimony principles. These algorithms identify parsimonious migration histories that explain the clonal compositions of primary tumors and one or more matched metastatic tumors^5,15–17^. However, different definitions of parsimony can disagree on the best solution, and current algorithms resolves these conflicts using ad hoc rules^15–17^. For example, a common rule is to only allow metastases to be seeded from the primary^14^, whereas determining whether metastases can seed other metastases is, itself, an important question. Indeed, one prevailing model in oncology, the “sequential progression model” – which posits that lymph node metastases give rise to distant metastases – is the rationale for surgical removal of lymph nodes^18^. However, a recent phylogenetic analysis found that the sequential model only applied to a third of patients in a colorectal cohort^19^. By pre-biasing their reconstructions with ad hoc rules, current algorithms undermine a key goal in making these reconstructions: determining which patterns of metastatic spread are prevalent in different cancer types.

To address this dilemma and overcome the limitations of previous tools (Supplementary Table 1), we introduce Metient (**met**astasis + grad**ient**). Metient is a principled statistical algorithm that proposes multiple potential hypotheses of metastatic spread in a patient and resolves parsimony conflicts using other, readily-available data. Metient achieves this through two key innovations. First, it adapts recent stochastic optimization algorithms for discrete variables to the problem of combinatorial optimization, thereby enabling efficient sampling of multiple parsimonious solutions. Second, it introduces new biological criteria, termed metastasis priors, to calibrate its parsimony criteria and select among equally parsimonious solutions. These calibrated criteria can also be used to uncover cancer type-specific trends in metastatic spread.

On realistic simulated data, Metient outperforms parsimony-only models in accurately recovering the true migration history. When applied to patient cohorts with metastatic breast^20^, skin^3^, ovarian^4^, neuroblastoma^9^, and lung cancer^14^, Metient automatically identifies all plausible expert-assigned migration histories. In notable cases, it also uncovers more plausible reconstructions, often when prior expert analyses pre-selected a favored seeding pattern.

Through its unbiased automated approach, Metient reveals that metastases are often seeded polyclonally and that most metastatic seeding follows a single, shared evolutionary trajectory. The cancer type-specific models learned by Metient reflect known differences in metastasis biology, suggesting that Metient can offer insights into metastatic dissemination for new cancer cohorts.

Metient is free, open-source software that includes easy-to-use visualization tools to compare multiple hypotheses on metastatic dissemination. Metient is accessible at https://github.com/morrislab/metient/.

## Results

### The Metient algorithm

**Figure 1.**
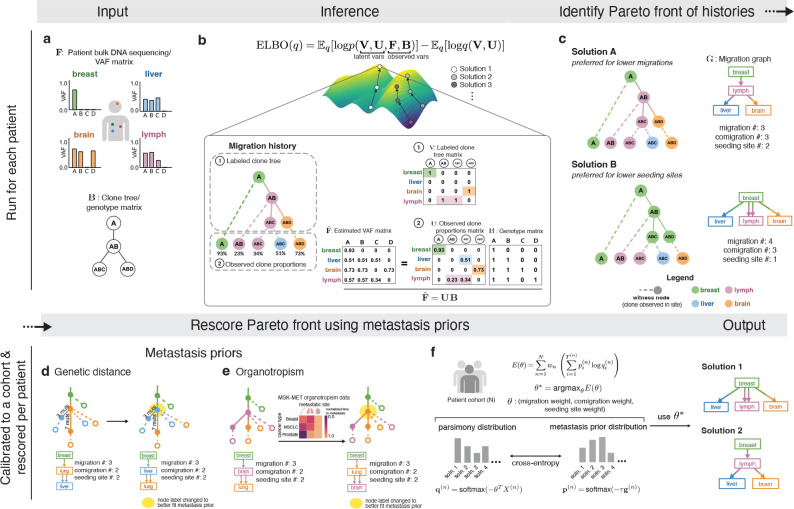
Overview of the Metient method. **(a) Input**: (top) bulk DNA sequencing sampled from multiple tumors in a single patient, and (bottom) a clone tree which represents the evolutionary relationship of mutations. AB refers to a clone with mutations or mutation clusters A and B. **(b) Inference**: Using the inputs as observed variables, we infer the latent variables (1) V (representing the labeled clone tree) and (2) U (representing the proportion of each clone in each anatomical site). $\overset{ˆ}{F}$ is the estimated VAF matrix produced by UB, where $B_{ij}=1$ if clone i contains mutation j. Each migration history solution can be represented by a migration history, which is a clone tree with (1) an anatomical site labeling of its internal nodes, and (2) leaf nodes representing the observed clone proportions in anatomical sites. **(c) Identify Pareto front of histories**: We infer a Pareto front of migration histories as defined by the three parsimony metrics (migration, comigration and seeding site number). A migration graph G summarizes the migration edges of the migration history. **(d) Genetic distance**: An example of how using genetic distance can promote migration histories with migrations on longer edges with more mutations. The anatomical site label of the yellow shaded node is changed. **(e) Organotropism**: An example of how using organotropism can promote migration histories that do not contain unlikely metastatic patterns, such as subsequent metastasis from the brain. The anatomical site label of the yellow shaded node is changed. **(f) Metient-calibrate**: Weights on the parsimony metrics (θ) are fit by minimizing the cross entropy loss between each patient’s migration histories’ probability distribution as scored by the metastasis priors (target distribution) and the probability distribution as scored by the parsimony metrics (source distribution). These weights are fit across a cohort of patients, and then used to rescore the Pareto front of migration histories produced for each patient in that cohort.

Migration history inference algorithms take DNA sequencing data from primary and metastatic tumor samples as input, along with an unlabeled clone tree that encodes the genetic ancestry of cancer clones (Figure 1a). These inputs are used to estimate the proportions of clonal populations in anatomical sites (referred to as “witness nodes” in Figure 1b). The internal nodes of the clone tree are then labeled with anatomical sites, defining the historical migrations: a clone that migrates to a new site receives a different label than its parent clone (Figure 1b) and the tree edge that connects them is deemed a “migration edge”. The final output is referred to as a “migration history”^17^ (Figure 1b).

MACHINA^17^ is the most widely used and most advanced migration history reconstruction algorithm. It scores migration histories using three parsimony metrics: **migrations**—the number of times a clone migrates to a different site^4,15–17^; **comigrations**—the number of migration events in which one or more clones travel from one site to another^17^; and **seeding sites**—the number of anatomical sites that seed another site^17^. MACHINA searches for the most parsimonious history by minimizing these three metrics.

This search involves solving a mixed-variable combinatorial optimization problem, consisting of continuous variables (the clone porportions matrix U in Figure 1b), and discrete variables (the labeled clone tree matrix V in Figure 1b). MACHINA, and other prior approaches, formulate this problem as a mixed integer linear programming (MILP) problem that they solve using commercial solvers^21^. However, using an MILP imposes strong limitations on the types of scoring functions that can be applied to migration histories, as MILPs require hard constraints and a linear objective. Moreover, MILP solvers identify only a single optimal solution, whereas there are often multiple solutions which are either equally parsimonious, or that trade-off one parsimony metric for the another (e.g., reducing the number of seeding sites by increasing the number of migration events). Returning a single solution obscures these possibilities, and the ad hoc rules used to distinguish among multiple solutions often introduce implicit bias into the reconstructions.

To address these issues, Metient takes a more systematic approach by first defining a “Pareto front”^22^ for each patient (Figure 1c). To do so, Metient searches for migration histories under a wide range of parsimony models (Supplementary Table 2). A parsimony model is represented by a set of parsimony weights – $w_{m},\;w_{c}$, and $w_{s}$ – assigned, respectively, to the number of migrations (indicated by m), comigrations (c), and seeding sites (s). A migration history’s parsimony score, p, is the model-weighted average of these three parsimony metrics, i.e., $p=w_{m}m+w_{c}c+w_{s}s$. Different parsimony models favor different histories on the Pareto front. Efficiently recovering this Pareto front required replacing the current state-of-the-art MILP with newly developed stochastic gradient descent methods that employ a low-variance gradient estimator for the discrete categorical distribution over migration histories parameterized by the parsimony model^23,24^ (V in Figure 1b; Methods, Supplementary Information). Metient’s gradient descent approach converges to a solution many times faster than the MILP, and it also helps to define the Pareto front by identifying multiple local maxima of the migration history score for each parsimony model (Methods, Supplementary Information). In addition, this approach reduces a large combinatorial search space of possible migration histories to only the most plausible explanations of metastatic spread for a given patient.

## Metient-calibrate fits cancer type-specific parsimony models

To illustrate the importance of defining a Pareto front of multiple possible patterns of metastatic spread, we defined four different cancer type-specific patient cohorts consisting of genomic sequencing of matched primary and multiple metastases: melanoma^3^, high-grade serous ovarian cancer (HGSOC)^4^, high-risk neuroblastoma (HR-NB)^9^, and non-small cell lung cancer (NSCLC)^14^. After applying quality control (Supplementary Information), we arrived at a dataset of 479 tumors (143 with multi-region sampling) in total from 167 patients (melanoma: n=7, HGSOC: n=7, HR-NB: n=27, NSCLC: n=126). Applying Metient to these patients, we discovered that 45% (75/167) had multiple Pareto-optimal migration histories, and that the complexity of the Pareto front increased with the number of metastases: 79% (27/34) of patient cases with three or more metastases had multiple Pareto-optimal histories. Often the choice among these different Pareto-optimal histories substantially impacted the interpretation of metastatic spread. For example, Figure 1c shows a patient with metastatic breast cancer with two Pareto-optimal reconstructions: one in which a lymph node metastasis gives rise to all other metastatic tumors, and another where most metastases are seeded directly from the primary tumor. Here, forcing an arbitrary choice between the two reconstructions determines whether one concludes that the lymph node acted as a staging site for metastatic spread.

MACHINA, and all previous methods^4,15,17^, resolve parsimony conflicts by minimizing migrations first, and then comigrations, thus implementing a parsimony model where $w_{m}>>w_{c}>>w_{s}$. However, no single parsimony model is appropriate for all cancer types. For example, in ovarian cancer, clusters of metastatic cells are thought to “passively” disseminate to the peritoneum or omentum through peritoneal fluid^25–27^. As such, metastatic events are more likely to be polyclonal, i.e., multiple clones seed metastases, so we might expect many more migrations than comigrations. In many solid cancers, metastatic cells make a “pit stop” at regional lymph nodes before disseminating to other distant sites^28^, and for the estimated 23.4% of patients with lymph node metastases across cancer types^29^, multiple seeding sites may be common. Different cancer type-specific patterns of metastatic spread are reflected in differences in trends in the relative numbers of migrations, comigrations, and seeding sites, and prespecifying a cancer type-independent parsimony model can prevent the recovery of these patterns. Furthermore, in our cohorts, we found that there were often multiple, equally parsimonious migration histories. MACHINA selects among these randomly, or via predefined constraints on the allowable patterns of metastatic spread.

In contrast, Metient uses metastasis priors to both define a cancer type-specific parsimony model and to rank equally parsimonious histories. These priors incorporate additional biological constraints relevant to migration histories. We provide a tool, Metient-calibrate, that fits a patient cohort-specific parsimony model using the metastasis priors (Figure 1d–f; Methods). This calibrated model is used to rank Pareto-optimal histories that differ in their metrics. Metient also provides a pan-cancer parsimony model, calibrated to all four cohorts combined, for use when an appropriate patient cohort is not available.

Metient provides two metastasis priors. One, genetic distance, can be applied to any cohort. The other, organotropism, can be used when appropriate tissue-type information are available for the sequenced tumor samples. The genetic distance prior considers the average genetic distance of migration edges in the labeled clone tree; where the genetic distance on an edge is the number of mutations gained in the child clone and not present in the parent clone. In general, we expect genetic distance to tend to be higher on migration edges than other clone tree edges for a number of reasons. First, the colonizing clones of a metastasis have undergone a clonal expansion in their metastatic site, which makes their private mutations more easily detectable by finite depth sequencing. In contrast, the vast majority of private mutations in the source tumor will not be at high enough cellular frequency to be detectable, and subclones detected in the source tumor need not have undergone a clonal expansion^30^. In addition to increased mutation detectability, colonizing cells likely have more mutations than randomly selected cells in the source population due to the strong selection pressures they faced in metastasizing, as strong selection pressures select, perhaps indirectly, for higher mutation rates in asexually reproducing populations^31–33^. Finally, metastases exhibit greater genomic instability^29,34,35^, possibly as a consequence of these selection pressures, which is associated with heightened mutation rates^36^. Indeed, metastases across many cancer types have moderately or significantly higher tumor mutation burden (TMB) than matched primaries^29,35,37^. Metient’s genetic distance prior deems more probable those migration histories with higher averaged genetic distances on migration edges (Methods, Supplementary Information). Figure 1d illustrates an example of using the genetic distance prior to select between two equally parsimonious migration histories.

The second metastassis prior, organotropism, is derived from data from 25,775 Memorial Sloan Kettering metastatic cancer patients^29^ on the preference that some cancer types have to colonize other organs^38^. We used these data to construct a matrix for 27 common cancer types, where each entry is the frequency of metastasis to a particular anatomical site that is observed in patients with that cancer type (Figure 1e). Note that there are no direct data for frequencies of migrations from one metastatic site to another metastatic site, so Metient only uses this matrix to score migrations coming from the primary site (Methods). For example, breast cancer metastasizes to lung more often than brain, so Metient’s organotropism prior favors a solution with migrations to the brain from a breast-seeded lung metastasis over one with migrations from a breast-seeded brain metastasis to the lung (Figure 1e). Indeed, brain to lung metastasis is rare^39^. As we illustrate in later sections, our metastasis priors lead to better performance on simulated benchmarks, and more plausible migration history reconstructions than using maximum-parsimony rules and cancer type-independent rules. Nonetheless, Metient reports all Pareto-optimal solutions; in this example, both solutions in Figure 1e are visualized in a simple summary report, so that these multiple hypotheses can be easily evaluated by the user.

Importantly, Metient uses its metastasis priors to complement but not replace its parsimony model. In our benchmarking analyses on simulated data, we find that using genetic distance alone to score migration histories performs poorly and can result in the inference of highly non-parsimonious migration histories (Supplementary Tables 4, 3, see also PathFinder^40^). Instead, the metastasis priors are only used once the Pareto front is defined, to calibrate parsimony models and to rank equally parsimonious solutions.

## Simulated data validates the genetic distance prior and shows that Metient is state-of-the-art

To assess Metient’s new objective and gradient-based optimization on data with a provided ground-truth, we ran benchmarking analyses along with the state-of-the-art migration history inference method (MACHINA^17^) on simulated data, originally used to validate MACHINA, for 80 patients with 5–11 tumor sites and various patterns of metastatic spread.

**Figure 2.**
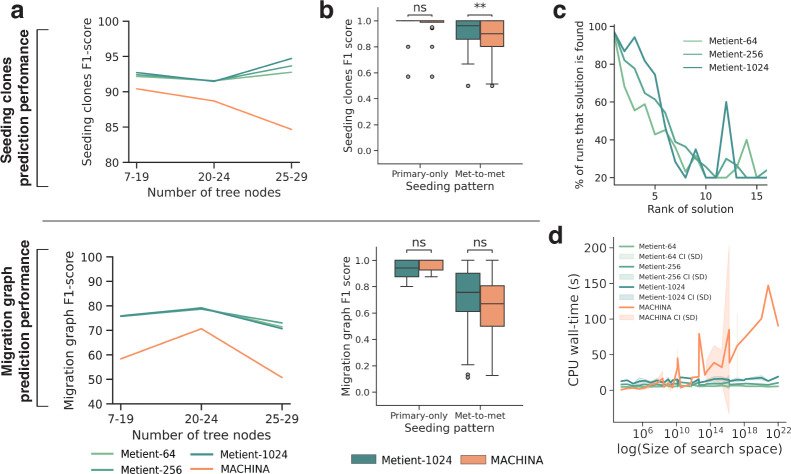
Metient achieves state-of-the-art performance on simulated data. All results shown for Metient are in calibrate mode using genetic distance as the metastasis prior. Metient-1024 refers to a model configuration where 1024 solutions are sampled. For a given simulated input, for MACHINA (which outputs one solution) the top solution is used, and for Metient we evaluate all top (lowest loss) solutions. **(a)** The averaged F1-score for predicting seeding clones (top) and migration graph (bottom), within three buckets of input tree sizes. **(b)** The distribution of F1-scores for predicting seeding clones (top) and migration graph (bottom) on different broad seeding patterns. Statistical significance assessed by a Wilcoxon signed rank test; ns: not significant, **: p=0.0021. **(c)** After running Metient five times, the percentage of runs that a certain solution is found as a function of its averaged rank across runs. **(d)** CPU wall-time needed to run Metient vs. MACHINA as a function of the search space size. CI: confidence interval, SD: standard deviation.

First, to assess the added value of the genetic distance prior, we used Metient-calibrate to fit a calibrated parsimony model, and compared calibrated Metient with a version of Metient that used the parsimony model implied by MACHINA. We fit two calibrated models, one on a cohort with primary-only seeding and another on a cohort with metastasis-to-metastasis seeding. Metient-calibrate improved recovery of the ground truth migration graph (Figure 1c) over fixed parsimony model (Calibrate vs. Evaluate (MP) in Supplementary Table 3), showcasing the ability of the metastasis priors to learn metastatic patterns specific to a cohort and improve overall accuracy. In addition, Metient-calibrate predicts ground truth seeding clones and migrations graphs at least as accurately as MACHINA, with overall improvements as tree sizes get larger (Figure 2a,b) and significant improvements in inferring the seeding clones for patients with more complex metastasis-to-metastasis seeding (Figure 2b top; p=0.0021).

Notably, although the Metient framework is non-deterministic, it identifies the same top solution 97% of the time across multiple runs (Figure 2c). Furthermore, in addition to its improved accuracy, Metient runs up to 55x faster (3.95s with Metient-64 vs. 221.19s with MACHINA for a cancer tree with 18 clones and 9 tumors), showcasing our framework’s scalability even as tree sizes get very large (Figure 2d).

### Validation of organotropism prior

**Figure 3.**
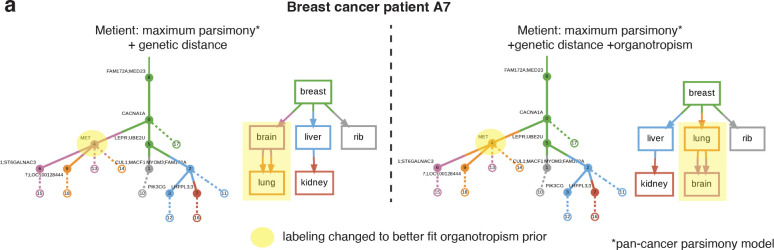
Organotropism prior corrects unlikely patterns of seeding. **(a)** The inferred migration history for breast cancer patient A7^20^ without (left) and with (right) the inclusion of the organotropism prior. The addition of an organotropism prior changes the vertex labeling of clone 4 from originating in the brain to originating in the lung. Solid edges are edges in the clone tree, and dashed edges indicate the presence of the clone in the corresponding colored anatomical site (i.e., witness nodes).

To validate the organotropism prior, we ran Metient, using the pan-cancer parsimony model, on samples available from two patients with metastatic breast cancer^20^ where site labels could be mapped to those used in our organotropism matrix. When faced with multiple parsimonious migration histories, Metient chooses a more plausible tree, wherein lung to brain seeding is preferred over brain to lung seeding, which is clinically rare^39^ (Figure 3a).

### Multi-cancer analysis of clonality, phyleticity, and dissemination patterns

Having established that Metient can accurately recover ground-truth and learn cohort-specific metastatic patterns on simulated data, we next sought to apply the method to real patient data from the melanoma, HGSOC, HR-NB and NSCLC cohorts to investigate shared and unique patterns of metastatic dissemination. Due to missing or inadequate anatomical site labels for many patients in these cohorts, we were unable to use Metient’s organotropism matrix on these cohorts, and we only calibrated to genetic distance.

**Figure 4.**
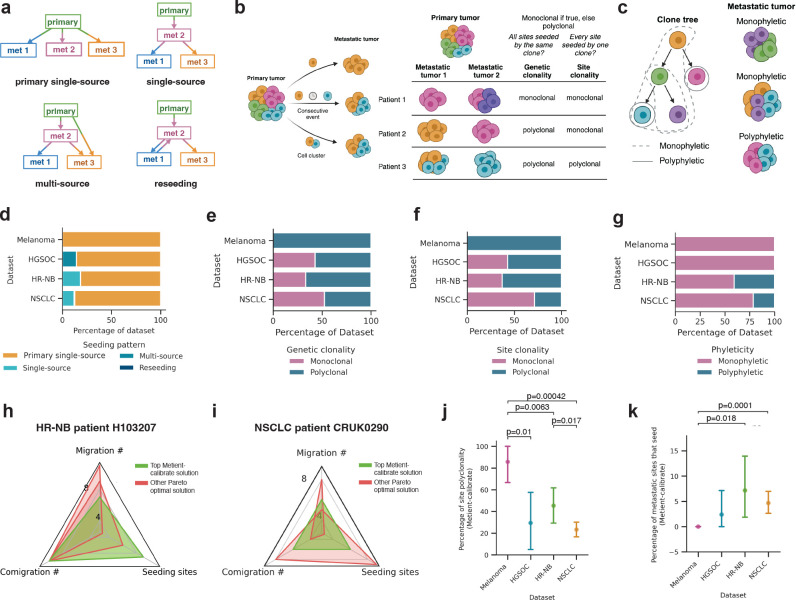
Clonal, phyletic and seeding patterns of four cancer types. **(a)** Schematic describing the four metastatic seeding patterns. met: metastasis. **(b)** Schematic depicting how metastases can get seeded by either one or multiple clones and the definitions of genetic clonality and site clonality. When a site is seeded by multiple clones, this can be a result of multiple clones traveling in a cluster to the same anatomical site, or because of two clones traveling one after the other to the same site. Colors represent genetically distinct cancer cell populations. **(c)** Schematic depicting the definitions of monophyletic and polyphletic seeding. Monophyletic indicates that the colonizing clone closest to the root can reach every other colonizing clone on the clone tree. Colors represent genetically distinct cancer cell populations. Distribution of **(d)** seeding patterns, **(e)** genetic clonality, **(f)** site clonality and **(g)** phyleticity for each dataset, as inferred by Metient’s top migration history. **(h)** Radar plot showing the unique Pareto-optimal metrics for migration histories inferred by Metient for HR-NB patient H103207. **(i)** Radar plot showing the unique Pareto-optimal metrics for migration histories inferred by Metient for NSCLC patient CRUK290. **(j)** Comparing across datasets the percent of migrations that are polyclonal for the top Metient solution. Statistical significance assessed by a Welch’s t-test. Error bars are the standard error for each dataset. **(k)** Comparing across datasets the percent of metastatic sites that seed for the top Metient solution. Statistical significance assessed by a Welch’s t-test. Error bars are the standard error for each dataset.

Using Metient, we examined three aspects of metastatic dissemination across the four cohorts. The first aspect is seeding pattern, which can be sub-categorized as single-source from the primary or from another site, multi-source, or reseeding (Figure 4a). The other two criteria are clonality, i.e., the number of distinct clones seeding metastases (Figure 4b), and phyleticity, i.e., whether metastatic potential is gained in one or multiple evolutionary trajectories of the clone tree (Figure 4c; Methods). We distinguish between genetic polyclonality, in which more than one clone seeds metastases in a patient, and site polyclonality, in which more than one clone seeds an individual site (Figure 4b; Methods). We introduce this distinction to highlight cases where each metastasis is seeded by a single clone, but all sites are not seeded by the same clone (i.e., the cancer is genetically polyclonal but site monoclonal), because these may be cases where different site-specific mutations are needed for metastasis. We also update the previous definitions of metastasis-initiating clones (commonly called seeding clones). We define a seeding or colonizing clone as a node in a migration history whose parent has a different label than itself (Methods), because this clone is the only one guaranteed to have the mutations necessary to establish the metastasis. Previous work often refers to the parent of the colonizing clone as the seeding clone^14,17^, although this clone may not have all of mutations required for the observed metastasis.

Consistent with expert annotations^3,4,9,14,17^, Metient finds that single-source seeding from the primary tumor is the most common pattern in every cohort (Figure 4d). However, Metient identifies a larger fraction of polyclonal migration patterns than previous reports^8,14^: 53.3% of patients have sites that are seeded by different clones, i.e., genetically polyclonal (Figure 4e), and 38.3% of patients have at least one site seeded by multiple clones, i.e. site polyclonal (Figure 4f). Overall, Metient estimates that 34.1% of sites (107/314) are seeded by multiple clones; nearly double prior estimates of site polyclonality (19.2%) based on an analysis of breast, colorectal and lung cancer patients^8^. Notably, parsimony model choice influences the polyclonality of migration histories, because reducing the number of seeding sites tends to increase the number of polyclonal migrations (Supplementary Figure S1a). However, the higher polyclonality in Metient’s reconstructions does not result from an assumption of primary-only seeding, as done in prior work, which would result in even more polyclonal migrations (Supplementary Figure S1a, Supplementary Information).

Metient’s phyleticity estimates mirror previous reports: 77.2% of patients (129/167) have a monophyletic tree where metastatic potential is gained once and maintained (Figure 4g). For some patients, this is due to the root clone being observed in one or more metastatic sites (Supplementary Figure S1b), and for other patients, all colonizing clones belong to a single path of the clone tree. Either scenario suggests that metastatic potential is less likely to be gained via multiple, independent evolutionary trajectories across cancers.

### Cancer type-specific metastasis trends

We next examined cancer type-specific differences in metastatic trends, first using a bootstrapping approach to ensure that the parsimony metric weights were reproducible and reflective of population level patterns for a particular cancer type. We fit parsimony metric weights to 100 bootstrapped samples of patients within the cohort (Methods), and found that 98.4% of patients ranked the same top solution across bootstrap samples, indicating that Metient can learn a reproducible cancer type-specific model for the melanoma and HGSOC cohorts which have only seven patients each.

These cancer type-specific parsimony metric weights lead to cohort-specific choices on how Metient ranks a patient’s Pareto front of migration histories. For example, Metient chooses the solution on the Pareto front with lowest migration number (i.e. colonizing clones) for HR-NB patient H103207 (Figure 4h), but the solution with the median value of each metric for NSCLC patient CRUK0290 (Figure 4i). To systematically assess the impact of cohort-specific rankings we computed the percentage of polyclonality and number of seeding sites in the top ranked solution for patients with each cancer type. Overall, we found a significantly higher fraction of polyclonal migrations in melanoma than HGSOC, HR-NB and NSCLC patients (Figure 4j). One explanation for this heightened polyclonality in melanoma patients is that all patients in the cohort had locoregional skin metastases, a common “in-transit” metastatic site around the primary melanoma or between the primary melanoma and regional lymph nodes. These locoregional sites could have multiple cancer cells traveling together through hematogeneous or lymphatic routes to seed new localized tumors^41^. The HR-NB and NSCLC cohorts had significantly higher percentages of metastasis-to-metastasis seeding than melanoma (Figure 4k). As described below, in the HR-NB cohort, multiple patients exhibit metastasis-to-metastasis seeding within an organ or between commonly metastatic sites. In the NSCLC cohort, 76.2% of patients have lymph node metastases, from which it is known that further metastases are commonly seeded^42^. Indeed, Metient predicted that 75% (12/16) of NSCLC patients who had metastasis-to-metastasis seeding had seeding from a lymph node to other metastases.

### Metastasis priors identify biologically relevant migration histories and alternative explanations of spread

A core advance of Metient is its ability to identify and rank the Pareto-optimal histories of a patient’s cancer. To assess how well our top ranked solution aligns with the most biologically plausible explanation, we compared our inferred migration histories to previously reported, expert-annotated seeding patterns.

Of the 167 patients analyzed, 152 patients had an expert or model-derived annotation available. Because the HR-NB annotations only indicate the presence of a migration between two sites and not the directionality, for an overall comparison of these 152 patients we compared our site-to-site migrations to those that were previously reported (i.e., a binarized representation of migration graph G (Figure 1c)). In 84% of patients (128/152), Metient-calibrate’s highest ranked solution aligns with the previously reported migration history. For the remaining 24 patients, Metient either identifies a more parsimonious history or recovers the expert annotation on the Pareto front but the metastasis priors prefer a different history than the expert. We provide a detailed case-by-case comparison in the Supplementary Information and Supplementary Figures S2, S3, S4, S5, and highlight some of the interesting cases below.

**Figure 5.**
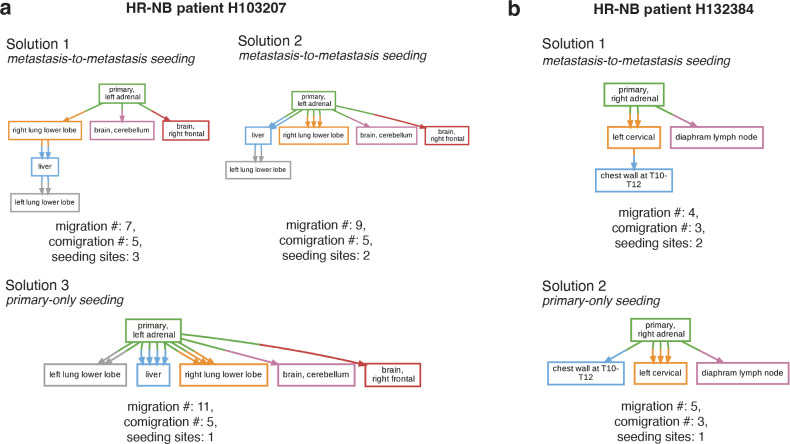
Metient finds biologically relevant trees. **(a)** All ranked Pareto-optimal migration graphs inferred by Metient-calibrate for HR-NB patient H103207. **(b)** All ranked Pareto-optimal migration graphs inferred by Metient-calibrate for HR-NB patient H132384.

Metient predicted metastasis-to-metastasis seeding for two HR-NB cases (H103207, H132384), which were previously reported to have initially seeded directly from the primary^9^. HR-NB patient H103207 shows evidence of two possible metastasis-to-metastasis seeding scenarios. One, which is ranked the highest by the calibrated parsimony metrics posits a serial progression of metastatic seeding from the primary to the right lung, then to the liver, and finally to the left lung. The other, which has the second highest rank, posits seeding from the primary to the liver and then the left lung (Figure 5a). While the exact prevalence of metastasis-to-metastasis seeding between the liver and lung in HR-NB is unknown, both are common sites of metastases across cancer types due to cancer cells’ ability to take advantage of rich blood supply, vascular organization and physiology^38^. Colonization of the lung by clones from a primary liver tumor is common^38,43,44^ and, similarly, the liver is a common site of metastasis for primary lung cancer patients^38,45^, suggesting that transitions from a liver-competent cancer clone to a lung-competent one and vice versa could also be common. For this patient, multiple colonizing clones emerge on distinct branches of the clone tree, providing another line of evidence that the suggested metastasis-to-metastasis seeding probably occurred (Supplementary Figure S2a). Specifically, the CNS-colonizing clones appear on a shared branch, and the lung- and liver-colonizing clones appear on a separate, shared branch after further primary tumor evolution occurred (Supplementary Figure S2a). This suggests that evolution within the primary tumor gave rise to multiple clones with organ-specific metastatic competence, and is concordant with the clonal analysis reported by Gundem et al.^9^ for this patient. Patient H132384 also shows evidence of metastasis-to-metastasis seeding, but from bone-to-bone, first to the left cervical and secondarily to the chest wall (Figure 5b). Metastasizing cells exhibit organ-specific genetic and phenotypic changes to survive in a new microenvironment^38^, suggesting that seeding an additional tumor within the same organ microenvironment is more likely than a secondary migration from the primary adrenal tumor in this case. In addition, prior experimental evidence shows that bone metastases prime and reprogram cells to form further secondary metastases^46,47^. These posited metastasis-to-metastasis seedings are thus upported by site proximity or organotropism, or both, and these Metient reconstructions were made without providing such information.

**Figure 6.**
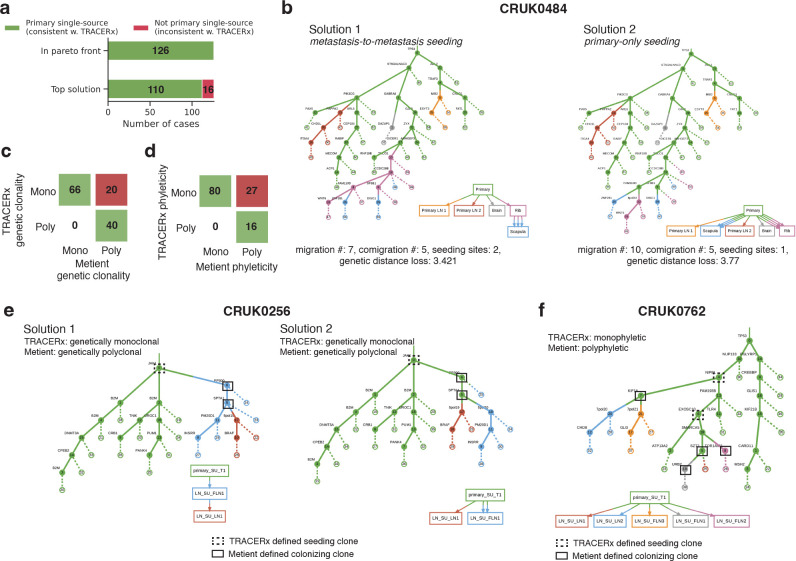
TRACERx NSCLC cohort. **(a)** The number of solutions that are classified as primary single-source (the assumed seeding pattern of TRACERx) vs. other when looking only at the Pareto-optimal solutions vs. the top solution. **(b)** The top two Pareto-optimal solutions for NSCLC patient CRUK0484 as ranked by Metient-calibrate. Comparison of Metient’s inference to TRACERx’s **(c)** clonality and **(d)** phyleticity classification. Numbers in boxes indicate the number of patients in agreement or disagreement. **(e)** All Pareto-optimal solutions for NSCLC patient CRUK0762 as ranked by Metient-calibrate. **(f)** Patient CRUK0762 where seeding pattern and clonality are in agreement between Metient-calibrate but phyleticity differs due to which clones are classified as seeding.

Next we compared the inferred migration histories from the NSCLC samples we analyzed to an in-depth analysis of the same samples by the TRACERx consortium^14^. The TRACERx analysis enforces a primary single-source dissemination model, i.e., that metastases are only seeded from the lung, for its analysis of clonality and phyleticity. While Metient generally agrees with this dissemination model, Metient predicts metastasis-to-metastasis seeding for several (12.8%; 16/126) patients (Figure 6a). CRUK0484 is one such patient where Metient proposes that an initial metastasizing clone to the rib leads to secondary metastasis formation in the scapula (Figure 6b), which we propose is a more plausible solution based on the same line of reasoning described for the bone-to-bone metastasis predicted in HR-NB patient H132384 above.

When comparing the TRACERx classifications of clonality and phyleticity for each patient to those implied by Metient’s highest-scoring solution, we find 84.1% agreement (106/126) in clonality (Figure 6c) and 78% agreement (96/123) in phyleticity (Figure 6d) (three patients classified as “mixed” phyleticity by TRACERx were excluded). The discrepancies between these classifications stem from the way in which metastatis initiating clones are defined. TRACERx identifies shared clones between a primary tumor and its metastases, defining the seeding clone as the most recent shared clone between the primary tumor and the metastasis. In contrast, Metient uses the entire migration history to define seeding clones (Methods) and accounts for metastasis-to-metastasis seeding, rather than assuming that seeding occurs only from the primary tumor. As a result, Metient has significantly higher sensitivity in detecting colonizing populations within metastases and, subsequently, increases the detection of polyclonal and polyphyletic events.

In 20 NSCLC patients, Metient inferred that multiple colonizing clones are needed to explain the full migration history, whereas no history is consistent with the TRACERx identified colonizing clones. For example, for patient CRUK0256 (Figure 6e), only the root clone is shared between primary and metastases, making it the only seeding clone by TRACERx’s definition. However, according to the clone tree and the observed presence of clone 6 in LN_SU_FLN1 and clone 5 in both LN_SU_FLN1 and LN_SU_LN1, we conclude that there must have been either a metastasis-to-metastasis seeding event (Figure 6e solution 1), or two clones originally from the primary (no longer detectable in the metastatic samples due to either ongoing evolution or undersampling) that seeded the metastases (Figure 6e solution 2). In either migration history, multiple clones had to participate in seeding in order to explain the clone tree and observed clones inferred from the sequencing data.

Inference of phyleticity is also impacted by the use of the clone tree to determine colonizing clones, as the path connecting colonizing clones is used to determine if metastatic competence arises once or multiple times during evolution. Because the number of colonizing clones is underestimated in the TRACERx analysis, monoclonal seeding is inferred more often, automatically classifying these histories as monophyletic. Furthermore, we find 27 cases where TRACERx classifies a patient as monophyletic and Metient classifies the same patient as polyphyletic; in such cases the multiple clones needed to explain seeding occur on separate paths of the clone tree (e.g. patient CRUK0762, Figure 6f). Therefore, while we agree that monophyleticity is the majority pattern in NSCLC (63%), we suggest that polyphyleticity might be underestimated due to less sensitivity in previous methods’ ability to detect colonizing clones.

## Discussion

We have presented and validated Metient, a new framework for reconstructing the migration histories of metastases. In contrast to prior work, Metient defines a Pareto front of possible migration histories, and then uses metastasis priors to resolve parsimony conflicts in a data-dependent manner. Another key innovation is that it adapts Gumbel straight-through stochastic gradient estimation to optimize the combinatorial problem required for history reconstruction. Collectively, these advances improve performance on simulated data, improve biological interpretation on real data, and define a Pareto front in a fraction of the time that MACHINA, the current state-of-the-art, takes to output a single solution. Notably, Metient uses open source software packages, whereas other methods rely on commercial MILP solvers. Metient, due to its much improved speed, could easily be adapted to much larger migration history reconstruction problems, such as those posed by single-cell data.

Here we show that by selecting among Pareto-optimal solutions using a pre-specified parsimony model and ad hoc rules, previous algorithms biased the conclusions of studies of metastatic spread. In one study^14^, primary-only seeding was assumed when analyzing migration histories, thus plausible histories with metastasis-to-metastasis seeding were ignored, even when they were identified by MACHINA. Metient thus provides an unbiased means of identifying cancer-type specific trends in metastasis biology, thus addressing a critical problem in metastasis research.

Metient’s increased precision in identifying colonizing clones allowed it to detect almost twice as much polyclonality as previously reported, suggesting that it is common for multiple clones to contribute to metastatic progression. Despite this, Metient still inferred that metastatic potential rarely emerges independently in separate evolutionary paths.

Currently, Metient uses genetic distance and organotropism as its metastasis priors, however, the Metient framework is designed to be easily extensible. Adding a new prior simply requires writing a scoring function because Metient incorporates auto-differentiation to compute its gradient updates. For instance, the framework could be easily extended to incorporate mutational signatures as a prior, since metastases exhibit shifts in mutational signature composition^48,49^.

Metient has some limitations. It scales well in compute time for larger clone trees or more samples but, because the loss landscape complexity increases substantially, in some cases (less than 1%), Metient became stuck in local minima. This problem was resolved when we ran Metient multiple times and with larger sample sizes, and we recommend this practice with larger reconstruction problems. One criteria to ensure convergence is when the Pareto front remains unchanged. Other migration history algorithms are also highly sensitive to the complexity of the loss landscape, and convergence issues that they face are not necessarily resolved by rerunning the algorithm. Also, Metient is not designed to consider subclonal copy number alternations (CNAs) when correcting its estimated variant allele frequencies for CNAs. Using the descendant cell fraction (DCF)^50^ or phylogenetic cancer cell fraction (phyloCCF)^51^ as inputs to Metient could solve this. Alternatively, one could input which clones are in which samples directly into Metient instead of the allele frequencies. Finally, we note that choice of clustering and tree inference algorithm used when inputting data into Metient can impact both the clonality and phyleticity classifications. In an attempt to most accurately compare our migration histories to previously reported results, where possible, we use the same clustering and trees inferred for the original datasets.

In conclusion, we show that Metient offers a fast and adaptable, fully automated framework that leverages bulk DNA sequencing data to probe enduring questions in metastasis research.

## Methods

### Estimating observed clone proportions

The first step of Metient is to estimate the binary presence or absence of clone tree (T) nodes in each site. The clone tree T can either be provided as input, or inferred from the DNA sequencing data using, e.g., Orchard^52^, PairTree^53^, SPRUCE^54^, CITUP^55^, or EXACT^56^. Building on a previous approach as described by Wintersinger et al.^53^, Metient estimates the proportion of clones in each site using the input clone tree T and read count data from bulk DNA sequencing. For a genomic locus j in anatomical site k, the probability of observing read count data $x_{kj}$ is defined using the following:

- $A_{kj}$ is the number of reads that map to genomic locus j in anatomical site k with the variant allele
- $R_{kj}$ is the number of reads that map to genomic locus j in anatomical site k with the reference allele
- $\omega_{kj}$ is a conversion factor from mutation cellular frequency to variant allele frequency (VAF) for genomic locus j in anatomical site k

Using a binomial model, we then estimate the proportion of anatomical site k containing clone c using $p\left(x_{kj}|F_{kj}\right)=Binom\left(A_{kj}|A_{kj}+R_{kj},\omega_{kj}F_{kj}\right)$. Where F=UB is the mutation cellular frequency matrix, $B\in \{0,\;1{\}}^{C\;\times \;M}$ is 1:1 with a clone tree, where C is the number of clones and M is the number of mutations or mutation clusters, and $B_{cm}=1$ if clone c contains mutation m (Figure 1b). $U\in [0,\;1{]}^{K\times C}$, where K is the number of anatomical sites, and $U_{kc}$ is the fraction of anatomical site k made up by clone c (Figure 1b). An L1 regularization is used to promote sparsity, since we expect most values in U to be zero. For details on how to set $\omega_{kj}$, see “Variant read probability calculation (ω)” in Supplementary Information. An alternative way to find a point estimate of U is using a previously described projection algorithm for this problem^52,53,56,57^. A point estimate U can be found by optimizing the following quadratic approximation to the binomial likelihood of U given B and F:

(1) $$LP(U|B,F,W)={min}_{\hat{F},U}\;∥W⊙(F-\overset{ˆ}{F}){∥}^{2}s.t.U1\leq 1,U\geq 0,\overset{ˆ}{F}=UB$$

where ‖·‖ is the Frobenius norm, 1 is a vector of 1s, F are the observed mutation frequencies, W is a K×M matrix of inverse-variances for each mutation in each sample derived from F, and ⊙ is the Hadamard, i.e., element-wise product. The definition for W is as described in previous work^53,56^.

We use U (estimated in either of the previously described ways) to determine if a clone c is present in an anatomical site k. If c is present, we attach a witness node with label k (leaf nodes connected by dashed lines in Figure 1b, c) to clone c in clone tree T. We deem c to be present in k if $U_{kc}>5\%$ for a given anatomical site k and clone c. If a clone c does not make up 5% of any of the K anatomical sites, and c is a leaf node of the clone tree T, we remove this node since it is not well estimated by the data.

Here the term “anatomical site” is used to describe a distinct tumor mass. If multiple samples are taken from the same tumor mass, we combine them as described in “Bulk DNA sequencing pre-processing: Non-small Cell Lung Cancer Dataset”.

Note that read count data are only used to determine which clones are present in which sites, if a matrix indicating the presence or absence of each clone in each anatomical site is available, it can be used as an input to replace the read count data. These clone-to-site assignment matrices can be derived, e.g., from single-cell data.

### Labeling the clone tree

The next step in inferring a migration history is to jointly infer a labeling of the clone tree and resolve polytomies, i.e., nodes with more than two children. Polytomy resolution is discussed in the section “Resolving polytomies”.

Because we are interested in identifying multiple hypotheses of metastatic spread, Metient seeks to find multiple possible labelings of a clone tree T. Each possible labeling is represented by a matrix $V\in \{0,\;1{\}}^{K\;\times \;C}$, where K is the number of anatomical sites and C is the number of clones, and $V_{kc}=1$ if clone c is first detected in anatomical site k. Each column of V is a one-hot vector. We solve for an individual V by optimizing the evidence lower bound, or ELBO, as defined by:

(2) $$ELBO(q)=E_{q(V)}[log\;p(U,T,V)]+H(V)$$

Where $E_{q(V)}[log\;p(U,T,V)]$ evaluates a labeling based on parsimony, genetic distance, and organotropism, and the second term is the entropy term. U has been optimized as described in the previous section “Estimating observed clone proportions”, or taken as input from the user. See Supplementary Information for a full derivation of this objective. Because V is a matrix of discrete categorical variables, we do not optimize V directly, but rather the underlying probabilites of each category that we optimize using a Gumbel-softmax estimator (see “Gumbel-softmax optimization”).

### Gumbel-softmax optimization

In the previous section, we described how to score the matrix representation of the labeled clone tree, V. Here, we describe how to optimize V via the straight-through estimator of the Gumbel-Softmax distribution^23,24^. Starting with a matrix $\psi \in \{0,\;1{\}}^{K\;\times \;C}$, of randomly initialized values, where K is the number of anatomical sites and C is the number of clones, and each column represents the unnormalized log probabilities of clone c being labeled in site k:

1. At every iteration, for each clone c, we sample $g_{1c}\ldots g_{kc},\;k$ i.i.d. samples from Gumbel(0,1) and compute $y_{\mathrm{ic}}=\psi_{\mathrm{ic}}+g_{\mathrm{ic}}$.
2. We then sample from the categorical distribution represented by the column vector $\psi_{:c}$ by setting $i^{*}={argmax}_{i}y_{ic}$ and represent that sample with a one-hot encoding in V, i.e., $V_{ic}=1$ if $i=i^{*}$, 0 otherwise.
3. Then we evaluate the ELBO(ν) where
$$\nu_{ic}=\frac{exp\left(y_{ic}/\tau \right)}{\sum_{j=1}^{k}\;exp\left(y_{jc}/\tau \right)}\;\text{for}\;i=1,\ldots ,k,$$
  using a stochastic approximation based on V, and take the gradient of this ELBO in the backward pass, thus implementing the straight-through estimator.
4. During training, start with a high τ to permit exploration, then gradually anneal τ to a small but non-zero value so that the Gumbel-Softmax distribution, ν resembles a one-hot vector.

At the end of training, as τ approaches 0, then the gradient becomes unbiased and ν approaches V. In order to capture multiple modes of the posterior distribution, each representing different hypotheses about the migration history, we optimize multiple Vs in parallel. To do this, we set up steps 1–3 such that xψs are solved for in parallel^58^ (with a different random initialization for each parallel process), where x is equal to the sample size and is calculated according to the size of the inputs $\left(\propto K^{C}\right)$. See Supplementary Information for further explanation.

### Resolving polytomies

An overview of the algorithm to resolve polytomies is given in Supplementary Figure S7a and b.

1. If a node i in T has more than 2 children, we create a new “resolver” node for every site where either i or i’s children are observed in. Specifically, for every node i in T, we look at the set of nodes P, which contains node i and node i’s children. We then tally the anatomical sites of all witness nodes for nodes in P. If any anatomical site is counted at least twice, a resolver node with that anatomical site label is added as a new child of i. The genetic distance between the parent node i and its new resolver node is set to 0 since there are no observed mutations between the two nodes.
2. We allow the children of i to stay as a child of i, or become a child of one of the resolver nodes of i.
3. Any resolver nodes that are unused (i.e. have no children) or which do not improve the migration history (i.e.the parsimony metrics without the resolver node are the same or worse) are removed.

### Fixing optimal subtrees

To improve convergence, we perform two rounds of optimization when solving for a labeled clone tree and resolving polytomies:

1. Solve for labeled trees and resolve polytomies jointly (as described in previous sections).
2. For each pair of labeled tree and polytomy resovled tree, find optimal subtrees. I.e., find the largest subtrees, as defined by the most number of nodes, where all labels for all nodes are equal. This means that there is no other possible optimal labeling for this subtree (there are 0 migrations, 0 comigrations, 0 seeding sites), and we can keep it fixed. Fix these nodes’ labelings and adjacency matrix connections (if using polytomy resolution).
3. Repeat step 1 for any nodes that have not been fixed in step 2.

### Metient-calibrate

In Metient-calibrate, we aim to fit a patient cohort-specific parsimony model using the metastasis priors. To score a migration history using genetic distance, we use the following equation: $\sum_{ij}\;-log\left(D_{ij}\right)K_{ij}$, where D contains the normalized number of mutations between clones, and K=1 if clone i is the parent of clone j and clone i and clone j have different anatomical site labels.

To score a migration history using organotropism, we use the following equation: $\sum_{i=1}^{K}\;-log\left(o_{i}\right)g_{i}$, where vector o contains the frequency at which the primary seeds other anatomical sites, and vector g contains the number of migrations from the primary site to all other anatomical sites for a particular migration history.

To optimize the parsimony metric weights, Metient identifies a Pareto front of labeled trees for each patient and scores these trees based on (1) the weighted parsimony metrics and (2) the metastasis priors: genetic distance and, if appropriate anatomical labels are available, organotropism. These form the parsimony distribution and metastasis prior distribution, respectively. We initialize with equal weights and use gradient descent to minimize the cross entropy loss between the parsimony distribution and metastasis prior distribution for all patients in the cohort. Once the optimization converges, Metient rescores the trees on the Pareto front using the fitted weights, to identify the maximum calibrated parsimony solution, and genetic distance and organotropism are used to break ties between equally parsimonious migration histories. See Supplementary Information for a more detailed derivation.

### Metient-evaluate

In Metient-evaluate, weights for each maximum parsimony metric (migrations, comigrations, seeding sites) and optionally, genetic distance and organotropism, are taken as input. These weights are used to rank the solutions on the Pareto front. If no weights are inputted, we provide a pan-cancer parsimony model calibrated to the four cohorts (melanoma, HGSOC, HR-NB, NSCLC) discussed in this work.

### Defining the organotropism matrix

Data from the MSK-MET study^29^ for 25,775 patients with annotations of distant metastases locations was downloaded from the publicly available cbioportal^59^. Each patient had annotations of one of 27 primary cancer types and the presence or absence of a metastasis in one of 21 distant anatomical sites. The original authors extracted this data from electronic health records and mapped it to a reference set of anatomical sites. We sum over all patients to build a 27 × 21, cancer type by metastatic site occurrence matrix. We then normalize the rows to turn these into frequencies. We interpret the negative log frequencies as a “relative time to metastasis”, and only score migrations from the primary site to other sites, because there is no data to indicate frequencies of seeding from metastatic sites to other metastatic sites, or back to the primary. We make this data available for users, with the option for users to instead input their own organotropism vector for each patient.

### Evaluations on simulated data

We use the simulated data for 80 patients provided by MACHINA^17^ to benchmark our method’s performance. To prepare inputs to Metient, we use the same clustering algorithm and clone tree inference algorithm used in MACHINA (MACHINA^17^ and SPRUCE^54^, respectively) in order to accurately compare only our migration history inference algorithm (including polytomy resolution) against MACHINA’s. All performance scores are reported using MACHINA’s PMH-TI mode and Metient-calibrate with a sample size of 1024, both with default configurations. We do not use polytomy resolution for Metient-calibrate in these results, since it does not improve performance on simulated data. (Supplementary Tables 4, 3). However, this performance is not necessarily indicative of polytomy resolution working poorly, because it actually finds more parsimonious solutions than the ground truth solution in 75% of simulated data (Supplementary Figure S6).

#### Evaluation metrics.

We use the same migration graph and seeding clones F1-scores as MACHINA. Given a reconstructed migration graph G, its recall and precision with respect to the ground truth migration graph $G^{*}$ are calculated as follows:

$$\text{recall}\;=\frac{\left|E(G)\;\cap \;E(G^{*})\right|}{\left|E(G^{*})\right|}\;\text{precision}\;=\frac{\left|E(G)\;\cap \;E(G^{*})\right|}{\left|E(G)\right|}$$

where E(G) are the edges of G, and multiple edges between the same two sites are included in E(G). When there are multiple edges from site i to site $j,\;\left|E(G)\cap E(G^{*})\right|=min(a,b)$, where a and b are the number of edges from site i to site j in G and $G^{*}$, respectively.

Recall and precision of the seeding clones in the inferred migration history (which includes inference of both the clone tree labeling and observed clone proportions) is calculated as follows:

$$\text{recall}\;=\frac{\left|C(U,\;V)\;\cap \;C(U^{*},\;V^{*})\right|}{\left|C(U^{*},\;V^{*})\right|}\;\text{precision}\;=\frac{\left|C(U,\;V)\;\cap \;C(U^{*},\;V^{*})\right|}{\left|C(U,\;V)\right|}$$

where C(U,V) is the set of mutations, i.e., the subclone, associated with the clone nodes that have an outgoing migration edge. For example, C(U,V)=A,B,C in solution A of Figure 1c. The definition for seeding clones used in these evaluations is distinct from how we define seeding clones in the rest of the paper (“Defining colonizing clones, clonality, and phyleticity” in Methods). Specifically, if there is an edge between two nodes (u,v), where the labeling of u and v are not equal, we define the seeding clone as v. However in order to consistently compare to MACHINA in these evaluations, we use their definition and define the seeding clone as u. We note that identifying the mutations of v is generally a harder problem.

#### Timing benchmarks.

All timing benchmarks (Figure 2e) were run on 8 Intel(R) Xeon(R) CPU E5-2697 v4 @ 2.30GHz CPU cores with 8 gigabytes of RAM per core. Runtime of each method is the time needed to run inference and save dot files of the inferred migration histories (and for Metient, an additional serialized file with the results of the top k migration histories). We compare MACHINA’s PMH-TI mode to Metient-calibrate with a sample size of 1024, both with default configurations. These are the same modes used to report comparisons in F1-scores. Each value in Figure 2e is the time needed to run one patient’s tree. Because Metient-calibrate has an additional inference step where parsimony metric weights are fit to a cohort, we take the time needed for this additional step and divide it by the number of patient trees in the cohort, and add this time to each patient’s migration history runtime.

### Defining colonizing clones, clonality, and phyleticity

A colonizing clone is defined as a node in a migration history whose parent is a different color than itself. There are two exceptions to this rule: when node a has a parent with a different color than itself, but the node is a witness node (Figure 1c) or a polytomy resolver node (e.g. A_POL in Supplementary Figure S7a). In these cases, these nodes do not represent any new mutations, but rather contain the same mutations as its parent. For these two cases, the colonizing clone is defined to be a’s parent node.

In order to rectify different meanings of the terms “monoclonal” and “polyclonal” used in previous work, we define two terms:

- genetic clonality: if all sites are seeded by the same colonizing clone, this patient is genetically monoclonal, otherwise, genetically polyclonal.
- site clonality: if each site is seeded by one colonizing clone, but not necessarily the same colonizing clone, this patient is site monoclonal, otherwise, site polyclonal.

Genetic clonality and site clonality are depicted schematically in Figure 4b.

To define phyleticity, we first extract all colonizing clones from a migration history. We then identify the colonizing clone closest to the root, s, i.e., the colonizing clone with the shortest path to the root. If all other colonizing clones are descendants of the tree rooted at s, the migration history is monophyletic, otherwise, it is polyphyletic. Under this definition, if a tree is monophyletic, then there are no independent evolutionary trajectories that give rise to colonizing clones. This is depicted schematically in Figure 4c.

In order to accurately compare our phyleticity measurements to TRACERx, we use their definition in Figure 6c and the TRACERx comparison analysis. To apply their definition to our migration histories, we extract colonizing clones as described above, and then determine if there is a Hamiltonian path in the clone tree that connects the colonizing clones. I.e., we determine if there is a path in the clone tree that visits each colonizing clone exactly once. If such a Hamiltonian path exists, we call this migration history monophyletic under the TRACERx definition, and polyphyletic otherwise.

### Bootstrap sampling for fitting parsimony metric weights

Running Metient-calibrate on the 167 patients from the melanoma, HGSOC, HR-NB and NSCLC datasets infers a Pareto front of migration histories for each patient. For each dataset, we subset patients that have a Pareto front with size greater than one, and take 100 bootstrap samples of patients from this subset. Patients with a single solution on the Pareto front do not have an impact on the cross-entropy loss used to fit the parsimony metric weights. For each bootstrap sample of patients, their Pareto front migration histories are used to fit the parsimony metric weights (“Calibrate alignment” in Supplementary Information). For each of the parsimony metric weights fit to a bootstrap sample, we evaluated how these weights would order the Pareto front, and evaluated how consistently the same top solution was chosen. We average the percent of times the same solution is ranked as the top solution across the four datasets.

## Supplementary Material

Supplement 1

## Data Availability

The HR-NB dataset was accessed from the NCI’s Cancer Research Data Commons (https://datacommons.cancer.gov) under the study phs03111.v1.p1. The anatomical site labels for TRACERx patients used data generated by The TRAcking Non-small Cell Lung Cancer Evolution Through Therapy (Rx) (TRACERx) Consortium and provided by the UCL Cancer Institute and The Francis Crick Institute. The TRACERx study is sponsored by University College London, funded by Cancer Research UK and coordinated through the Cancer Research UK and UCL Cancer Trials Centre. The organotropism matrix derived from MSK-MET is available at https://github.com/morrislab/metient/blob/main/metient/data/msk_met/msk_met_freq_by_cancer_type.csv. The following publicly available datasets were used: melanoma^3^, breast^20^, HGSOC^4^, NSCLC^14^, MSK-MET^29^.

## References

[1] Ganesh Karuna and Massagué Joan. Targeting metastatic cancer. Nature medicine, 27(1):34–44, 2021. 10.1038/s41591-020-01195-4 PMC7895475 33442008

[2] Gundem Gunes, Van Loo Peter, Kremeyer Barbara, Alexandrov Ludmil B, Tubio Jose MC, Papaemmanuil Elli, Brewer Daniel S, Kallio Heini ML, Högnäs Gunilla, Annala Matti, The evolutionary history of lethal metastatic prostate cancer. Nature, 520(7547):353–357, 2015. 25830880 10.1038/nature14347 PMC4413032

[3] Zachary Sanborn J, Chung Jongsuk, Purdom Elizabeth, Wang Nicholas J, Kakavand Hojabr, Wilmott James S, Butler Timothy, Thompson John F, Mann Graham J, Haydu Lauren E, Phylogenetic analyses of melanoma reveal complex patterns of metastatic dissemination. Proceedings of the National Academy of Sciences, 112(35):10995–11000, 2015. 10.1073/pnas.1508074112 PMC4568214 26286987

[4] McPherson Andrew, Roth Andrew, Laks Emma, Masud Tehmina, Bashashati Ali, Zhang Allen W, Ha Gavin, Biele Justina, Yap Damian, Wan Adrian, Divergent modes of clonal spread and intraperitoneal mixing in high-grade serous ovarian cancer. Nature genetics, 48(7):758–767, 2016. 27182968 10.1038/ng.3573

[5] Birkbak Nicolai J and McGranahan Nicholas. Cancer genome evolutionary trajectories in metastasis. Cancer cell, 37(1):8–19, 2020. 31935374 10.1016/j.ccell.2019.12.004

[6] Wei Q, Ye Z, Zhong X, Li L, Wang C, Myers RE, Palazzo JP, Fortuna D, Yan A, Waldman SA, Multiregion whole-exome sequencing of matched primary and metastatic tumors revealed genomic heterogeneity and suggested polyclonal seeding in colorectal cancer metastasis. Annals of oncology, 28(9):2135–2141, 2017. 28911083 10.1093/annonc/mdx278 PMC5834069

[7] Hu Zheng, Ding Jie, Ma Zhicheng, Sun Ruping, Seoane Jose A, Shaffer J Scott, Suarez Carlos J, Berghoff Anna S, Cremolini Chiara, Falcone Alfredo, Quantitative evidence for early metastatic seeding in colorectal cancer. Nature genetics, 51(7):1113–1122, 2019. 31209394 10.1038/s41588-019-0423-x PMC6982526

[8] Hu Zheng, Li Zan, Ma Zhicheng, and Curtis Christina. Multi-cancer analysis of clonality and the timing of systemic spread in paired primary tumors and metastases. Nature genetics, 52(7):701–708, 2020. 32424352 10.1038/s41588-020-0628-z PMC7343625

[9] Gundem Gunes, Levine Max F, Roberts Stephen S, Cheung Irene Y, Medina-Martínez Juan S, Feng Yi, Arango-Ossa Juan E, Chadoutaud Loic, Rita Mathieu, Asimomitis Georgios, Clonal evolution during metastatic spread in high-risk neuroblastoma. Nature Genetics, pages 1–12, 2023. 10.1038/s41588-023-01395-x PMC11481711 37169874

[10] Brown David, Smeets Dominiek, Székely Borbála, Larsimont Denis, Szász A Marcell, Adnet Pierre-Yves, Rothé Françoise, Rouas Ghizlane, Nagy Zsófia I, Faragó Zsófia, Phylogenetic analysis of metastatic progression in breast cancer using somatic mutations and copy number aberrations. Nature communications, 8(1):14944, 2017. 10.1038/ncomms14944 PMC5474888 28429735

[11] Brastianos Priscilla K, Carter Scott L, Santagata Sandro, Cahill Daniel P, Taylor-Weiner Amaro, Jones Robert T, Van Allen Eliezer M, Lawrence Michael S, Horowitz Peleg M, Cibulskis Kristian, Genomic characterization of brain metastases reveals branched evolution and potential therapeutic targets. Cancer discovery, 5(11):1164–1177, 2015. 26410082 10.1158/2159-8290.CD-15-0369 PMC4916970

[12] Turajlic Samra, Xu Hang, Litchfield Kevin, Rowan Andrew, Chambers Tim, Lopez Jose I, Nicol David, O’Brien Tim, Larkin James, Horswell Stuart, Tracking cancer evolution reveals constrained routes to metastases: Tracerx renal. Cell, 173(3):581–594, 2018. 29656895 10.1016/j.cell.2018.03.057 PMC5938365

[13] Noorani Ayesha, Li Xiaodun, Goddard Martin, Crawte Jason, Alexandrov Ludmil B, Secrier Maria, Eldridge Matthew D, Bower Lawrence, Weaver Jamie, Lao-Sirieix Pierre, Genomic evidence supports a clonal diaspora model for metastases of esophageal adenocarcinoma. Nature genetics, 52(1):74–83, 2020. 31907488 10.1038/s41588-019-0551-3 PMC7100916

[14] Bakir Maise Al, Huebner Ariana, Martínez-Ruiz Carlos, Grigoriadis Kristiana, Watkins Thomas B. K., Pich Oriol, Moore David A., Veeriah Selvaraju, Ward Sophia, Laycock Joanne, and The evolution of non-small cell lung cancer metastases in tracerx. Nature, Apr 2023. 10.1038/s41586-023-05729-x PMC10115651 37046095

[15] Dang HX, White BS, Foltz SM, Miller CA, Luo Jingqin, Fields RC, and Maher CA. Clonevol: clonal ordering and visualization in cancer sequencing. Annals of oncology, 28(12):3076–3082, 2017. 28950321 10.1093/annonc/mdx517 PMC5834020

[16] Reiter Johannes G, Makohon-Moore Alvin P, Gerold Jeffrey M, Bozic Ivana, Chatterjee Krishnendu, Iacobuzio-Donahue Christine A, Vogelstein Bert, and Nowak Martin A. Reconstructing metastatic seeding patterns of human cancers. Nature communications, 8(1):14114, 2017. 10.1038/ncomms14114 PMC5290319 28139641

[17] El-Kebir Mohammed, Satas Gryte, and Raphael Benjamin J. Inferring parsimonious migration histories for metastatic cancers. Nature genetics, 50(5):718–726, 2018. 29700472 10.1038/s41588-018-0106-z PMC6103651

[18] Zhang Chong, Zhang Lin, Xu Tianlei, Xue Ruidong, Yu Liang, Zhu Yuelu, Wu Yunlong, Zhang Qingqing, Li Dongdong, Shen Shuohao, Mapping the spreading routes of lymphatic metastases in human colorectal cancer. Nature communications, 11(1):1993, 2020. 10.1038/s41467-020-15886-6 PMC7181746 32332722

[19] Naxerova Kamila, Reiter Johannes G, Brachtel Elena, Lennerz Jochen K, Van De Wetering Marc, Rowan Andrew, Cai Tianxi, Clevers Hans, Swanton Charles, Nowak Martin A, Origins of lymphatic and distant metastases in human colorectal cancer. Science, 357(6346):55–60, 2017. 28684519 10.1126/science.aai8515 PMC5536201

[20] Hoadley Katherine A, Siegel Marni B, Kanchi Krishna L, Miller Christopher A, Ding Li, Zhao Wei, He Xiaping, Parker Joel S, Wendl Michael C, Fulton Robert S, Tumor evolution in two patients with basal-like breast cancer: a retrospective genomics study of multiple metastases. PLoS medicine, 13(12):e1002174, 2016. 27923045 10.1371/journal.pmed.1002174 PMC5140046

[21] Gurobi Optimization, LLC. Gurobi Optimizer Reference Manual, 2023.

[22] Stiglitz Joseph E. Pareto optimality and competition. The Journal of Finance, 36(2):235–251, 1981.

[23] Jang Eric, Gu Shixiang, and Poole Ben. Categorical reparameterization with gumbel-softmax. arXiv preprint arXiv:1611.01144, 2016.

[24] Maddison Chris J, Mnih Andriy, and Teh Yee Whye. The concrete distribution: A continuous relaxation of discrete random variables. arXiv preprint arXiv:1611.00712, 2016.

[25] Lengyel Ernst. Ovarian cancer development and metastasis. The American journal of pathology, 177(3):1053–1064, 2010. 20651229 10.2353/ajpath.2010.100105 PMC2928939

[26] Mitra Anirban K. Ovarian cancer metastasis: a unique mechanism of dissemination. IntechOpen, 2016.

[27] Gui Philippe and Bivona Trever G. Evolution of metastasis: New tools and insights. Trends in Cancer, 8(2):98–109, 2022. 34872888 10.1016/j.trecan.2021.11.002

[28] Reticker-Flynn Nathan E, Zhang Weiruo, Belk Julia A, Basto Pamela A, Escalante Nichole K, Pilarowski Genay OW, Bejnood Alborz, Martins Maria M, Kenkel Justin A, Linde, Lymph node colonization induces tumor-immune tolerance to promote distant metastasis. Cell, 185(11):1924–1942, 2022. 35525247 10.1016/j.cell.2022.04.019 PMC9149144

[29] Nguyen Bastien, Fong Christopher, Luthra Anisha, Smith Shaleigh A, DiNatale Renzo G, Nandakumar Subhiksha, Walch Henry, Chatila Walid K, Madupuri Ramyasree, Kundra Ritika, Genomic characterization of metastatic patterns from prospective clinical sequencing of 25,000 patients. Cell, 185(3):563–575, 2022. 35120664 10.1016/j.cell.2022.01.003 PMC9147702

[30] Williams Marc J, Werner Benjamin, Barnes Chris P, Graham Trevor A, and Sottoriva Andrea. Identification of neutral tumor evolution across cancer types. Nature genetics, 48(3):238–244, 2016. 26780609 10.1038/ng.3489 PMC4934603

[31] Taddei François, Radman Miroslav, Maynard-Smith John, Toupance Bruno, Gouyon Pierre-Henri, and Godelle Bernard. Role of mutator alleles in adaptive evolution. Nature, 387(6634):700–702, 1997. 9192893 10.1038/42696

[32] Mao Emily F, Lane Laura, Lee Jean, and Miller Jeffrey H. Proliferation of mutators in a cell population. Journal of bacteriology, 179(2):417–422, 1997. 8990293 10.1128/jb.179.2.417-422.1997 PMC178711

[33] Gentile Christopher F, Yu Szi-Chieh, Serrano Sebastian Akle, Gerrish Philip J, and Sniegowski Paul D. Competition between high-and higher-mutating strains of escherichia coli. Biology letters, 7(3):422–424, 2011. 21227974 10.1098/rsbl.2010.1036 PMC3097864

[34] Turajlic Samra and Swanton Charles. Metastasis as an evolutionary process. Science, 352(6282):169–175, 2016. 27124450 10.1126/science.aaf2784

[35] Martínez-Jiménez Francisco, Movasati Ali, Brunner Sascha Remy, Nguyen Luan, Priestley Peter, Cuppen Edwin, and Van Hoeck Arne. Pan-cancer whole-genome comparison of primary and metastatic solid tumours. Nature, pages 1–9, 2023. 10.1038/s41586-023-06054-z PMC10247378 37165194

[36] Sansregret Laurent and Swanton Charles. The role of aneuploidy in cancer evolution. Cold Spring Harbor perspectives in medicine, 7(1):a028373, 2017. 28049655 10.1101/cshperspect.a028373 PMC5204330

[37] Christensen Ditte S, Ahrenfeldt Johanne, Sokač Mateo, Kisistók Judit, Thomsen Martin K, Maretty Lasse, McGranahan Nicholas, and Birkbak Nicolai J. Treatment represents a key driver of metastatic cancer evolution. Cancer Research, 82(16):2918–2927, 2022. 35731928 10.1158/0008-5472.CAN-22-0562

[38] Gao Yang, Bado Igor, Wang Hai, Zhang Weijie, Rosen Jeffrey M, and Zhang Xiang H-F. Metastasis organotropism: redefining the congenial soil. Developmental cell, 49(3):375–391, 2019. 31063756 10.1016/j.devcel.2019.04.012 PMC6506189

[39] Alvord Ellsworth C. Why do gliomas not metastasize? Archives of Neurology, 33(2):73–75, 1976. 1252152 10.1001/archneur.1976.00500020001001

[40] Kumar Sudhir, Chroni Antonia, Tamura Koichiro, Sanderford Maxwell, Oladeinde Olumide, Aly Vivian, Vu Tracy, and Miura Sayaka. Pathfinder: Bayesian inference of clone migration histories in cancer. Bioinformatics, 36(Supplement_2):i675–i683, 2020. 33381835 10.1093/bioinformatics/btaa795 PMC7773489

[41] Wolf Ingrid H, Richtig Erika, Kopera Daisy, and Kerl Helmut. Locoregional cutaneous metastases of malignant melanoma and their management. Dermatologic surgery, 30:244–247, 2004. 14871216 10.1111/j.1524-4725.2004.30091.x

[42] Sleeman Jonathan, Schmid Anja, and Thiele Wilko. Tumor lymphatics. In Seminars in cancer biology, volume 19, pages 285–297. Elsevier, 2009. 19482087 10.1016/j.semcancer.2009.05.005

[43] Lee Yeu-Tsu Margaret and Geer Deborah A. Primary liver cancer: pattern of metastasis. Journal of surgical oncology, 36(1):26–31, 1987. 3041113 10.1002/jso.2930360107

[44] Wu Wenrui, He Xingkang, Andayani Dewi, Yang Liya, Ye Jianzhong, Li Yating, Chen Yanfei, and Li Lanjuan. Pattern of distant extrahepatic metastases in primary liver cancer: a seer based study. Journal of Cancer, 8(12):2312, 2017. 28819435 10.7150/jca.19056 PMC5560150

[45] Riihimäki Matias, Hemminki A, Fallah Mahdi, Thomsen Hauke, Sundquist Kristina, Sundquist Jan, and Hemminki Kari. Metastatic sites and survival in lung cancer. Lung cancer, 86(1):78–84, 2014. 25130083 10.1016/j.lungcan.2014.07.020

[46] Bado Igor L, Zhang Weijie, Hu Jingyuan, Xu Zhan, Wang Hai, Sarkar Poonam, Li Lucian, Wan Ying-Wooi, Liu Jun, Wu William, The bone microenvironment increases phenotypic plasticity of er+ breast cancer cells. Developmental cell, 56(8):1100–1117, 2021. 33878299 10.1016/j.devcel.2021.03.008 PMC8062036

[47] Zhang Weijie, Bado Igor L, Hu Jingyuan, Wan Ying-Wooi, Wu Ling, Wang Hai, Gao Yang, Jeong Hyun-Hwan, Xu Zhan, Hao Xiaoxin, The bone microenvironment invigorates metastatic seeds for further dissemination. Cell, 184(9):2471–2486, 2021. 33878291 10.1016/j.cell.2021.03.011 PMC8087656

[48] Ashley Charles W, Da Cruz Paula Arnaud, Kumar Rahul, Mandelker Diana, Pei Xin, Riaz Nadeem, Reis-Filho Jorge S, and Weigelt Britta. Analysis of mutational signatures in primary and metastatic endometrial cancer reveals distinct patterns of dna repair defects and shifts during tumor progression. Gynecologic oncology, 152(1):11–19, 2019. 30415991 10.1016/j.ygyno.2018.10.032 PMC6726428

[49] Angus Lindsay, Smid Marcel, Wilting Saskia M, van Riet Job, Van Hoeck Arne, Nguyen Luan, Nik-Zainal Serena, Steenbruggen Tessa G, Tjan-Heijnen Vivianne CG, Labots Mariette, The genomic landscape of metastatic breast cancer highlights changes in mutation and signature frequencies. Nature genetics, 51(10):1450–1458, 2019. 31570896 10.1038/s41588-019-0507-7 PMC6858873

[50] Satas Gryte, Zaccaria Simone, El-Kebir Mohammed, and Raphael Benjamin J. Decifering the elusive cancer cell fraction in tumor heterogeneity and evolution. Cell systems, 12(10):1004–1018, 2021. 34416171 10.1016/j.cels.2021.07.006 PMC8542635

[51] Jamal-Hanjani Mariam, Wilson Gareth A, McGranahan Nicholas, Birkbak Nicolai J, Watkins Thomas BK, Veeriah Selvaraju, Shafi Seema, Johnson Diana H, Mitter Richard, Rosenthal Rachel, Tracking the evolution of non–small-cell lung cancer. New England Journal of Medicine, 376(22):2109–2121, 2017. 28445112 10.1056/NEJMoa1616288

[52] Kulman Ethan, Kuang Rui, and Morris Quaid. Orchard: building large cancer phylogenies using stochastic combinatorial search. arXiv preprint arXiv:2311.12917, 2023. 10.1371/journal.pcbi.1012653 PMC11723595 39775053

[53] Wintersinger Jeff A, Dobson Stephanie M, Kulman Ethan, Stein Lincoln D, Dick John E, and Morris Quaid. Reconstructing complex cancer evolutionary histories from multiple bulk dna samples using pairtreereconstructing cancer evolutionary histories using pairtree. Blood Cancer Discovery, pages OF1–OF12, 2022. 10.1158/2643-3230.BCD-21-0092 PMC9780082 35247876

[54] El-Kebir Mohammed, Satas Gryte, Oesper Layla, and Raphael Benjamin J. Inferring the mutational history of a tumor using multi-state perfect phylogeny mixtures. Cell systems, 3(1):43–53, 2016. 27467246 10.1016/j.cels.2016.07.004

[55] Malikic Salem, McPherson Andrew W, Donmez Nilgun, and Sahinalp Cenk S. Clonality inference in multiple tumor samples using phylogeny. Bioinformatics, 31(9):1349–1356, 2015. 25568283 10.1093/bioinformatics/btv003

[56] Ray Surjyendu, Jia Bei, Safavi Sam, van Opijnen Tim, Isberg Ralph, Rosch Jason, and Bento José. Exact inference under the perfect phylogeny model. arXiv preprint arXiv:1908.08623, 2019.

[57] Jia Bei, Ray Surjyendu, Safavi Sam, and Bento José. Efficient projection onto the perfect phylogeny model. Advances in Neural Information Processing Systems, 31, 2018.

[58] Li Yaoxin, Liu Jing, Lin Guozheng, Hou Yueyuan, Mou Muyun, and Zhang Jiang. Gumbel-softmax-based optimization: a simple general framework for optimization problems on graphs. Computational Social Networks, 8(1):1–16, 2021.

[59] Gao Jianjiong, Aksoy Bülent Arman, Dogrusoz Ugur, Dresdner Gideon, Gross Benjamin, Sumer S Onur, Sun Yichao, Jacobsen Anders, Sinha Rileen, Larsson Erik, Integrative analysis of complex cancer genomics and clinical profiles using the cbioportal. Science signaling, 6(269):pl1–pl1, 2013. 23550210 10.1126/scisignal.2004088 PMC4160307

[60] Sankoff David. Minimal mutation trees of sequences. SIAM Journal on Applied Mathematics, 28(1):35–42, 1975.

[61] Tarabichi Maxime, Salcedo Adriana, Deshwar Amit G, Leathlobhair Máire Ni, Wintersinger Jeff, Wedge David C, Van Loo Peter, Morris Quaid D, and Boutros Paul C. A practical guide to cancer subclonal reconstruction from dna sequencing. Nature methods, 18(2):144–155, 2021. 33398189 10.1038/s41592-020-01013-2 PMC7867630

[62] Roth Andrew, Khattra Jaswinder, Yap Damian, Wan Adrian, Laks Emma, Biele Justina, Ha Gavin, Aparicio Samuel, Bouchard-Côté Alexandre, and Shah Sohrab P. Pyclone: statistical inference of clonal population structure in cancer. Nature methods, 11(4):396–398, 2014. 24633410 10.1038/nmeth.2883 PMC4864026

[63] Gillis Sierra and Roth Andrew. Pyclone-vi: scalable inference of clonal population structures using whole genome data. BMC bioinformatics, 21:1–16, 2020. 33302872 10.1186/s12859-020-03919-2 PMC7730797

[64] Nik-Zainal Serena, Van Loo Peter, Wedge David C, Alexandrov Ludmil B, Greenman Christopher D, Lau King Wai, Raine Keiran, Jones David, Marshall John, Ramakrishna Manasa, The life history of 21 breast cancers. Cell, 149(5):994–1007, 2012. 22608083 10.1016/j.cell.2012.04.023 PMC3428864

