# Inferring cancer type-specific patterns of metastatic spread using Metient

**DOI:** 10.1101/2024.07.09.602790

**Published:** 2025-02-03

**Authors:** Divya Koyyalagunta, Karuna Ganesh, Quaid Morris

**Affiliations:** 1 Tri-Institutional Graduate Program in Computational Biology and Medicine, Weill Cornell Medicine, New York, NY 10065, USA; 2 Computational and Systems Biology Program, Sloan Kettering Institute, New York, NY 10065, USA.; 3 Department of Medicine, Memorial Sloan Kettering Cancer Center, New York, NY, USA; 4 Molecular Pharmacology Program, Sloan Kettering Institute, Memorial Sloan Kettering Cancer Center, New York, NY, USA

**Keywords:** migration history inference, metastasis, combinatorial optimzation

## Abstract

Cancers differ in how they establish metastases. These differences can be studied by reconstructing the metastatic spread of a cancer from sequencing data of multiple tumors. Current methods to do so are limited by computational scalability and rely on technical assumptions that do not reflect current clinical knowledge. Metient overcomes these limitations using gradient-based, multi-objective optimization to generate multiple hypotheses of metastatic spread and rescores these hypotheses using independent data on genetic distance and organotropism. Unlike current methods, Metient can be used with both clinical sequencing data and barcode-based lineage tracing in preclinical models, enhancing its translatability across systems. In a reanalysis of metastasis in 169 patients and 490 tumors, Metient automatically identifies cancer type-specific trends of metastatic dissemination in melanoma, high-risk neuroblastoma, and non-small cell lung cancer. Its reconstructions often align with expert analyses but frequently reveal more plausible migration histories, including those with more metastasis-to-metastasis seeding and higher polyclonal seeding, offering new avenues for targeting metastatic cells. Metient’s findings challenge existing assumptions about metastatic spread, enhance our understanding of cancer type-specific metastasis, and offer insights that inform future clinical treatment strategies of metastasis.

## Introduction

Metastasis is associated with 90% of cancer deaths, yet its pathophysiology remains poorly understood^1^. It remains unclear how often clones seed metastases polyclonally, or how often metastases are capable of seeding other metastases, such as through intermediate lymph nodes^2–10^. It is also not known whether metastatic potential is rare, and thus gained once in the same cancer, or common, and thus gained multiple times^11–14^. The answers to all these questions would improve the understanding and clinical management of metastasis, and could be gleaned from reconstructing migration histories of metastatic clones from clinical sequencing data.

However, this reconstruction requires solving a complex combinatorial optimization problem^2–4^, which previous algorithms have addressed by solving a more tractable, oversimplified maximum parsimony problem^5,15–18^. Early approaches framed migration history reconstruction as a classical phylogenetic problem of labeling interior nodes based on leaf node labels^19–21^, thereby assuming that the optimal reconstruction minimized the number of migrating clones. While these methods scale well to large datasets, such as those generated by lineage tracing experiments^22^, they often yield unrealistic reconstructions, including extensive reseeding between metastatic sites^17^. MACHINA^17^ replaced the simple parsimony model with multi-objective parsimony criteria that also incorporate the number of seeding sites and polyclonal migration events when scoring histories. MACHINA generates more biologically plausible migration histories on small datasets, but its computational complexity makes its unusable on larger datasets, such as those generated by barcode-based single-cell lineage tracing.

Another key limitation is that existing algorithms only return a single migration history, even when multiple equally parsimonious solutions exist. Using multi-objective parsimony scoring methods can also lead to conflicts where different solutions are most parsimonious under different weightings of the parsimony criteria, and currently these conflicts are resolved using pre-defined, ad hoc criteria^15–17^. For example, one common assumption is that metastases can only be seeded from the primary tumor^14^. However, such assumptions are challenged by emerging clinical data suggesting that metastasis-to-metastasis seeding might be more common. Indeed, one prevailing model in oncology, the “sequential progression model” – which posits that lymph node metastases give rise to distant metastases – is the rationale for surgical removal of lymph nodes^23^ and a recent phylogenetic analysis found that the sequential model applied to a third of patients in a colorectal cohort^24^. Pre-deciding primary-only seeding dismisses the possibility of metastasis-to-metastasis seeding before looking at the data. These ad hoc rules used to define parsimony also impact other interpretations of metastatic spread, such as the frequency that sites are seeded by multiple tumor cells. While multiple lineage tracing experiments in animal models have detected that a majority of tumors are seeded polyclonally^25^, human tumor sequencing studies report lower rates of polyclonality^7^, here we show this discrepancy is partly due to previous modeling assumptions. By pre-biasing their reconstructions, current algorithms undermine a key goal in metastasis research: determining which patterns of metastatic spread are prevalent in different cancer types.

To address these issues and overcome the limitations of previous tools (Supplementary Table 1), we introduce Metient (**met**astasis + grad**ient**). Metient is a principled statistical algorithm that proposes multiple potential hypotheses of metastatic spread in a patient. Metient combines modern stochastic optimization techniques with metastasis priors, i.e., new biologically-grounded, migration history scoring criteria. These priors allow Metient to navigate trade-offs among competing explanations, align predictions with empirical data, and uncover trends specific to cancer subtypes. Together, these advances allow Metient to resolve the current challenges in scalability, parsimony resolution, and biological plausibility in migration history reconstruction.

On realistic simulations, Metient outperforms MACHINA in reconstructing the ground truth migration history. When applied to patient cohorts with breast, skin, ovarian, neuroblastoma, and lung cancers, Metient automatically identifies all plausible expert-assigned migration histories and, in some cases, uncovers more biologically plausible reconstructions, especially when previous analyses on this same data pre-selected a favored seeding pattern. In these cohorts, Metient reveals that polyclonal metastatic seeding occurs far more frequently than previously reported, leading to more robust, experimentally-aligned estimates of clonality. Notably, Metient is the first computational framework capable of scaling to lineage tracing datasets comprising thousands of single cells while avoiding the restrictive assumptions of simplified migration history inference methods.

Metient is free, open-source software that includes easy-to-use visualization tools to compare multiple hypotheses of metastatic dissemination. Metient is accessible at https://github.com/morrislab/metient/.

## Results

### The Metient algorithm

**Figure 1.**
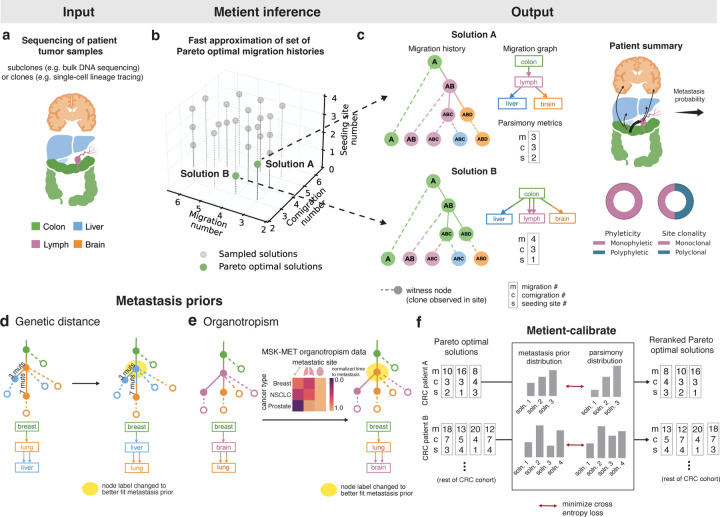
A scalable framework for migration history inference evaluates multiple hypotheses of metastatic spread. (a) Data on clone or subclone frequencies from multiple tissue samples is input into Metient. (b) Metient efficiently recovers multiple Pareto-optimal migration histories based on the three counts: migrations, comigrations and seeding site number. (c) Metient solutions can be represented by a migration history, a tree with (1) an anatomical site labeling of its internal nodes, and (2) leaf nodes (witness nodes) representing a node’s presence in an anatomical site. A migration graph summarizes the migration edges of the migration history. Parsimony metrics indicate the number of migrations $\left(m\right)$, comigrations $\left(c\right)$, and seeding sites $\left(s\right)$. Metient can report summary metrics over the Pareto front, such as the probability of a particular tissue-to-tissue transition, and the percentage of phyleticity and clonality that is inferred. (d) An example of how using genetic distance (a metastasis prior) can promote migration histories with migrations on longer edges with more mutations. The assigned anatomical site label of the highlighted node changes. (e) An example using organotropism (a metastasis prior) to identify migration histories with unlikely metastatic events, such as subsequent metastasis from the brain. The anatomical site label of the highlighted node is changed. (f) Metient-calibrate uses the metastasis priors to fit weights for the parsimony metrics, this calibrated parsimony model can then be used to rescore the Pareto front of migration histories produced for each patient in that cohort.

Migration history inference algorithms aim to reconstruct the spread of cancer clones across anatomical sites using molecular sequencing data from primary and metastatic tumors, paired with an unlabeled tree encoding their genetic ancestry (Figure 1a). These methods estimate the proportions of clonal populations at each site (referred to as "witness nodes", Figure 1b,c), or use observed cell locations directly (e.g., from lineage tracing data). The interior nodes of the tree are then labeled with anatomical sites, defining migration events — tree edges connecting clones assigned to different sites are deemed “migration edges”. The reconstructed history is referred to as a "migration history" (Figure 1c).

The most advanced existing algorithm for migration history inference, MACHINA^17^, scores migration histories using three parsimony metrics: (1) migrations— the number of times a clone is established at a new site^4,15–17^; (2) comigrations—the number of migration events involving multiple clones traveling together; and (3) seeding sites—the number of sites from which clones migrate. MACHINA searches for the most parsimonious history by minimizing these metrics using mixed-integer linear programming (MILP)^26^.

While effective, this approach has critical limitations. First, MILP solvers require hard constraints and linear objectives, preventing the integration of complex, biologically-motivated scoring criteria. Second, they identify only a single optimal solution, even when multiple equally plausible histories exist, leading to biases in reconstructions. Finally, MILP methods scale poorly, making them unsuitable for analyzing large trees, such as those arising from single-cell lineage tracing experiments.

Metient addresses these issues with two key innovations. First, it replaces the MILP framework with stochastic optimization, leveraging low-variance gradient estimators to efficiently search the space of possible migration histories (Supplementary Figure S1, Methods, Supplementary Information). Second, it introduces biologically-informed “metastasis priors” to resolve ambiguities in equally parsimonious solutions and resolve trade-offs among migrations, comigrations, and seeding sites (Figure 1c–e) in scoring parsimony. The first step in Metient’s inference is to define a Pareto front^27^ of potential solutions by searching for parsimonious migration histories under a wide range of “parsimony models” (Supplementary Table 2) represented by a set of weights – $w_{m}$, $w_{c}$, and $w_{s}$ – assigned, respectively, to the number of migrations (indicated by m), comigrations $\left(c\right)$, and seeding sites $\left(s\right)$. A migration history’s parsimony score, p, is the model-weighted average of these three parsimony metrics, i.e., $p=w_{m}m+w_{c}c+w_{s}s$. Different parsimony models favor different histories on the Pareto front.

### Metient-calibrate fits cancer type-specific parsimony models

To assess the importance of considering multiple hypotheses of metastatic spread, we defined four different cancer type-specific patient cohorts consisting of genomic sequencing of matched primary and multiple metastases: melanoma^3^, high-grade serous ovarian cancer (HGSOC)^4^, high-risk neuroblastoma (HR-NB)^9^, and non-small cell lung cancer (NSCLC)^14^. After quality control (Supplementary Information), our dataset included 479 tumors (143 with multi-region sampling) from 167 patients (melanoma: n=7, HGSOC: n=7, HR-NB: n=27, NSCLC: n=126). Among patients with multiple metastases, 38.2% of patients (29/76) had multiple Pareto-optimal migration histories; this frequency increases to 53% for patients with three or more metastases. When it returns a single solution, Metient has ruled out all other sampled solutions as suboptimal, enhancing confidence in the single Pareto-optimal history. Different Pareto-optimal histories often represented different overall patterns of metastatic spread. For example, Figure 1c shows a patient with metastatic colorectal cancer with two Pareto-optimal reconstructions: one in which a lymph node metastasis gives rise to all other metastatic tumors (solution A), and another where most metastases are seeded directly from the primary tumor (solution B). Here, forcing an arbitrary choice between the two reconstructions determines whether one concludes that the lymph node acted as a staging site for metastatic spread.

MACHINA resolves parsimony conflicts by prioritizing the minimization of migrations, comigrations, and seeding sites in a fixed hierarchical manner (i.e., $w_{m}>>w_{c}>>w_{s}$). Other methods simplify further, focusing solely on minimizing migrations^4,15,22^. Such a rigid model can obscure important differences among cancers. For example, in ovarian cancer metastatic events are often polyclonal due to the dissemination of clusters of metastatic cells through peritoneal fluid, these trends would lead to more migrations relative to comigrations^28–30^. In contrast, multiple seeding sites might be more common in the estimated 23.4% of cancer cases have lymph node involvement^31^, particularly in cancer types like non-small cell lung cancer^32^ where this frequently increases up to 87%. It has been proposed that metastatic cells can make a “pit stop” at regional lymph nodes before disseminating to other distant sites^33^. Prescribing a single, cancer-independent parsimony model risks missing these clinically-relevant differences.

In contrast to an ad hoc approach, Metient uses metastasis priors to both define a cancer type-specific parsimony model and to resolve ties between histories with equal parsimony metrics. Metient, in calibrate mode, fits a patient cohort-specific parsimony model using the metastasis priors (Figure 1d–f; Methods). This calibrated model is used to rank Pareto-optimal histories that differ in their parsimony metrics. Metient also provides a pan-cancer parsimony model, calibrated to all four cohorts combined, for use when an appropriate patient cohort is not available. This pan-cancer model is a first step toward estimating the likelihoods of key events in metastatic dissemination across cancers, providing a generalized framework that can be applied to any dataset.

Metient provides two metastasis priors. One, genetic distance, can be applied to any cohort. The other, organotropism, can be used when appropriate tissue-type information are available for the sequenced tumor samples. The genetic distance prior considers the genetic distance of migration edges in the labeled clone tree; here genetic distance is defined as the number of mutations gained in the child clone and not present in the parent clone. This prior favors histories with higher averaged genetic distances on migration edges (Figure 1d, Methods, Supplementary Information). Theoretical and empirical evidence supports this approach. Colonizing clones at metastatic sites typically undergo clonal expansions^34^, making their mutations more detectable than those in the source tumor, where private mutations often remain undetected due to insufficient clonal expansion. Furthermore, metastasizing cells face strong selection pressures, which are associated with elevated mutation rates^35–37^. These pressures, combined with the increased genomic instability observed in metastases^35–37^, result in higher tumor mutation burdens compared to primary tumors^31,38,39^. Analysis of our cohorts confirm this trend, with migration edges showing higher genetic distances than non-migration edges when the most parsimonious migration history was unambiguous (Supplementary Figure S2).

The second metastasis prior, organotropism, is derived from data from over 25,000 metastatic cancer patients to compute the observed preference of certain cancer types for specific metastatic sites^40^. All else being equal, this prior favors more common migration patterns, like migration from a breast-seeded lung metastasis to the brain, over rarer ones, like a breast-seeded brain metastasis to the lung^41^ (Figure 1e). The organotropism prior does not directly model secondary metastasis-to-metastasis migrations, instead it indirectly captures these patterns through its scoring of primary-to-metastasis events (Methods).

Metient uses these priors to complement, rather than replace, the parsimony model. In our benchmarking analyses on simulated data, we find that using genetic distance alone to score migration histories performs poorly and can result in the inference of highly non-parsimonious migration histories (Supplementary Tables 3, 4, see also PathFinder^18^). Therefore, genetic distance and organotropism are used to rank solutions only after defining the Pareto front. Metient reports and visualizes all Pareto-optimal solutions it finds, allowing users to evaluate multiple plausible hypotheses and select the one most consistent with biological and clinical insights.

### Simulated data validates the genetic distance prior and shows that Metient is state-of-the-art

To assess Metient’s new objective and gradient-based optimization on data with a provided ground-truth, we ran benchmarking analyses along with the state-of-the-art migration history inference method (MACHINA^17^) on simulated data for 80 patients with 5–11 tumor sites and various patterns of metastatic spread. These data were originally used to validate MACHINA.

First, to assess the added value of the genetic distance prior, we used Metient-calibrate to fit a calibrated parsimony model, and compared calibrated Metient with a version of Metient that used the parsimony model implied by MACHINA. We fit two calibrated models, one on a cohort with primary-only seeding and another on a cohort with metastasis-to-metastasis seeding. Metient-calibrate improved recovery of the ground truth migration graph (Supplementary Figure S3a) over the fixed parsimony model (Calibrate vs. Evaluate (MP) in Supplementary Table 3), showcasing the ability of the metastasis priors to learn metastatic patterns specific to a cohort and improve overall accuracy. In addition, Metient-calibrate predicts ground truth seeding clones and migrations graphs at least as accurately as MACHINA, with overall improvements as tree sizes get larger (Supplementary Figure S3b) and significant improvements in inferring the seeding clones for patients with more complex metastasis-to-metastasis seeding (Supplementary Figure S3b right; p=0.0021).

Notably, even though the Metient framework is non-deterministic, it identifies the same top solution 97% of the time across multiple runs (Supplementary Figure S3c). In addition to its improved accuracy, Metient runs up to 55x faster (3.95s with Metient-64 vs. 221.19s with MACHINA for a cancer tree with 18 clones and 9 tumors) on this benchmark, showcasing our framework’s scalability as tree sizes grow (Supplementary Figure S3d).

### Multi-cancer analysis of clonality, phyleticity, and dissemination patterns

**Figure 2.**
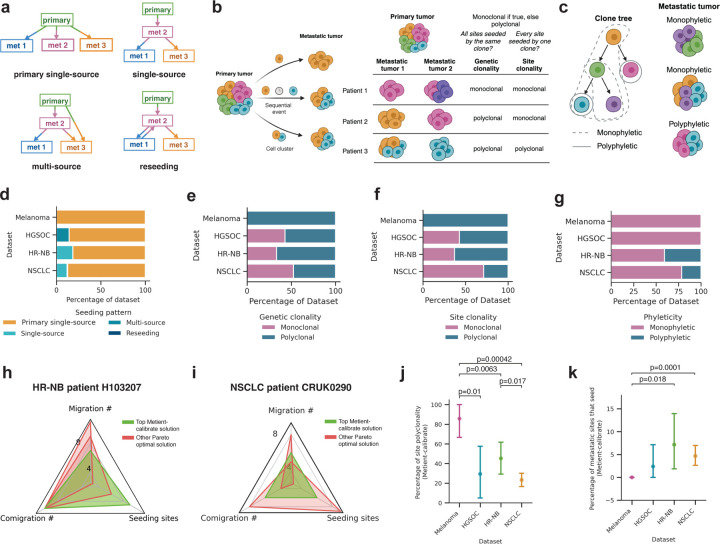
Multi-cancer analysis of clonality, phyleticity, and dissemination patterns. **(a)** Schematic illustrates four metastatic seeding patterns. met: metastasis. **(b)** Schematic illustrates metastases seeding by one or multiple clones either sequentially or in a cell cluster, table defines genetic clonality versus site clonality. Colors represent genetically distinct cancer cell populations. **(c)** Schematic illustrates the definitions of monophyletic and polyphletic seeding. Monophyletic indicates that the colonizing clone closest to the root can reach every other colonizing clone on the clone tree. Colors represent genetically distinct cancer cell populations. Distribution of **(d)** seeding patterns, **(e)** genetic clonality, **(f)** site clonality and **(g)** phyleticity for each dataset, as inferred by Metient’s top migration history. **(h)** Radar plot showing the unique Pareto-optimal metrics for migration histories inferred by Metient for HR-NB patient H103207. **(i)** Radar plot showing the unique Pareto-optimal metrics for migration histories inferred by Metient for NSCLC patient CRUK290. **(j)** Comparing across datasets the percent of migrations that are polyclonal for the top Metient solution. Statistical significance assessed by a Welch’s t-test. Error bars are the standard error for each dataset. **(k)** Comparing across datasets the percent of metastatic sites that seed for the top Metient solution. Statistical significance assessed by a Welch’s t-test. Error bars are the standard error for each dataset.

After validating calibrated Metient’s ability to recover ground-truth metastatic patterns in simulated data, we applied it to the patient cohorts with melanoma, HGSOC, HR-NB, and NSCLC to explore shared and unique patterns of metastatic spread. Due to missing or inadequate anatomical site labels, we only calibrated to genetic distance. We examined three aspects of metastatic dissemination: seeding pattern (single-source from the primary or another site, multi-source, reseeding; Figure 2a), clonality (number of clones seeding metastases; Figure 2b), and phyleticity (metastatic potential is gained in one or multiple evolutionary trajectories; Figure 2c; Methods). We distinguish between genetic polyclonality (multiple clones seeding metastases) and site polyclonality (multiple clones seeding individual sites), to highlight cases where each metastasis is seeded by a single clone, but different sites may be seeded by different clones, in order to distinguish cases where different site-specific mutations are needed for metastasis (Figure 2b).

Consistent with expert annotations^3,4,9,14,17^, Metient finds that single-source seeding from the primary tumor is the most common pattern in every cohort (Figure 2d). However, Metient identifies a larger fraction of polyclonal migration patterns than previous reports^8,14^: 53.3% of patients show genetic polyclonality (Figure 2e), and 38.3% of patients have site polyclonality (Figure 2f). Overall, Metient estimates that 34.1% of sites (107/314) are seeded by multiple clones; nearly double previous estimates of site polyclonality (19.2%) based on an analysis of breast, colorectal and lung cancer patients^8^. Notably, the choice of parsimony model affects polyclonality, as reducing the number of seeding sites increases polyclonal migrations (Supplementary Figure S4a). However, Metient’s findings do not stem from assuming primary-only seeding, as done in prior work.

Metient’s phyleticity estimates are similar to previous reports: 77.2% of patients (129/167) have a monophyletic tree where metastatic potential is gained once and maintained (Figure 2g). For some patients, this is due to the root clone being observed in one or more metastatic sites (Supplementary Figure S4b), and for other patients, all colonizing clones belong to a single subtree of the clone tree whose root is itself a colonizing clone. Either scenario suggests that metastatic potential is less likely to be gained via multiple, independent evolutionary trajectories across cancers.

### Cancer type-specific metastasis trends

We next examined cancer type-specific differences in metastatic trends, first using a bootstrapping approach to ensure that the parsimony metric weights were reproducible and reflective of population level patterns for a particular cancer type. We fit parsimony metric weights to 100 bootstrapped samples of patients within the cohort (Methods), and found that 98.4% of patients ranked the same top solution across bootstrap samples, indicating that Metient can learn a reproducible cancer type-specific model for the melanoma and HGSOC cohorts which have only seven patients each.

These cancer type-specific parsimony metric weights lead to cohort-specific choices on how Metient ranks a patient’s Pareto front of migration histories. For example, Metient chooses the solution on the Pareto front with lowest migration number (i.e. colonizing clones) for HR-NB patient H103207 (Figure 2h), but the solution with the median value of each metric for NSCLC patient CRUK0290 (Figure 2i). To systematically assess the impact of cohort-specific rankings we computed the percentage of polyclonality and number of seeding sites in the top ranked solution for patients with each cancer type. Overall, we found a significantly higher fraction of polyclonal migrations in melanoma than HGSOC, HR-NB and NSCLC patients (Figure 2j). One explanation for this heightened polyclonality in melanoma patients is that all patients in the cohort had locoregional skin metastases, a common “in-transit” metastatic site around the primary melanoma or between the primary melanoma and regional lymph nodes. These locoregional sites could have multiple cancer cells traveling together through hematogeneous or lymphatic routes to seed new localized tumors^42^. The HR-NB and NSCLC cohorts had significantly higher percentages of metastasis-to-metastasis seeding than melanoma (Figure 2k). As described below, in the HR-NB cohort, multiple patients exhibit metastasis-to-metastasis seeding within an organ or between commonly metastatic sites. In the NSCLC cohort, 76.2% of patients have lymph node metastases, from which it is known that further metastases are commonly seeded^43^. Indeed, Metient predicted that 75% (12/16) of NSCLC patients with metastasis-to-metastasis seeding had seeding from a lymph node to other metastases, suggesting that lymph nodes serve as a common intermediate staging site.

### Metastasis priors identify biologically relevant migration histories and alternative explanations of spread

A core advance of Metient is its ability to identify and rank the Pareto-optimal histories of a patient’s cancer. To assess how well our top ranked solution aligns with the most biologically plausible explanation, we compared our inferred migration histories to previously reported, expert-annotated seeding patterns. Of the 167 patients analyzed, 152 patients had an expert or model-derived annotation available. In 84% of cases (128/152), Metient’s predictions aligned with previously reported site-to-site migrations. For the remaining 24 cases, Metient either identified a more parsimonious history or included the expert annotation on the Pareto front but prioritized a different solution based on its metastasis priors. We provide a detailed case-by-case comparison in the Supplementary Information and Supplementary Figures S5, S6, S7, S8, and highlight two interesting HR-NB cases below.

**Figure 3.**
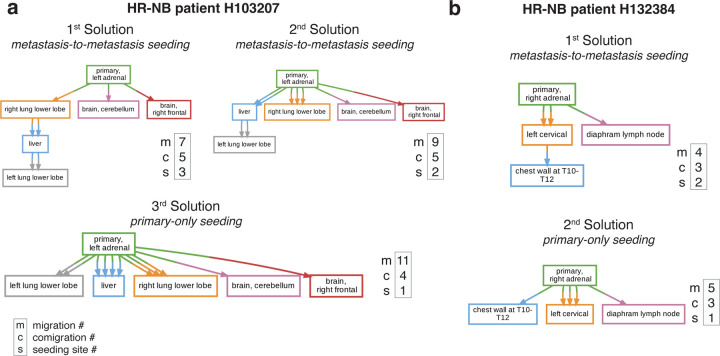
Metient finds biologically relevant migration histories. **(a)** All ranked Pareto-optimal migration graphs inferred by Metient-calibrate for HR-NB patient H103207. **(b)** All ranked Pareto-optimal migration graphs inferred by Metient-calibrate for HR-NB patient H132384.

Metient predicts metastasis-to-metastasis seeding for two HR-NB cases (H103207, H132384), differing from earlier reports of direct seeding from the primary^9^. HR-NB patient H103207 exhibits evidence for two potential metastasis-to-metastasis seeding scenarios, both involving seeding between the lung and liver (Figure 3a). While the exact prevalence of metastasis-to-metastasis seeding between the liver and lung in HR-NB is unknown, both are common sites of metastases across cancer types due to cancer cells’ ability to take advantage of rich blood supply and vascular organization^40^. Colonization of the lung by clones from a primary liver tumor is common^40,44,45^ and, similarly, the liver is a common site of metastasis for primary lung cancer patients^40,46^, suggesting that transitions from a liver-competent cancer clone to a lung-competent one or vice versa could be common as well. The lung- and liver-colonizing clones emerge on a shared branch, separate from the branch that gives rise to the CNS-colonizing clones (Supplementary Figure S5a). This suggests that evolution within the primary tumor generated clones with shared metastatic potential for the lung and liver that was separate from the metastatic potential of CNS-colonizing clones. This is consistent with the clonal analysis reported by Gundem et al.^9^, and further supports the proposed metastasis-to-metastasis seeding. Patient H132384 also shows evidence of metastasis-to-metastasis seeding, but from bone-to-bone, first to the left cervical and secondarily to the chest wall (Figure 3b). Metastasizing cells exhibit organ-specific genetic and phenotypic changes to survive in a new microenvironment^40^, suggesting that seeding an additional tumor within the same organ microenvironment is more likely than a secondary migration from the primary adrenal tumor in this case. In addition, prior experimental evidence shows that bone metastases prime and reprogram cells to form further secondary metastases^47,48^. These posited metastasis-to-metastasis seedings are thus supported by site proximity or organotropism, or both, and these Metient reconstructions were made without providing such information.

**Figure 4.**
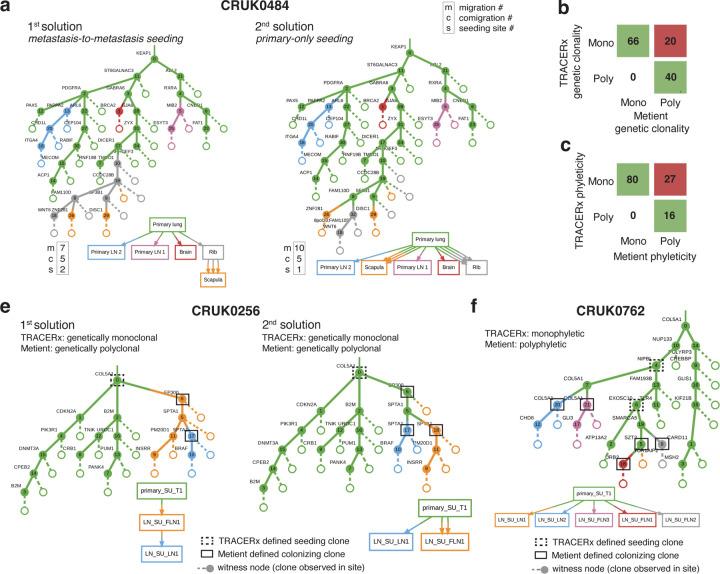
Metient identifies more polyclonality and polyphyelticity in a large-scale NSCLC cohort. **(a)** The top two Pareto-optimal solutions for NSCLC patient CRUK0484 as ranked by Metient-calibrate. Comparison of Metient’s inference to TRACERx’s: **(b)** clonality and **(c)** phyleticity classification. Counts indicate the number of patients in agreement or disagreement. **(d)** All Pareto-optimal solutions for NSCLC patient CRUK0762 as ranked by Metient-calibrate. **(f)** Patient CRUK0762 where seeding pattern and clonality are in agreement between TRACERx and Metient-calibrate but phyleticity differs due to which clones are classified as seeding.

Next, we compared the inferred migration histories from the NSCLC samples we analyzed to an in-depth analysis of the same samples by the TRACERx consortium^14^ which enforces a primary single-source dissemination model for its analysis of clonality and phyleticity. While Metient generally agrees with this dissemination model, Metient predicts metastasis-to-metastasis seeding for several (12.8%; 16/126) patients. For instance, Metient suggests that, in patient CRUK0484, a rib metastasis seeded the scapula (Figure 4a), a pattern consistent with other bone-to-bone metastasis cases like HR-NB patient H132384.

Metient’s highest-scoring solution agrees with TRACERx classifications 84.1% (106/126) for clonality (Figure 4b)) and 78% (96/123) for phyleticity (Figure 4c). Discrepancies arise because TRACERx defines seeding clones as the most recent shared clone between primary and metastasis, while Metient considers the full migration history and accounts for metastasis-to-metastasis seeding, rather than assuming primary-only seeding. Consequently, Metient has higher sensitivity for detecting colonizing clones, leading to increased detection of polyclonal and polyphyletic events.

In 20 NSCLC patients, Metient inferred that multiple colonizing clones are needed to explain the full migration history, whereas no history is consistent with the TRACERx identified colonizing clones. For example, in CRUK0256 (Figure 4d), Metient identifies multiple colonizing clones, in either the metastasis-to-metastasis seeding scenario (Figure 4d first solution) or a primary-only seeding scenario (Figure 4d second solution), whereas TRACERx identifies only the root clone. Similarly, TRACERx’s phyleticity inferences, constrained by its definition of colonizing clones, miss some potential polyphyletic events, misclassifying them as monophyletic. For instance, Metient classifies 27 cases as polyphyletic where TRACERx classifies them as monophyletic, as in patient CRUK0762 (Figure 4e). While monophyleticity remains the dominant pattern in NSCLC (65%), we suggest that polyphyleticity may be underrecognized due to TRACERx’s lower sensitivity for detecting colonizing clones.

### Metient identifies the mediastinum as an early hub for metastatic dissemination in lung adenocarcinoma single-cell lineage tracing experiments

Recent advancements in Cas9-based molecular recording technologies have enabled precise mapping of cellular evolution and metastatic progression in cancer, offering invaluable insights for metastasis research. However, existing multi-objective models fail to scale to the size of this data, which often profiles thousands of single cells, while simplified approaches frequently yield erroneous results. We demonstrate the application of Metient to lineage tracing data from a human *KRAS*-mutant lung adenocarcinoma cell line, tracking 100 clones in a mouse xenograft model^22^. Metient is the first method capable of analyzing such large datasets—encompassing clones with up to 15,488 cells—without relying on oversimplified models of metastasis.

**Figure 5.**
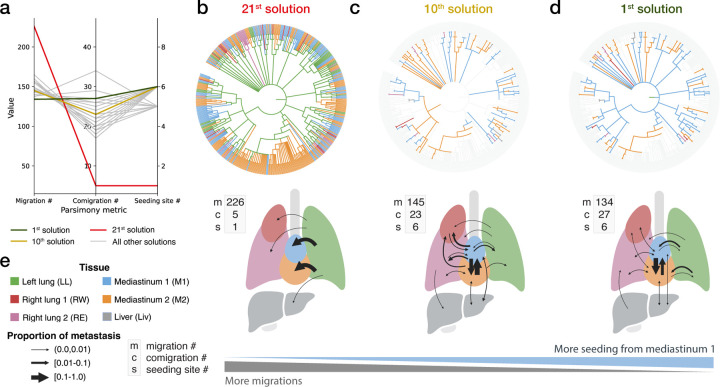
Clones from lineage tracing data have large Pareto fronts with many different possible patterns of spread. (a) The parsimony metrics for all 21 solutions on the Pareto front for clone 15. (b-d) Solutions by their rank on the Pareto front, where more top ranked solutions exhibit lower migration numbers and more seeding via the mediastinum 1. Solutions ranked 1 and 10 only show colored migration edges that are different from the primary-only seeding solution ranked 21. Migration edges that are the same as those in solution ranked 21 are in light grey. (e) Legend for colors and width of arrows used in b-d.

Using Metient’s pan-cancer parsimony model, calibrated on bulk-sequencing data, we identified diverse patterns on the Pareto front, providing insights into the different possible trajectories of metastatic spread for each clone. For example, clone 15’s Pareto front consisted of 21 unique solutions (Figure 5a). The lowest-ranked solution (Figure 5b) exhibits primary-only seeeding, whereas higher-ranked solutions revealed greater involvement of a mediastinum seeding site, thereby reducing the overall number of migration events (Figure 5c,d). When multiple solutions on the Pareto front had equal parsimony metrics, Metient used the organotropism prior to distinguish between them (see Supplementary Information for details).

**Figure 6.**
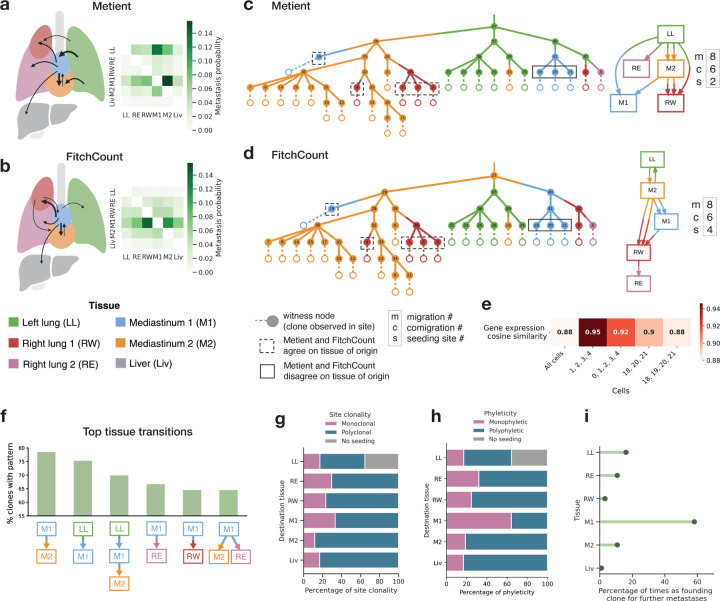
Metient’s migration histories of lineage tracing clones differ from simplified models and implicate the mediastinum as an early metastatic hub. Probability of metastasis from source to destination tissue, aggregated over all 100 clones, as inferred by Metient in (a) and FitchCount in (b). Width of arrows on the body maps are scaled to the frequency of metastasis between sites, as shown in the heat map. Tissue transition probabilities < 0.05 are omitted. (c) Clone 99’s top ranked migration history by Metient. (d) A random minimum migration solution returned by the Fitch-Hartigan algorithm used by FitchCount for clone 99. (e) The mean cosine similarity of gene expression of cells in clone 99. (f) The top six most frequent subgraphs of migration graphs among the 100 clones. (g) The site clonality of seeding to each destination tissue across all 100 clones. (h) The phyleticity of seeding to each destination tissue across all 100 clones. (i) The percentage of times the first colonizing clone is detected in each tissue.

We compared Metient’s migration history inferences for the 100 clones to those inferred by FitchCount^22^, the method used in the original paper. FitchCount, an extension of the single-objective Fitch-Hartigan algorithm^20,21^, computes tissue transition probabilities for minimum migration number solutions. However, FitchCount neither recovers complete migration histories nor integrates the more biologically relevant multi-objective parsimony model. Its simplified assumptions led to notable differences in the overall tissue transition probabilities. Specifically, Metient postulates that seeding from the primary left lung tumor predominantly spreads to the mediastinal tissues, which function as a hub for further metastatic dissemination (Figure 6a,b).

In addition, there are some notable differences in Metient’s and FitchCount’s migration histories. For example, both methods predict a solution with the same number of metastatic migrations for clone 99, but failing to account for comigrations and seeding sites leads FitchCount to propose a more complex reseeding pattern (Figure 6c,d). This example makes clear that minimum migration solutions are not always the simplest or most biologically relevant explanations of the data. To further evaluate the validity of Metient’s migration histories, particularly where Metient and FitchCount diverged, we used the single-cell RNA-sequencing readouts from the lineage tracing data. Since cells alter their gene expression to adapt to new tissue environments^40,49^, we hypothesized that a cell’s gene expression profile should be more similar to cells that underwent the same metastatic transition—i.e., those originating from the same source tissue and metastasizing to the same final tissue—than to other cells within the same clone. Both Metient’s and FitchCount’s inferred migration histories have significantly increased gene expression cosine similarity for cells that underwent the same metastatic transition (Supplementary Figure S9), with Metient having a slightly higher effect size when the tissue of origin differed from FitchCount (Cohen’s *d* for paired samples: 0.54 for Metient and 0.52 for FitchCount). For example, in clone 99, both methods predict that cells 1–4 were seeded from M2 to RW (Figure 6c,d). As expected, these cells show high gene expression similarity to one another, while the inclusion of cell 0, also in RW but likely originating from a different source, reduces this similarity (Figure 6e). In the same clone, the average gene expression similarity of the group of co-migrating clones (18,20,21) decreases when 19 is added to the group, suggesting that 19 has a different source tissue than clones (18,20,21), as predicted by Metient but not FitchCount (Figure 6c–e).

Metient reconstructs complete migration trees for all Pareto-optimal solutions it finds, allowing for a detailed analysis of migration event sequencing, clonality, and phyleticity. This analysis cannot be done with FitchCount, because it does not return all migration histories it finds, but rather uses a dynamic programming approach that returns one minimum migration number solution along with aggregated counts of tissue transitions over all solutions. In Metient’s reconstructions, mediastinum 1 is the most common source of further dissemination, serving as a hub for systemic spread more often than the primary tumor (Figure 6f). This is consistent with the mediastinal lymph tissue’s anatomical and lymphatic connectivity to the primary lung tumor, as it provides a direct route for cancer cells to metastasize early^50,51^. The original study also detects early colonization of mediastinal lymph tissue using bulk live imaging^22^.

Metient revealed widespread polyclonality and polyphyleticity across this dataset, in line with the aggressive metastatic nature of this cell line^22^ (Figure 6g,h). In a majority of clones, the mediastinum 1 contains the founding clone for further metastases (Figure 6i). This suggests that while subsequent systemic spread is highly polyclonal, the initial seeding of the mediastinum may be driven by a single dominant clone for many cases, suggesting a pivotal role in the early stages of metastatic progression.

## Discussion

We have presented and validated Metient, a novel framework for reconstructing the migration histories of metastasis. In contrast to prior work, Metient defines a Pareto front of possible migration histories, and then uses biologically-motivated metastasis priors to resolve parsimony conflicts in a data-dependent manner. Metient is able to scale to large problems because it adapts Gumbel straight-through stochastic gradient estimation to optimize the combinatorial problem required for history reconstruction. Collectively, these advances improve performance on simulated data, improve biological interpretation on real data, and can define a Pareto front in a fraction of the time that MACHINA, the current state-of-the-art, takes to output a single solution.

We demonstrate that previous algorithms using pre-specified parsimony models and ad hoc rules introduced systematic biases into studies of metastatic spread. For example, a prior analysis of a large NSCLC cohort^14^ assumed primary-only seeding when assessing clonality and phyleticity. This approach excluded plausible histories involving metastasis-to-metastasis seeding, even when MACHINA was used to identify such events. In contrast, Metient reveals that 12.8% of the NSCLC cohort exhibits evidence of metastasis-to-metastasis seeding, predominantly through lymph nodes. By identifying these overlooked metastatic events and using the full migration history to identify colonizing clones, Metient also uncovers higher levels of polyclonality and polyphyleticity in this cohort than previously reported. Furthermore, across all datasets analyzed, Metient consistently reveals almost twice the level of polyclonality previously reported, suggesting that multiple clones more frequently contribute to metastatic progression. This view of metastatic dynamics implies that therapeutic strategies could shift focus from targeting individual cancer cells to disrupting the formation and migration of tumor cell clusters, potentially offering more effective approaches to prevent or reduce metastasis^52^. Metient’s statistical, data-driven approach uncovers unrecognized metastatic patterns and provides an unbiased framework for identifying cancer-type-specific trends, thereby addressing a longstanding challenge in metastasis research.

To demonstrate the versatility and scalability of Metient, we applied it to a *KRAS*-mutant lung adenocarcinoma cell line with single-cell lineage tracing, a domain where migration history reconstruction was previously constrained by only simplified models of metastatic spread. In these data, Metient identified the mediastinum as a key hub for further metastatic dissemination after initial seeding from the lung primary, revealing the particular ordering of seeding that a previous simplified model was not able to infer. Unlike prior multi-objective approaches built on integer linear programming, Metient leverages gradient-based optimization that can efficiently utilize GPUs, enabling scalability to larger and more complex datasets. This capability opens new avenues for studying metastasis at single-cell resolution, offering a powerful tool for future research.

Currently, Metient uses genetic distance and organotropism as its metastasis priors, as well as MACHINA’s parsimony criteria, however, the Metient framework is designed to be easily extensible. New parsimony criteria can be added as long as they can be computed as matrix functions of the tree adjacency matrix and vertex labeling matrices. Adding a new prior simply requires writing a scoring function because Metient incorporates auto-differentiation to compute its gradient updates. For instance, the framework could be easily extended to incorporate mutational signatures as a prior, since metastases exhibit shifts in mutational signature composition^53,54^. Metient has some limitations. It scales well in compute time for larger clone trees or more samples but, because the loss landscape complexity increases substantially, in approximately 1% of cases in bulk-sequencing data and 5% of single-cell lineage tracing data, Metient became stuck in local minima. This problem was resolved when we ran Metient multiple times and with more samples, and we recommend this practice with larger reconstruction problems. One criteria to assess convergence is when the Pareto front remains unchanged. Other migration history algorithms are also highly sensitive to the complexity of the loss landscape, and convergence issues that they face are not necessarily resolved by rerunning the algorithm. Also, Metient is not designed to consider subclonal copy number alternations (CNAs) when correcting its estimated variant allele frequencies for CNAs. Using the descendant cell fraction (DCF)^55^ or phylogenetic cancer cell fraction (phyloCCF)^56^ as inputs to Metient could solve this. Alternatively, one could input which clones are in which samples directly into Metient instead of the allele frequencies that define subclones, as we did to run Metient on the lineage tracing data. Finally, we note that choice of clustering and tree inference algorithm used when inputting data into Metient can impact both the clonality and phyleticity classifications. In an attempt to most accurately compare our migration histories to previously reported results, where possible, we used the same clustering and trees inferred for the original datasets.

In conclusion, we show that Metient offers a fast and adaptable, fully automated framework that leverages molecular tumor sequencing data to probe enduring questions in metastasis research.

## Methods

### Estimating observed clone proportions

The first step of Metient is to estimate the binary presence or absence of clone tree $\left(T\right)$ nodes in each site. The clone tree T can either be provided as input, or inferred from the DNA sequencing data using, e.g., Orchard^57^, PairTree^58^, SPRUCE^59^, CITUP^60^, or EXACT^61^. Building on a previous approach as described by Wintersinger et al.^58^, Metient estimates the proportion of clones in each site using the input clone tree T and read count data from bulk DNA sequencing. For a genomic locus j in anatomical site k, the probability of observing read count data $x_{kj}$ is defined using the following:

- $A_{kj}$ is the number of reads that map to genomic locus j in anatomical site k with the variant allele
- $R_{kj}$ is the number of reads that map to genomic locus j in anatomical site k with the reference allele
- $\omega_{kj}$ is a conversion factor from mutation cellular frequency to variant allele frequency (VAF) for genomic locus j in anatomical site k

Using a binomial model, we then estimate the proportion of anatomical site k containing clone c using $p\left(x_{kj}\left|F_{kj}\right.\right)=\text{Binom}\left(A_{kj}\left|A_{kj}+R_{kj},\omega_{kj}\;F_{kj}\right.\right)$. Where F=UB is the mutation cellular frequency matrix, $B\in {\left\{0,1\right\}}^{C\times M}$ is 1:1 with a clone tree, where C is the number of clones and M is the number of mutations or mutation clusters, and $B_{cm}=1$ if clone c contains mutation m (Figure S1b). $U\in {\left[0,1\right]}^{K\times C}$, where K is the number of anatomical sites, and $U_{kc}$ is the fraction of anatomical site k made up by clone c (Figure S1b). An L1 regularization is used to promote sparsity, since we expect most values in U to be zero. For details on how to set $\omega_{kj}$, see “Variant read probability calculation $\left(\omega \right)$ ” in Supplementary Information. An alternative way to find a point estimate of U is using a previously described projection algorithm for this problem^57,58,61,62^. A point estimate U can be found by optimizing the following quadratic approximation to the binomial likelihood of U given B and F:

(1) $$LP\left(U\left|B,F,W\right.\right)=mn_{\hat{F},U}{\left\|W⊙\left(F-\hat{F}\right)\right\|}^{2}\;\;s.t.\;\;U1\leq 1,U\geq 0,\hat{F}=UB$$

where $\left\|\cdot \right\|$ is the Frobenius norm, **1** is a vector of 1s, F are the observed mutation frequencies, W is a K×M matrix of inverse-variances for each mutation in each sample derived from F, and ⊙ is the Hadamard, i.e., element-wise product. The definition for W is as described in previous work^58,61^.

We use U (estimated in either of the previously described ways) to determine if a clone c is present in an anatomical site k. If c is present, we attach a witness node with label k (leaf nodes connected by dashed lines in Figure S1b, c) to clone c in clone tree T. We deem c to be present in k if $U_{kc}>5\%$ for a given anatomical site k and clone c. If a clone c does not make up 5% of any of the K anatomical sites, and c is a leaf node of the clone tree T, we remove this node since it is not well estimated by the data.

Here the term “anatomical site” is used to describe a distinct tumor mass. If multiple samples are taken from the same tumor mass, we combine them as described in “Bulk DNA sequencing pre-processing: Non-small Cell Lung Cancer Dataset”.

Note that read count data are only used to determine which clones are present in which sites, if a matrix indicating the presence or absence of each clone in each anatomical site is available, it can be used as an input to replace the read count data. These clone-to-site assignment matrices can be derived, e.g., from single-cell data.

### Labeling the clone tree

The next step in inferring a migration history is to jointly infer a labeling of the clone tree and resolve polytomies, i.e., nodes with more than two children. Polytomy resolution is discussed in the section “Resolving polytomies”. Because we are interested in identifying multiple hypotheses of metastatic spread, Metient seeks to find multiple possible labelings of a clone tree T. Each possible labeling is represented by a matrix $V\in {\left\{0,1\right\}}^{K\times C}$, where K is the number of anatomical sites and C is the number of clones, and $V_{kc}=1$ if clone c is first detected in anatomical site k. Each column of V is a one-hot vector. We solve for an individual V by optimizing the evidence lower bound, or ELBO, as defined by:

(2) $$\text{ELBO}\left(q\right)=E_{q\left(V\right)}\left[\log p\left(U,T,V\right)\right]+ℍ\left(V\right)$$

Where $E_{q\left(V\right)}\left[\log p\left(U,T,V\right)\right]$ evaluates a labeling based on parsimony, genetic distance, and organotropism, and the second term is the entropy term. U has been optimized as described in the previous section “Estimating observed clone proportions”, or taken as input from the user. See Supplementary Information for a full derivation of this objective. Because V is a matrix of discrete categorical variables, we do not optimize V directly, but rather the underlying probabilites of each category that we optimize using a Gumbel-softmax estimator (see “Gumbel-softmax optimization”).

### Gumbel-softmax optimization

In the previous section, we described how to score the matrix representation of the labeled clone tree, V. Here, we describe how to optimize V via the straight-through estimator of the Gumbel-Softmax distribution^63,64^. Starting with a matrix $\psi \in {\left\{0,1\right\}}^{K\times C}$, of randomly initialized values, where K is the number of anatomical sites and C is the number of clones, and each column represents the unnormalized log probabilities of clone c being labeled in site k:

1. At every iteration, for each clone c, we sample ${\text{g}}_{1c}\ldots {\text{g}}_{kc}$, k i.i.d. samples from Gumbel(0,1) and compute $y_{ic}=\psi_{ic}+{\text{g}}_{ic}$.
2. We then sample from the categorical distribution represented by the column vector $\psi_{:c}$ by setting $i^{*}={\text{argmax}}_{i}\;y_{ic}$ and represent that sample with a one-hot encoding in V, i.e., $V_{ic}=1$ if $i=i^{*}$, 0 otherwise.
3. Then we evaluate the $\text{ELBO}\left(\nu \right)$ where
$$\nu_{ic}=\frac{\exp\left(y_{ic}/\tau \right)}{\sum_{j=1}^{k}\exp\left(y_{jc}/\tau \right)}\;\;\;\;\;\;\;\;\text{for}\;\;i=1,\ldots ,k,$$
  using a stochastic approximation based on V, and take the gradient of this ELBO in the backward pass, thus implementing the straight-through estimator.
4. During training, start with a high τ to permit exploration, then gradually anneal τ to a small but non-zero value so that the Gumbel-Softmax distribution, ν resembles a one-hot vector.

At the end of training, as τ approaches 0, then the gradient becomes unbiased and ν approaches V. In order to capture multiple modes of the posterior distribution, each representing different hypotheses about the migration history, we optimize multiple Vs in parallel. To do this, we set up steps 1–3 such that xψs are solved for in parallel^65^ (with a different random initialization for each parallel process), where x is equal to the sample size and is calculated according to the size of the inputs $\left(\propto K^{C}\right)$. See Supplementary Information for further explanation.

### Resolving polytomies

An overview of the algorithm to resolve polytomies is given in Supplementary Figure S11a and b.

1. If a node i in T has more than 2 children, we create r new “resolver” nodes where r is the number of children of node i/2. These new resolver nodes’ vertex labels are jointly inferred along with the refinement of T. The genetic distance between the parent node i and its new resolver node is set to 0 since there are no observed mutations between the two nodes.
2. We allow the children of i to stay as a child of i, or become a child of one of the resolver nodes of i.
3. Any resolver nodes that are unused (i.e. have no children) or which do not improve the migration history (i.e. the parsimony metrics without the resolver node are the same or worse) are removed.

### Fixing optimal subtrees

To improve convergence, we perform two rounds of optimization when solving for a labeled clone tree and resolving polytomies:

1. Solve for labeled trees and resolve polytomies jointly (as described in previous sections).
2. For each pair of labeled tree and polytomy resovled tree, find optimal subtrees. I.e., find the largest subtrees, as defined by the most number of nodes, where all labels for all nodes are equal. This means that there is no other possible optimal labeling for this subtree (there are 0 migrations, 0 comigrations, 0 seeding sites), and we can keep it fixed. Fix these nodes’ labelings and adjacency matrix connections (if using polytomy resolution).
3. Repeat step 1 for any nodes that have not been fixed in step 2.

### Guaranteeing minimum migration solutions on the Pareto front

To guarantee a minimum migration solution on the Pareto front, when polytomy resolution is not being used, we run the Fitch-Hartigan algorithm^20,21^. This aids in output stability when input sizes are very large, however Metient recovers these solutions using its sampling approach (along with multiple minimum migration solutions) in almost all cases.

### Metient-calibrate

In Metient-calibrate, we aim to fit a patient cohort-specific parsimony model using the metastasis priors. To score a migration history using genetic distance, we use the following equation: $\sum_{ij}-lo\text{g}\left({\text{D}}_{\text{ij}}\right){\text{K}}_{\text{ij}}$, where D contains the normalized number of mutations between clones, and K=1 if clone i is the parent of clone j and clone i and clone j have different anatomical site labels.

To score a migration history using organotropism, we use the following equation: $\sum_{i=1}^{K}-lo\text{g}\left(o_{i}\right)\;{\text{g}}_{i}$, where vector o contains the frequency at which the primary seeds other anatomical sites, and vector g contains the number of migrations from the primary site to all other anatomical sites for a particular migration history.

To optimize the parsimony metric weights, Metient identifies a Pareto front of labeled trees for each patient and scores these trees based on (1) the weighted parsimony metrics and (2) the metastasis priors: genetic distance and, if appropriate anatomical labels are available, organotropism. These form the parsimony distribution and metastasis prior distribution, respectively. We initialize with equal weights and use gradient descent to minimize the cross entropy loss between the parsimony distribution and metastasis prior distribution for all patients in the cohort. Once the optimization converges, Metient rescores the trees on the Pareto front using the fitted weights, to identify the maximum calibrated parsimony solution, and genetic distance and organotropism are used to break ties between equally parsimonious migration histories. See Supplementary Information for a more detailed derivation.

### Metient-evaluate

In Metient-evaluate, weights for each maximum parsimony metric (migrations, comigrations, seeding sites) and optionally, genetic distance and organotropism, are taken as input. These weights are used to rank the solutions on the Pareto front. If no weights are inputted, we provide a pan-cancer parsimony model calibrated to the four cohorts (melanoma, HGSOC, HR-NB, NSCLC) discussed in this work.

### Defining the organotropism matrix

Data from the MSK-MET study^31^ for 25,775 patients with annotations of distant metastases locations was downloaded from the publicly available cbioportal^66^. Each patient had annotations of one of 27 primary cancer types and the presence or absence of a metastasis in one of 21 distant anatomical sites. The original authors extracted this data from electronic health records and mapped it to a reference set of anatomical sites. We sum over all patients to build a 27 x 21, cancer type by metastatic site occurrence matrix. We then normalize the rows to turn these into frequencies. We interpret the negative log frequencies as a “relative time to metastasis”, and only score migrations from the primary site to other sites, because there is no data to indicate frequencies of seeding from metastatic sites to other metastatic sites, or back to the primary. We make this data available for users, with the option for users to instead input their own organotropism vector for each patient.

### Evaluations on simulated data

We use the simulated data for 80 patients provided by MACHINA^17^ to benchmark our method’s performance. To prepare inputs to Metient, we use the same clustering algorithm and clone tree inference algorithm used in MACHINA (MACHINA^17^ and SPRUCE^59^, respectively) in order to accurately compare only our migration history inference algorithm (including polytomy resolution) against MACHINA’s. All performance scores are reported using MACHINA’s PMH-TI mode and Metient-calibrate with a sample size of 1024, both with default configurations. We do not use polytomy resolution for Metient-calibrate in these results, since it does not improve performance on simulated data. (Supplementary Tables 4, 3). However, this performance is not necessarily indicative of polytomy resolution working poorly, because it actually finds more parsimonious solutions than the ground truth solution in 75% of simulated data (Supplementary Figure S10).

#### Evaluation metrics.

We use the same migration graph and seeding clones F1-scores as MACHINA. Given a reconstructed migration graph G, its recall and precision with respect to the ground truth migration graph $G^{*}$ are calculated as follows:

$$\text{recall}=\frac{\left|E\left(G\right)\;\cap \;E\left(G^{*}\right)\right|}{\left|E\left(G^{*}\right)\right|}\;\;\;\;\;\text{precision}=\frac{\left|E\left(G\right)\;\cap \;E\left(G^{*}\right)\right|}{\left|E\left(G\right)\right|}$$

where $E\left(G\right)$ are the edges of G, and multiple edges between the same two sites are included in $E\left(G\right)$. When there are multiple edges from site i to site j, $\left|E\left(G\right)\;\cap \;E\left(G^{*}\right)\right|=\min\left(a,b\right)$, where a and b are the number of edges from site i to site j in G and $G^{*}$, respectively.

Recall and precision of the seeding clones in the inferred migration history (which includes inference of both the clone tree labeling and observed clone proportions) is calculated as follows:

$$\text{recall}=\frac{\left|C\left(U,V\right)\;\cap \;C\left(U^{*},V^{*}\right)\right|}{\left|C\left(U^{*},V^{*}\right)\right|}\;\;\;\;\;\text{precision}=\frac{\left|C\left(U,V\right)\;\cap \;C\left(U^{*},V^{*}\right)\right|}{\left|C\left(U,V\right)\right|}$$

where $C\left(U,V\right)$ is the set of mutations, i.e., the subclone, associated with the clone nodes that have an outgoing migration edge. For example, $C\left(U,V\right)=\text{A},\text{B},\text{C}$ in solution A of Figure S1c. The definition for seeding clones used in these evaluations is distinct from how we define seeding clones in the rest of the paper (“Defining colonizing clones, clonality, and phyleticity” in Methods). Specifically, if there is an edge between two nodes $\left(u,v\right)$, where the labeling of u and v are not equal, we define the seeding clone as v. However in order to consistently compare to MACHINA in these evaluations, we use their definition and define the seeding clone as u. We note that identifying the mutations of v is generally a harder problem.

#### Timing benchmarks.

All timing benchmarks (Figure S3e) were run on 8 Intel(R) Xeon(R) CPU E5–2697 v4 @ 2.30GHz CPU cores with 8 gigabytes of RAM per core. Runtime of each method is the time needed to run inference and save dot files of the inferred migration histories (and for Metient, an additional serialized file with the results of the top k migration histories). We compare MACHINA’s PMH-TI mode to Metient-calibrate with a sample size of 1024, both with default configurations. These are the same modes used to report comparisons in F1-scores. Each value in Figure S3e is the time needed to run one patient’s tree. Because Metient-calibrate has an additional inference step where parsimony metric weights are fit to a cohort, we take the time needed for this additional step and divide it by the number of patient trees in the cohort, and add this time to each patient’s migration history runtime.

### Defining colonizing clones, clonality, and phyleticity

A colonizing clone is defined as a node in a migration history whose parent is a different color than itself. There are two exceptions to this rule: when node a has a parent with a different color than itself, but the node is a witness node (Figure S1c) or a polytomy resolver node (e.g. A_POL in Supplementary Figure S11a). In these cases, these nodes do not represent any new mutations, but rather contain the same mutations as its parent. For these two cases, the colonizing clone is defined to be a ’s parent node.

In order to rectify different meanings of the terms “monoclonal” and “polyclonal” used in previous work, we define two terms:

- genetic clonality: if all sites are seeded by the same colonizing clone, this patient is genetically monoclonal, otherwise, genetically polyclonal.
- site clonality: if each site is seeded by one colonizing clone, but not necessarily the same colonizing clone, this patient is site monoclonal, otherwise, site polyclonal.

Genetic clonality and site clonality are depicted schematically in Figure 2b.

To define phyleticity, we first extract all colonizing clones from a migration history. We then identify the colonizing clone closest to the root, s, i.e., the colonizing clone with the shortest path to the root. If all other colonizing clones are descendants of the tree rooted at s, the migration history is monophyletic, otherwise, it is polyphyletic. Under this definition, if a tree is monophyletic, then there are no independent evolutionary trajectories that give rise to colonizing clones. This is depicted schematically in Figure 2c.

In order to accurately compare our phyleticity measurements to TRACERx, we use their definition in Figure 4c and the TRACERx comparison analysis. To apply their definition to our migration histories, we extract colonizing clones as described above, and then determine if there is a Hamiltonian path in the clone tree that connects the colonizing clones. I.e., we determine if there is a path in the clone tree that visits each colonizing clone exactly once. If such a Hamiltonian path exists, we call this migration history monophyletic under the TRACERx definition, and polyphyletic otherwise.

### Bootstrap sampling for fitting parsimony metric weights

Running Metient-calibrate on the 167 patients from the melanoma, HGSOC, HR-NB and NSCLC datasets infers a Pareto front of migration histories for each patient. For each dataset, we subset patients that have a Pareto front with size greater than one, and take 100 bootstrap samples of patients from this subset. Patients with a single solution on the Pareto front do not have an impact on the cross-entropy loss used to fit the parsimony metric weights. For each bootstrap sample of patients, their Pareto front migration histories are used to fit the parsimony metric weights (“Calibrate alignment” in Supplementary Information). For each of the parsimony metric weights fit to a bootstrap sample, we evaluated how these weights would order the Pareto front, and evaluated how consistently the same top solution was chosen. We average the percent of times the same solution is ranked as the top solution across the four datasets.

## Supplementary Material

Supplement 1

## Data Availability

The results for the HR-NB cohort were based on data under the study phs03111.v1.p1 and were accessed from the NCI’s Cancer Research Data Commons (https://datacommons.cancer.gov). The anatomical site labels in Figure 6 used data generated by the TRAcking Non-small Cell Lung Cancer Evolution Through Therapy (Rx) (TRACERx) Consortium and provided by the UCL Cancer Institute and The Francis Crick Institute. The TRACERx study is sponsored by University College London, funded by Cancer Research UK and coordinated through the Cancer Research UK and UCL Cancer Trials Centre. This data is available from the European Genome-phenome Archive under accession EGAD00001009825^67^. All other data used for the NSCLC cohort were made publicly available and accessed at https://zenodo.org/records/7649257. The organotropism matrix derived from data in the MSK-MET study^31^ is available at https://github.com/morrislab/metient/blob/main/metient/data/msk_met/msk_met_freq_by_cancer_type.csv. The following publicly available datasets were accessed via the supplementary information in the following studies: melanoma^3^, breast^68^, HGSOC^4^, MSK-MET^31^. The lung adenocarcinoma single-cell lineage tracing data were made publicly available and accessed at https://zenodo.org/records/4243162.

## References

[1] Ganesh Karuna and Massagué Joan. Targeting metastatic cancer. Nature medicine, 27(1):34–44, 2021. 10.1038/s41591-020-01195-4 PMC7895475 33442008

[2] Gundem Gunes, Loo Peter Van, Kremeyer Barbara, Alexandrov Ludmil B, Tubio Jose MC, Papaemmanuil Elli, Brewer Daniel S, Kallio Heini ML, Högnäs Gunilla, Annala Matti, The evolutionary history of lethal metastatic prostate cancer. Nature, 520(7547):353–357, 2015. 25830880 10.1038/nature14347 PMC4413032

[3] Zachary Sanborn J, Chung Jongsuk, Purdom Elizabeth, Wang Nicholas J, Kakavand Hojabr, Wilmott James S, Butler Timothy, Thompson John F, Mann Graham J, Haydu Lauren E, Phylogenetic analyses of melanoma reveal complex patterns of metastatic dissemination. Proceedings of the National Academy of Sciences, 112(35):10995–11000, 2015. 10.1073/pnas.1508074112 PMC4568214 26286987

[4] McPherson Andrew, Roth Andrew, Laks Emma, Masud Tehmina, Bashashati Ali, Zhang Allen W, Ha Gavin, Biele Justina, Yap Damian, Wan Adrian, Divergent modes of clonal spread and intraperitoneal mixing in high-grade serous ovarian cancer. Nature genetics, 48(7):758–767, 2016. 27182968 10.1038/ng.3573

[5] Birkbak Nicolai J and McGranahan Nicholas. Cancer genome evolutionary trajectories in metastasis. Cancer cell, 37(1):8–19, 2020. 31935374 10.1016/j.ccell.2019.12.004

[6] Wei Q, Ye Z, Zhong X, Li L, Wang C, Myers RE, Palazzo JP, Fortuna D, Yan A, Waldman SA, Multiregion whole-exome sequencing of matched primary and metastatic tumors revealed genomic heterogeneity and suggested polyclonal seeding in colorectal cancer metastasis. Annals of oncology, 28(9):2135–2141, 2017. 28911083 10.1093/annonc/mdx278 PMC5834069

[7] Hu Zheng, Ding Jie, Ma Zhicheng, Sun Ruping, Seoane Jose A, Scott Shaffer J, Suarez Carlos J, Berghoff Anna S, Cremolini Chiara, Falcone Alfredo, Quantitative evidence for early metastatic seeding in colorectal cancer. Nature genetics, 51(7):1113–1122, 2019. 31209394 10.1038/s41588-019-0423-x PMC6982526

[8] Hu Zheng, Li Zan, Ma Zhicheng, and Curtis Christina. Multi-cancer analysis of clonality and the timing of systemic spread in paired primary tumors and metastases. Nature genetics, 52(7):701–708, 2020. 32424352 10.1038/s41588-020-0628-z PMC7343625

[9] Gundem Gunes, Levine Max F, Roberts Stephen S, Cheung Irene Y, Medina-Martínez Juan S, Feng Yi, Arango-Ossa Juan E, Chadoutaud Loic, Rita Mathieu, Asimomitis Georgios, Clonal evolution during metastatic spread in high-risk neuroblastoma. Nature Genetics, pages 1–12, 2023. 10.1038/s41588-023-01395-x PMC11481711 37169874

[10] Brown David, Smeets Dominiek, Székely Borbála, Larsimont Denis, Marcell Szász A, Adnet Pierre-Yves, Rothé Françoise, Rouas Ghizlane, Nagy Zsófia I, Faragó Zsófia, Phylogenetic analysis of metastatic progression in breast cancer using somatic mutations and copy number aberrations. Nature communications, 8(1):14944, 2017. 10.1038/ncomms14944 PMC5474888 28429735

[11] Brastianos Priscilla K, Carter Scott L, Santagata Sandro, Cahill Daniel P, Taylor-Weiner Amaro, Jones Robert T, Van Allen Eliezer M, Lawrence Michael S, Horowitz Peleg M, Cibulskis Kristian, Genomic characterization of brain metastases reveals branched evolution and potential therapeutic targets. Cancer discovery, 5(11):1164–1177, 2015. 26410082 10.1158/2159-8290.CD-15-0369 PMC4916970

[12] Turajlic Samra, Xu Hang, Litchfield Kevin, Rowan Andrew, Chambers Tim, Lopez Jose I, Nicol David, O’Brien Tim, Larkin James, Horswell Stuart, Tracking cancer evolution reveals constrained routes to metastases: Tracerx renal. Cell, 173(3):581–594, 2018. 29656895 10.1016/j.cell.2018.03.057 PMC5938365

[13] Noorani Ayesha, Li Xiaodun, Goddard Martin, Crawte Jason, Alexandrov Ludmil B, Secrier Maria, Eldridge Matthew D, Bower Lawrence, Weaver Jamie, Lao-Sirieix Pierre, Genomic evidence supports a clonal diaspora model for metastases of esophageal adenocarcinoma. Nature genetics, 52(1):74–83, 2020. 31907488 10.1038/s41588-019-0551-3 PMC7100916

[14] Bakir Maise Al, Huebner Ariana, Martínez-Ruiz Carlos, Grigoriadis Kristiana, Watkins Thomas B. K., Pich Oriol, Moore David A., Veeriah Selvaraju, Ward Sophia, Laycock Joanne, and The evolution of non-small cell lung cancer metastases in tracerx. Nature, Apr 2023. 10.1038/s41586-023-05729-x PMC10115651 37046095

[15] Dang HX, White BS, Foltz SM, Miller CA, Luo Jingqin, Fields RC, and Maher CA. Clonevol: clonal ordering and visualization in cancer sequencing. Annals of oncology, 28(12):3076–3082, 2017. 28950321 10.1093/annonc/mdx517 PMC5834020

[16] Reiter Johannes G, Makohon-Moore Alvin P, Gerold Jeffrey M, Bozic Ivana, Chatterjee Krishnendu, Iacobuzio-Donahue Christine A, Vogelstein Bert, and Nowak Martin A. Reconstructing metastatic seeding patterns of human cancers. Nature communications, 8(1):14114, 2017. 10.1038/ncomms14114 PMC5290319 28139641

[17] El-Kebir Mohammed, Satas Gryte, and Raphael Benjamin J. Inferring parsimonious migration histories for metastatic cancers. Nature genetics, 50(5):718–726, 2018. 29700472 10.1038/s41588-018-0106-z PMC6103651

[18] Kumar Sudhir, Chroni Antonia, Tamura Koichiro, Sanderford Maxwell, Oladeinde Olumide, Aly Vivian, Vu Tracy, and Miura Sayaka. Pathfinder: Bayesian inference of clone migration histories in cancer. Bioinformatics, 36(Supplement_2):i675–i683, 2020. 33381835 10.1093/bioinformatics/btaa795 PMC7773489

[19] Sankoff David. Minimal mutation trees of sequences. SIAM Journal on Applied Mathematics, 28(1):35–42, 1975.

[20] Fitch Walter M. Toward defining the course of evolution: minimum change for a specific tree topology. Systematic Biology, 20(4):406–416, 1971.

[21] Hartigan John A. Minimum mutation fits to a given tree. Biometrics, pages 53–65, 1973.

[22] Quinn Jeffrey J, Jones Matthew G, Okimoto Ross A, Nanjo Shigeki, Chan Michelle M, Yosef Nir, Bivona Trever G, and Weissman Jonathan S. Single-cell lineages reveal the rates, routes, and drivers of metastasis in cancer xenografts. Science, 371(6532):eabc1944, 2021. 33479121 10.1126/science.abc1944 PMC7983364

[23] Zhang Chong, Zhang Lin, Xu Tianlei, Xue Ruidong, Yu Liang, Zhu Yuelu, Wu Yunlong, Zhang Qingqing, Li Dongdong, Shen Shuohao, Mapping the spreading routes of lymphatic metastases in human colorectal cancer. Nature communications, 11(1):1993, 2020. 10.1038/s41467-020-15886-6 PMC7181746 32332722

[24] Naxerova Kamila, Reiter Johannes G, Brachtel Elena, Lennerz Jochen K, Wetering Marc Van De, Rowan Andrew, Cai Tianxi, Clevers Hans, Swanton Charles, Nowak Martin A, Origins of lymphatic and distant metastases in human colorectal cancer. Science, 357(6346):55–60, 2017. 28684519 10.1126/science.aai8515 PMC5536201

[25] Yamamoto Ami, Doak Andrea E, and Cheung Kevin J. Orchestration of collective migration and metastasis by tumor cell clusters. Annual Review of Pathology: Mechanisms of Disease, 18(1):231–256, 2023. 10.1146/annurev-pathmechdis-031521-023557 36207009

[26] Gurobi Optimization, LLC. Gurobi Optimizer Reference Manual, 2023.

[27] Stiglitz Joseph E. Pareto optimality and competition. The Journal of Finance, 36(2):235–251, 1981.

[28] Lengyel Ernst. Ovarian cancer development and metastasis. The American journal of pathology, 177(3):1053–1064, 2010. 20651229 10.2353/ajpath.2010.100105 PMC2928939

[29] Mitra Anirban K. Ovarian cancer metastasis: a unique mechanism of dissemination. IntechOpen, 2016.

[30] Gui Philippe and Bivona Trever G. Evolution of metastasis: New tools and insights. Trends in Cancer, 8(2):98–109, 2022. 34872888 10.1016/j.trecan.2021.11.002

[31] Nguyen Bastien, Fong Christopher, Luthra Anisha, Smith Shaleigh A, DiNatale Renzo G, Nandakumar Subhiksha, Walch Henry, Chatila Walid K, Madupuri Ramyasree, Kundra Ritika, Genomic characterization of metastatic patterns from prospective clinical sequencing of 25,000 patients. Cell, 185(3):563–575, 2022. 35120664 10.1016/j.cell.2022.01.003 PMC9147702

[32] Jee Justin, Fong Christopher, Pichotta Karl, Tran Thinh Ngoc, Luthra Anisha, Waters Michele, Fu Chenlian, Altoe Mirella, Liu Si-Yang, Maron Steven B, Automated real-world data integration improves cancer outcome prediction. Nature, pages 1–9, 2024. 10.1038/s41586-024-08167-5 PMC11655358 39506116

[33] Reticker-Flynn Nathan E, Zhang Weiruo, Belk Julia A, Basto Pamela A, Escalante Nichole K, Pilarowski Genay OW, Bejnood Alborz, Martins Maria M, Kenkel Justin A, Linde Ian L, Lymph node colonization induces tumor-immune tolerance to promote distant metastasis. Cell, 185(11):1924–1942, 2022. 35525247 10.1016/j.cell.2022.04.019 PMC9149144

[34] Williams Marc J, Werner Benjamin, Barnes Chris P, Graham Trevor A, and Sottoriva Andrea. Identification of neutral tumor evolution across cancer types. Nature genetics, 48(3):238–244, 2016. 26780609 10.1038/ng.3489 PMC4934603

[35] Taddei François, Radman Miroslav, Maynard-Smith John, Toupance Bruno, Gouyon Pierre-Henri, and Godelle Bernard. Role of mutator alleles in adaptive evolution. Nature, 387(6634):700–702, 1997. 9192893 10.1038/42696

[36] Mao Emily F, Lane Laura, Lee Jean, and Miller Jeffrey H. Proliferation of mutators in a cell population. Journal of bacteriology, 179(2):417–422, 1997. 8990293 10.1128/jb.179.2.417-422.1997 PMC178711

[37] Gentile Christopher F, Yu Szi-Chieh, Serrano Sebastian Akle, Gerrish Philip J, and Sniegowski Paul D. Competition between high-and higher-mutating strains of escherichia coli. Biology letters, 7(3):422–424, 2011. 21227974 10.1098/rsbl.2010.1036 PMC3097864

[38] Christensen Ditte S, Ahrenfeldt Johanne, Sokač Mateo, Kisistók Judit, Thomsen Martin K, Maretty Lasse, McGranahan Nicholas, and Birkbak Nicolai J. Treatment represents a key driver of metastatic cancer evolution. Cancer Research, 82(16):2918–2927, 2022. 35731928 10.1158/0008-5472.CAN-22-0562

[39] Martínez-Jiménez Francisco, Movasati Ali, Brunner Sascha Remy, Nguyen Luan, Priestley Peter, Cuppen Edwin, and Hoeck Arne Van. Pan-cancer whole-genome comparison of primary and metastatic solid tumours. Nature, pages 1–9, 2023. 10.1038/s41586-023-06054-z PMC10247378 37165194

[40] Gao Yang, Bado Igor, Wang Hai, Zhang Weijie, Rosen Jeffrey M, and Zhang Xiang H-F. Metastasis organotropism: redefining the congenial soil. Developmental cell, 49(3):375–391, 2019. 31063756 10.1016/j.devcel.2019.04.012 PMC6506189

[41] Alvord Ellsworth C. Why do gliomas not metastasize? Archives of Neurology, 33(2):73–75, 1976. 1252152 10.1001/archneur.1976.00500020001001

[42] Wolf Ingrid H, Richtig Erika, Kopera Daisy, and Kerl Helmut. Locoregional cutaneous metastases of malignant melanoma and their management. Dermatologic surgery, 30:244–247, 2004. 14871216 10.1111/j.1524-4725.2004.30091.x

[43] Sleeman Jonathan, Schmid Anja, and Thiele Wilko. Tumor lymphatics. In Seminars in cancer biology, volume 19, pages 285–297. Elsevier, 2009. 19482087 10.1016/j.semcancer.2009.05.005

[44] Lee Yeu-Tsu Margaret and Geer Deborah A. Primary liver cancer: pattern of metastasis. Journal of surgical oncology, 36(1):26–31, 1987. 3041113 10.1002/jso.2930360107

[45] Wu Wenrui, He Xingkang, Andayani Dewi, Yang Liya, Ye Jianzhong, Li Yating, Chen Yanfei, and Li Lanjuan. Pattern of distant extrahepatic metastases in primary liver cancer: a seer based study. Journal of Cancer, 8(12):2312, 2017. 28819435 10.7150/jca.19056 PMC5560150

[46] Riihimäki Matias, Hemminki A, Fallah Mahdi, Thomsen Hauke, Sundquist Kristina, Sundquist Jan, and Hemminki Kari. Metastatic sites and survival in lung cancer. Lung cancer, 86(1):78–84, 2014. 25130083 10.1016/j.lungcan.2014.07.020

[47] Bado Igor L, Zhang Weijie, Hu Jingyuan, Xu Zhan, Wang Hai, Sarkar Poonam, Li Lucian, Wan Ying-Wooi, Liu Jun, Wu William, The bone microenvironment increases phenotypic plasticity of er+ breast cancer cells. Developmental cell, 56(8):1100–1117, 2021. 33878299 10.1016/j.devcel.2021.03.008 PMC8062036

[48] Zhang Weijie, Bado Igor L, Hu Jingyuan, Wan Ying-Wooi, Wu Ling, Wang Hai, Gao Yang, Jeong Hyun-Hwan, Xu Zhan, Hao Xiaoxin, The bone microenvironment invigorates metastatic seeds for further dissemination. Cell, 184(9):2471–2486, 2021. 33878291 10.1016/j.cell.2021.03.011 PMC8087656

[49] Sanghvi Neel, Calvo-Alcañiz Camilo, Rajagopal Padma S, Scalera Stefano, Canu Valeria, Sinha Sanju, Schischlik Fiorella, Wang Kun, Madan Sanna, Shulman Eldad, Charting the transcriptomic landscape of primary and metastatic cancers in relation to their origin and target normal tissues. Science Advances, 10(49):eadn0220, 2024. 39642223 10.1126/sciadv.adn0220 PMC11623296

[50] Lemjabbar-Alaoui Hassan, Hassan Omer UI, Yang Yi-Wei, and Buchanan Petra. Lung cancer: Biology and treatment options. Biochimica et Biophysica Acta (BBA)-Reviews on Cancer, 1856(2):189–210, 2015. 26297204 10.1016/j.bbcan.2015.08.002 PMC4663145

[51] Pereira Ethel R, Kedrin Dmitriy, Seano Giorgio, Gautier Olivia, Meijer Eelco FJ, Jones Dennis, Chin Shan-Min, Kitahara Shuji, Bouta Echoe M, Chang Jonathan, Lymph node metastases can invade local blood vessels, exit the node, and colonize distant organs in mice. Science, 359(6382):1403–1407, 2018. 29567713 10.1126/science.aal3622 PMC6002772

[52] Cheung Kevin J and Ewald Andrew J. A collective route to metastasis: Seeding by tumor cell clusters. Science, 352(6282):167–169, 2016. 27124449 10.1126/science.aaf6546 PMC8183671

[53] Ashley Charles W, Paula Arnaud Da Cruz, Kumar Rahul, Mandelker Diana, Pei Xin, Riaz Nadeem, Reis-Filho Jorge S, and Weigelt Britta. Analysis of mutational signatures in primary and metastatic endometrial cancer reveals distinct patterns of dna repair defects and shifts during tumor progression. Gynecologic oncology, 152(1):11–19, 2019. 30415991 10.1016/j.ygyno.2018.10.032 PMC6726428

[54] Angus Lindsay, Smid Marcel, Wilting Saskia M, Riet Job van, Hoeck Arne Van, Nguyen Luan, Nik-Zainal Serena, Steenbruggen Tessa G, Tjan-Heijnen Vivianne CG, Labots Mariette, The genomic landscape of metastatic breast cancer highlights changes in mutation and signature frequencies. Nature genetics, 51(10):1450–1458, 2019. 31570896 10.1038/s41588-019-0507-7 PMC6858873

[55] Satas Gryte, Zaccaria Simone, El-Kebir Mohammed, and Raphael Benjamin J. Decifering the elusive cancer cell fraction in tumor heterogeneity and evolution. Cell systems, 12(10):1004–1018, 2021. 34416171 10.1016/j.cels.2021.07.006 PMC8542635

[56] Jamal-Hanjani Mariam, Wilson Gareth A, McGranahan Nicholas, Birkbak Nicolai J, Watkins Thomas BK, Veeriah Selvaraju, Shafi Seema, Johnson Diana H, Mitter Richard, Rosenthal Rachel, Tracking the evolution of non–small-cell lung cancer. New England Journal of Medicine, 376(22):2109–2121, 2017. 28445112 10.1056/NEJMoa1616288

[57] Kulman Ethan, Kuang Rui, and Morris Quaid. Orchard: building large cancer phylogenies using stochastic combinatorial search. arXiv preprint arXiv:2311.12917, 2023. 10.1371/journal.pcbi.1012653 PMC11723595 39775053

[58] Wintersinger Jeff A, Dobson Stephanie M, Kulman Ethan, Stein Lincoln D, Dick John E, and Morris Quaid. Reconstructing complex cancer evolutionary histories from multiple bulk dna samples using pairtreereconstructing cancer evolutionary histories using pairtree. Blood Cancer Discovery, pages OF1–OF12, 2022. 10.1158/2643-3230.BCD-21-0092 PMC9780082 35247876

[59] El-Kebir Mohammed, Satas Gryte, Oesper Layla, and Raphael Benjamin J. Inferring the mutational history of a tumor using multi-state perfect phylogeny mixtures. Cell systems, 3(1):43–53, 2016. 27467246 10.1016/j.cels.2016.07.004

[60] Malikic Salem, McPherson Andrew W, Donmez Nilgun, and Sahinalp Cenk S. Clonality inference in multiple tumor samples using phylogeny. Bioinformatics, 31(9):1349–1356, 2015. 25568283 10.1093/bioinformatics/btv003

[61] Ray Surjyendu, Jia Bei, Safavi Sam, Opijnen Tim van, Isberg Ralph, Rosch Jason, and Bento José. Exact inference under the perfect phylogeny model. arXiv preprint arXiv:1908.08623, 2019.

[62] Jia Bei, Ray Surjyendu, Safavi Sam, and Bento José. Efficient projection onto the perfect phylogeny model. Advances in Neural Information Processing Systems, 31, 2018.

[63] Jang Eric, Gu Shixiang, and Poole Ben. Categorical reparameterization with gumbel-softmax. arXiv preprint arXiv:1611.01144, 2016.

[64] Maddison Chris J, Mnih Andriy, and Teh Yee Whye. The concrete distribution: A continuous relaxation of discrete random variables. arXiv preprint arXiv:1611.00712, 2016.

[65] Li Yaoxin, Liu Jing, Lin Guozheng, Hou Yueyuan, Mou Muyun, and Zhang Jiang. Gumbel-softmax-based optimization: a simple general framework for optimization problems on graphs. Computational Social Networks, 8(1):1–16, 2021.

[66] Gao Jianjiong, Aksoy Bülent Arman, Dogrusoz Ugur, Dresdner Gideon, Gross Benjamin, Onur Sumer S, Sun Yichao, Jacobsen Anders, Sinha Rileen, Larsson Erik, Integrative analysis of complex cancer genomics and clinical profiles using the cbioportal. Science signaling, 6(269):pl1–pl1, 2013. 23550210 10.1126/scisignal.2004088 PMC4160307

[67] Frankell Alexander M, Dietzen Michelle, Bakir Maise Al, Lim Emilia L, Karasaki Takahiro, Ward Sophia, Veeriah Selvaraju, Colliver Emma, Huebner Ariana, Bunkum Abigail, The evolution of lung cancer and impact of subclonal selection in tracerx. Nature, 616(7957):525–533, 2023. 37046096 10.1038/s41586-023-05783-5 PMC10115649

[68] Hoadley Katherine A, Siegel Marni B, Kanchi Krishna L, Miller Christopher A, Ding Li, Zhao Wei, He Xiaping, Parker Joel S, Wendl Michael C, Fulton Robert S, Tumor evolution in two patients with basal-like breast cancer: a retrospective genomics study of multiple metastases. PLoS medicine, 13(12):e1002174, 2016. 27923045 10.1371/journal.pmed.1002174 PMC5140046

[69] Tarabichi Maxime, Salcedo Adriana, Deshwar Amit G, Leathlobhair Máire Ni, Wintersinger Jeff, Wedge David C, Loo Peter Van, Morris Quaid D, and Boutros Paul C. A practical guide to cancer subclonal reconstruction from dna sequencing. Nature methods, 18(2):144–155, 2021. 33398189 10.1038/s41592-020-01013-2 PMC7867630

[70] Roth Andrew, Khattra Jaswinder, Yap Damian, Wan Adrian, Laks Emma, Biele Justina, Ha Gavin, Aparicio Samuel, Bouchard-Côté Alexandre, and Shah Sohrab P. Pyclone: statistical inference of clonal population structure in cancer. Nature methods, 11(4):396–398, 2014. 24633410 10.1038/nmeth.2883 PMC4864026

[71] Gillis Sierra and Roth Andrew. Pyclone-vi: scalable inference of clonal population structures using whole genome data. BMC bioinformatics, 21:1–16, 2020. 33302872 10.1186/s12859-020-03919-2 PMC7730797

[72] Nik-Zainal Serena, Loo Peter Van, Wedge David C, Alexandrov Ludmil B, Greenman Christopher D, Lau King Wai, Raine Keiran, Jones David, Marshall John, Ramakrishna Manasa, The life history of 21 breast cancers. Cell, 149(5):994–1007, 2012. 22608083 10.1016/j.cell.2012.04.023 PMC3428864

[73] Jones Matthew G, Khodaverdian Alex, Quinn Jeffrey J, Chan Michelle M, Hussmann Jeffrey A, Wang Robert, Xu Chenling, Weissman Jonathan S, and Yosef Nir. Inference of single-cell phylogenies from lineage tracing data using cassiopeia. Genome biology, 21:1–27, 2020. 10.1186/s13059-020-02000-8 PMC7155257 32290857

[74] Alexander Wolf F, Angerer Philipp, and Theis Fabian J. Scanpy: large-scale single-cell gene expression data analysis. Genome biology, 19:1–5, 2018. 29409532 10.1186/s13059-017-1382-0 PMC5802054

