# Supplementary material for: Inferring cancer type-specific patterns of metastatic spread
Source: bioRxiv. 2024 Sep 3:2024.07.09.602790. Preprint. [Version 2] doi: 10.1101/2024.07.09.602790 (PMC11398359; doi:10.1101/2024.07.09.602790)
Supplement: Supplementary file 1 [file NIHPP2024.07.09.602790v2-supplement-1.pdf]

bioRxiv preprint doi: https://doi.org/10.1101/2024.07.09.602790; this version posted September 3, 2024. The copyright holder for this preprint (which was not certified by peer review) is the author/funder, who has granted bioRxiv a license to display the preprint in perpetuity. It is made available under aCC-BY 4.0 International license.

Supplementary Figures and Tables

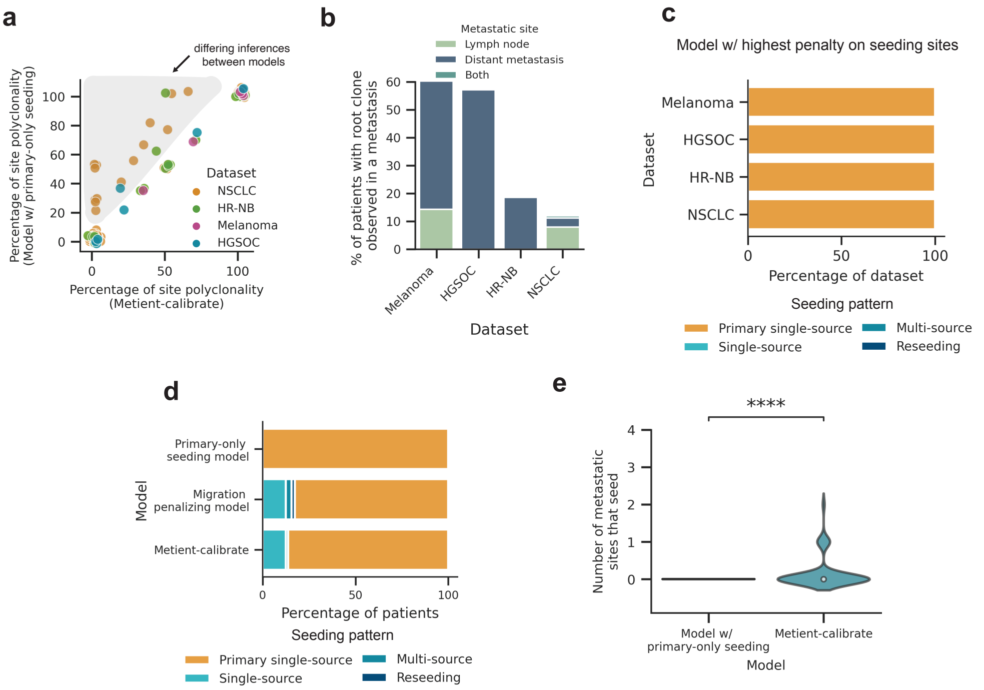

Figure S1. **(a)** A comparison of the percent of site polyclonal migrations for each patient's migration history when using the best migration history chosen by Metient (x-axis) vs. a model that assumes primary-only seeding (y-axis). **(b)** Percent of patients in each dataset with the root cancerous clone observed in a metastatic site. **(c)** The distribution of seeding patterns in each dataset when taking the migration history on the approximate Pareto front with the lowest number of seeding sites, run with Metient-calibrate. **(d)** The distribution of seeding patterns across all patients if we choose the migration history on the Pareto front with the lowest number of seeding sites (primary-only seeding model), lowest number of migrations (migration penalizing model), or the top Metient-calibrate solution. **(e)** A comparison of the number of metastatic sites that seed other sites between migration histories chosen by a model which chooses the migration history with a model that assumes primary-only seeding vs. Metient. Statistical significance assessed by a paired t-test, p=2.233e-06.

bioRxiv preprint doi: https://doi.org/10.1101/2024.07.09.602790; this version posted September 3, 2024. The copyright holder for this preprint (which was not certified by peer review) is the author/funder, who has granted bioRxiv a license to display the preprint in perpetuity. It is made available under aCC-BY 4.0 International license.

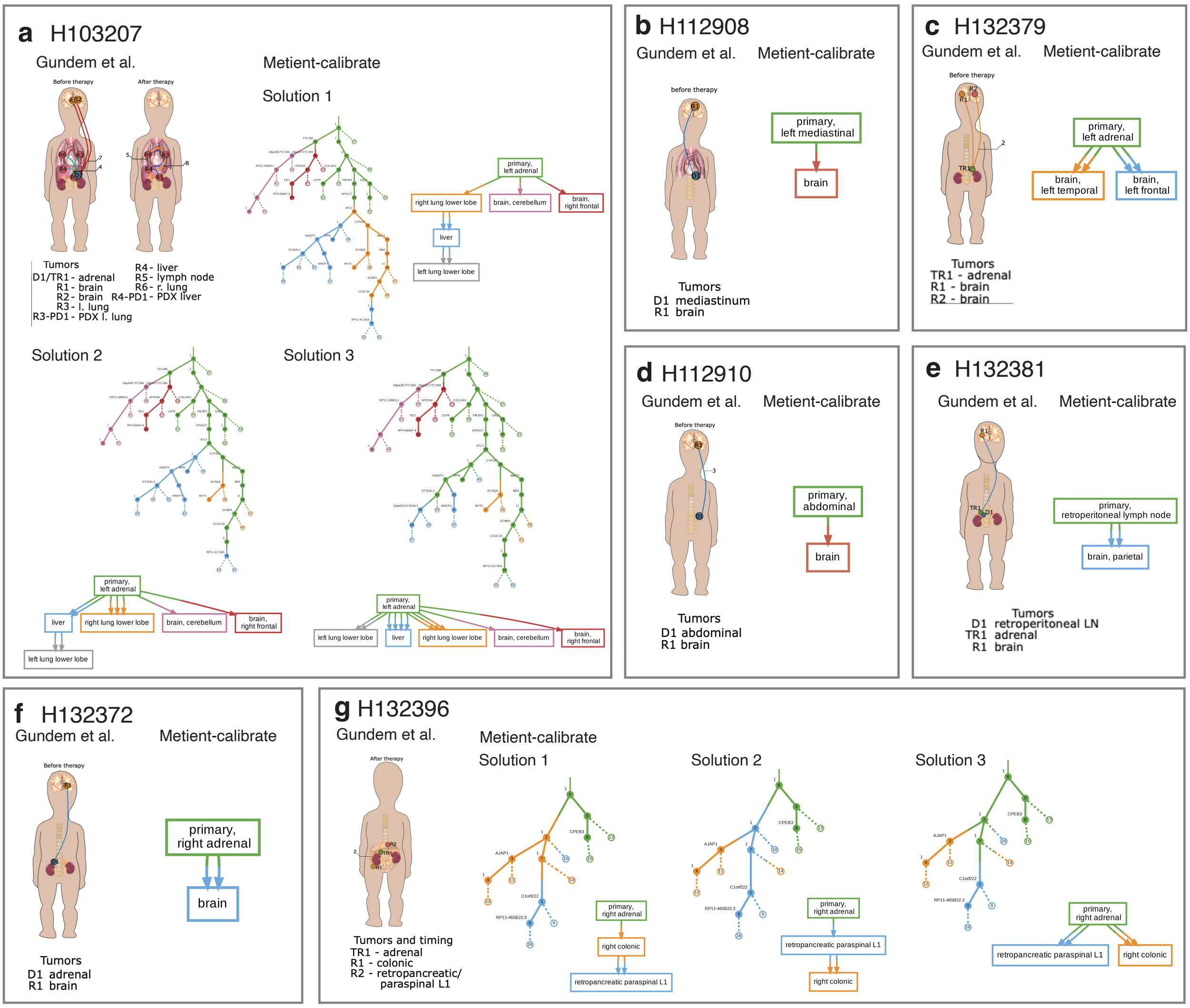

Figure S2. Comparison of Gundem et al.[9] reported body maps (left of each square) and Metient-calibrate inferred histories. The Metient-calibrate solutions with unique migration graphs on the Pareto front are shown. For example, in cases where there are multiple Pareto optimal migration histories with the same migration graph, only the migration history with the lowest loss is shown.

bioRxiv preprint doi: https://doi.org/10.1101/2024.07.09.602790; this version posted September 3, 2024. The copyright holder for this preprint (which was not certified by peer review) is the author/funder, who has granted bioRxiv a license to display the preprint in perpetuity. It is made available under aCC-BY 4.0 International license.

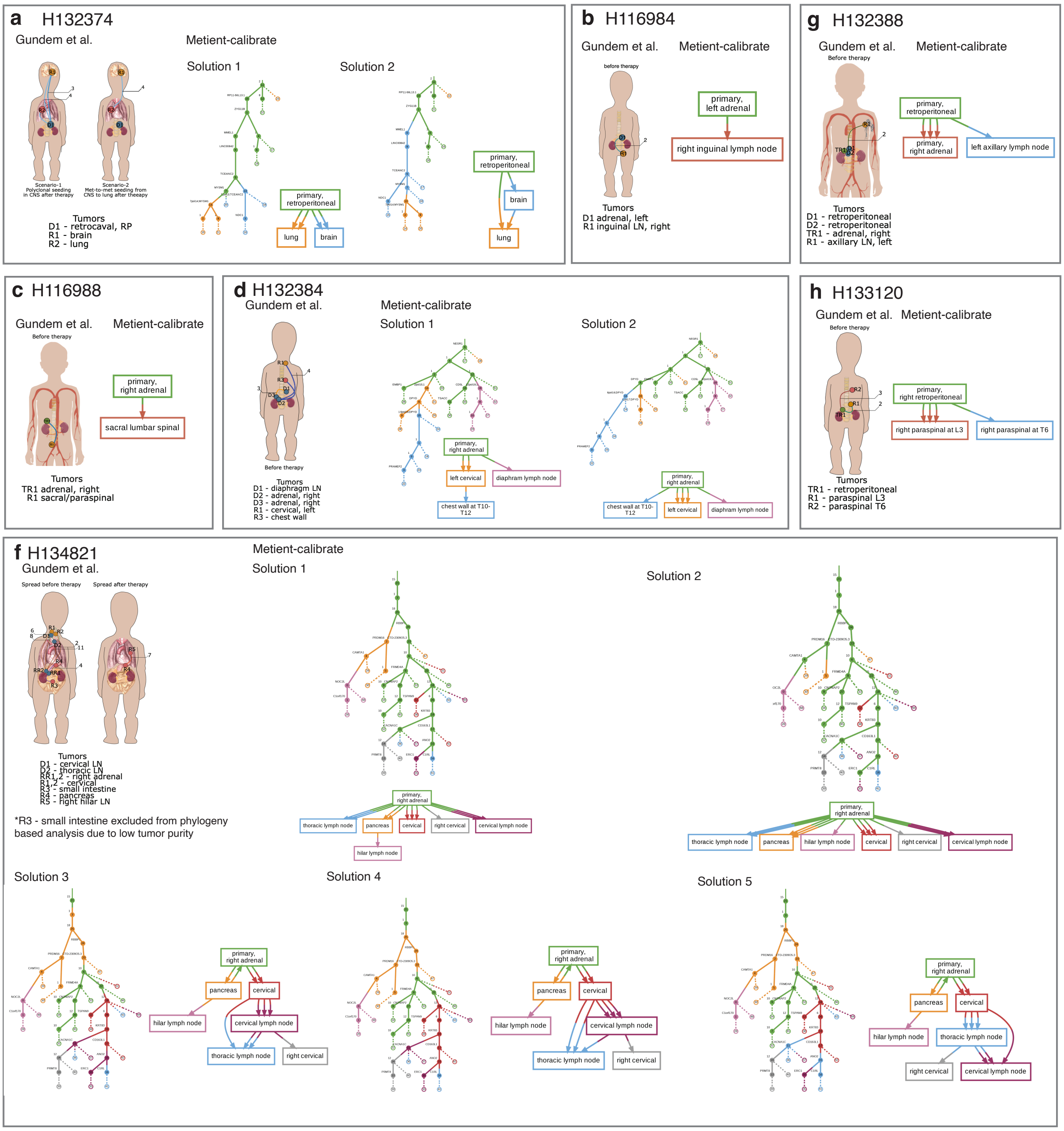

Figure S3. Comparison of Gundem et al.[9] reported body maps (left of each square) and Metient-calibrate inferred histories. The Metient-calibrate solutions with unique migration graphs on the Pareto front are shown. For example, in cases where there are multiple Pareto optimal migration histories with the same migration graph, only the migration history with the lowest loss is shown.

bioRxiv preprint doi: https://doi.org/10.1101/2024.07.09.602790; this version posted September 3, 2024. The copyright holder for this preprint (which was not certified by peer review) is the author/funder, who has granted bioRxiv a license to display the preprint in perpetuity. It is made available under aCC-BY 4.0 International license.

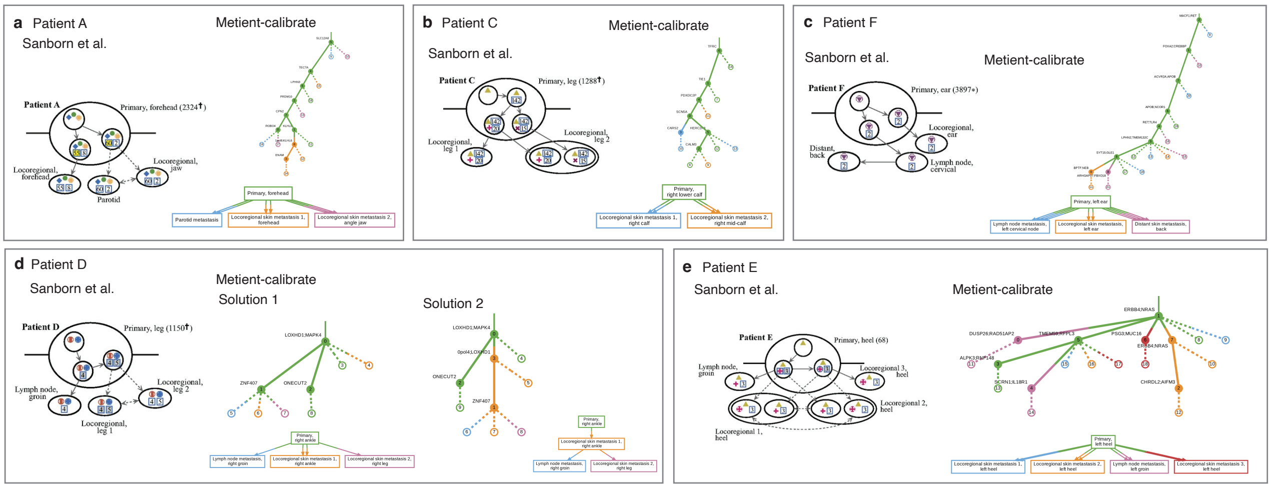

Figure S4. Comparison of Sanborn et al.[3] reported histories and Metient-calibrate inferred histories. In the Sanborn et al.[3] reported histories, solid lines denote probable dissemination patterns and dashed lines denote multiple possible paths. The Metient-calibrate solutions with unique migration graphs on the Pareto front are shown. For example, in cases where there are multiple Pareto optimal migration histories with the same migration graph, only the migration history with the lowest loss is shown.

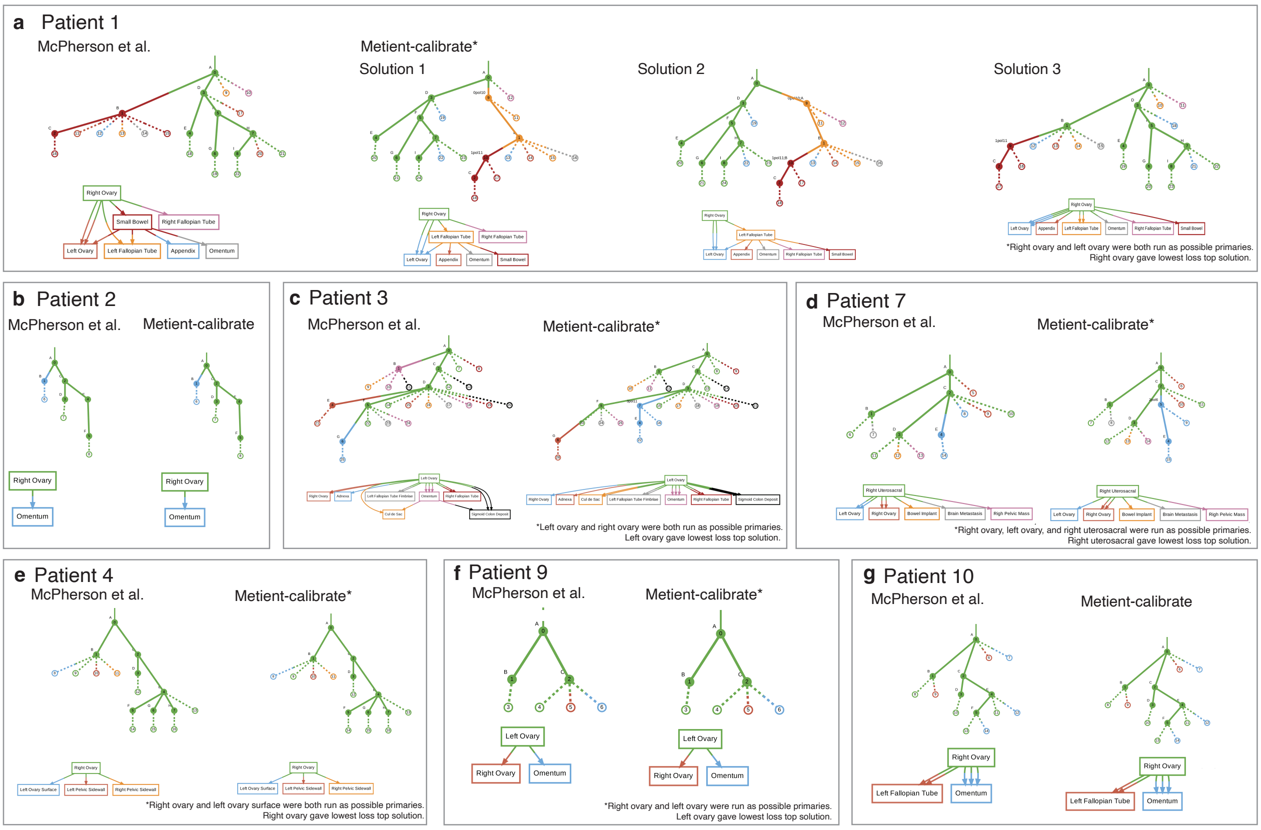

Figure S5. Comparison of McPherson et al.[4] reported histories and Metient-calibrate inferred histories. The Metient-calibrate solutions with unique migration graphs on the Pareto front are shown. For example, in cases where there are multiple Pareto optimal migration histories with the same migration graph, only the migration history with the lowest loss is shown. When multiple possible primaries were available, Metient-calibrate was run once with each possible primary, and the primary with the lowest loss solution is shown.

bioRxiv preprint doi: https://doi.org/10.1101/2024.07.09.602790; this version posted September 3, 2024. The copyright holder for this preprint (which was not certified by peer review) is the author/funder, who has granted bioRxiv a license to display the preprint in perpetuity. It is made available under aCC-BY 4.0 International license.

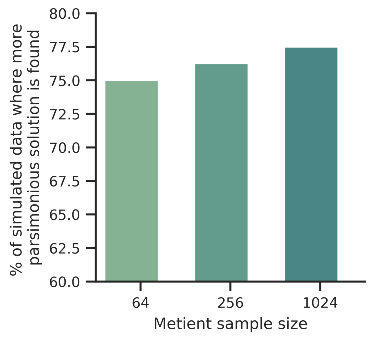

Figure S6. The percent of simulated data where a more parsimonious solution than ground truth is found when running Metient-1024 in calibrate mode with polytomy resolution. More parsimonious is defined as at least one of the parsimony metrics (migration, comigration and seeding site number) being less than the ground truth and all other metrics being equal.

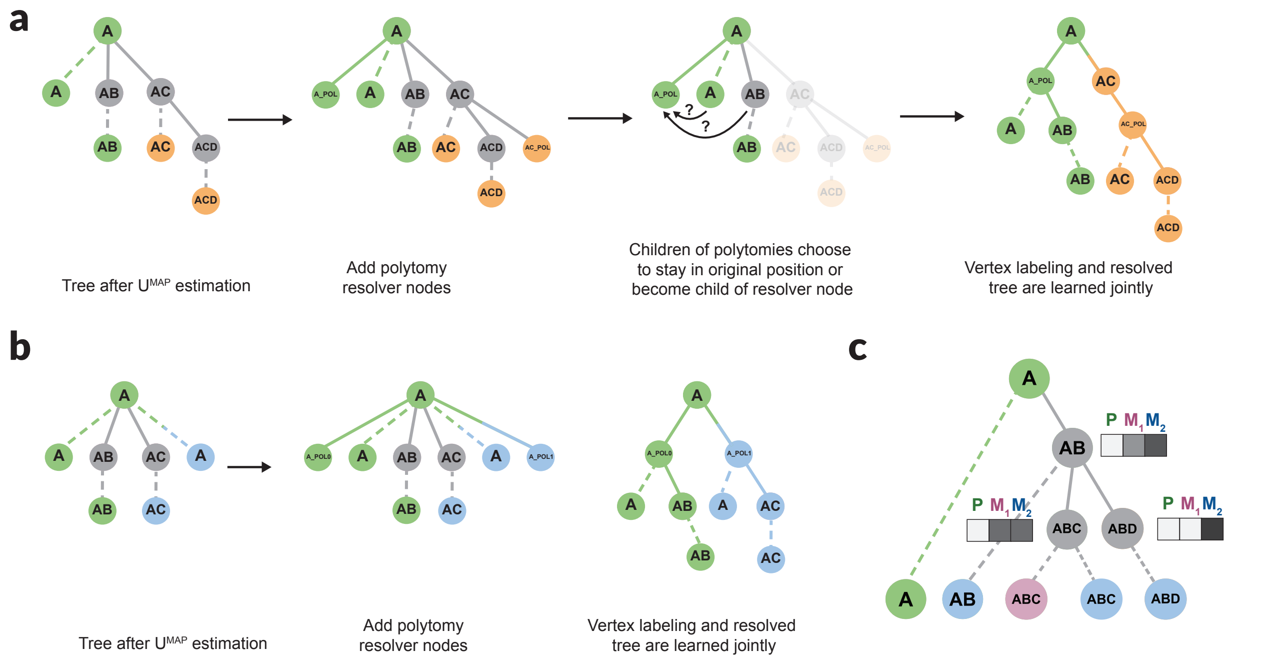

Figure S7. (a) Polytomy resolution algorithm with two nodes (A and AC) that have polytomies that can be resolved. **(b)** Polytomy resolution algorithm for a single node with four children and thus two resolver nodes. **(c)** Weight initialization is done such that nodes start with higher probabilities of being in the same site as the site that they or their children are detected in (after $\mathbf{U}^{\text{MAP}}$ estimation).

bioRxiv preprint doi: https://doi.org/10.1101/2024.07.09.602790; this version posted September 3, 2024. The copyright holder for this preprint (which was not certified by peer review) is the author/funder, who has granted bioRxiv a license to display the preprint in perpetuity. It is made available under aCC-BY 4.0 International license.

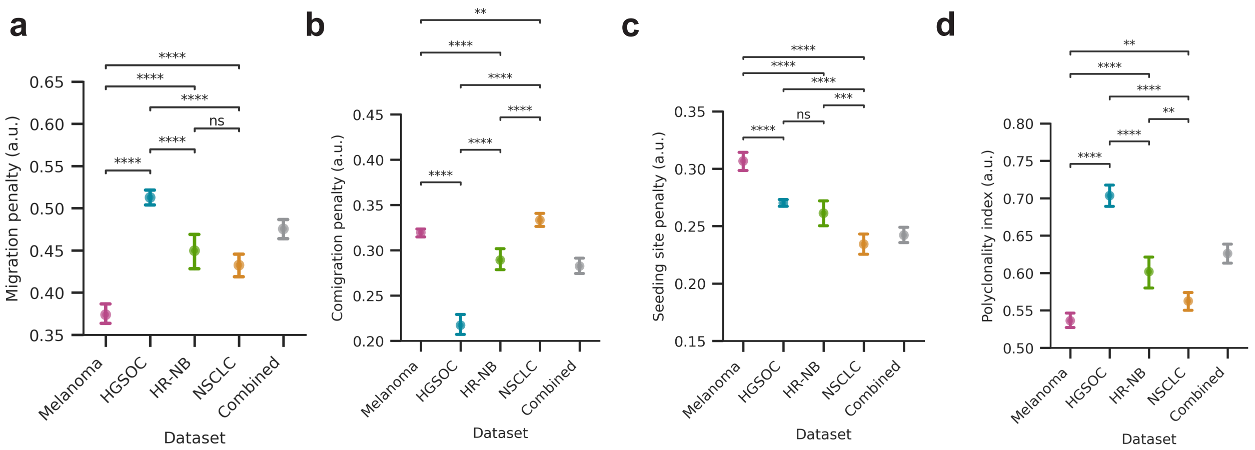

Figure S8. The **(a)** migration penalty/weight, **(b)** comigration penalty/weight, and **(c)** seeding site penalty/weight for each cohort, when taking 100 bootstrap samples of each cohort and fitting the weights to the bootstrapped sample. **(d)** The polyclonality index, which is $1-(w_c/(w_m+w_c))$, where w_m is the migration penalty/weight and w_c is the comgiration penalty/weight. Statistical significance tested through a Welch's t-test; ns: 5e-02 < p <= 1, *: 1e-02 < p <= 5e-02, **: 1e-03 < p <= 1e-02, ***: 1e-04 < p <= 1e-03, ****: p <= 1e-04.

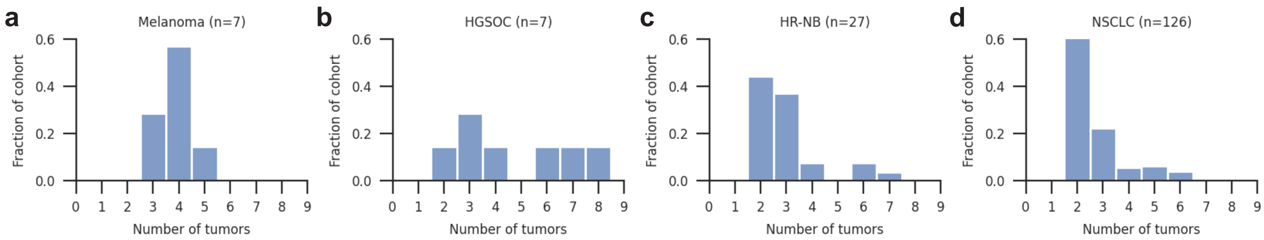

Figure S9. The distribution of tumors (number of distinct anatomical sites) for each cohort: **(a)** melanoma, **(b)** high-grade serous ovarian cancer (HGSOC), **(a)** high-risk neuroblastoma (HR-NB) and **(a)** non-small cell lung cancer (NSCLC).

Previous Methods for Migration History Inference

Method	Labels clone tree	Estimates clone proportions in sites	Models Complex Seeding	Multiple solutions	Organo-tropism	Genetic Distance	Polytomy Resolution
ClonEvol[15]	Y	Y	N	Y	N	N	N
Treeomics[16]	N	Y	N	N	N	N	N
MACHINA[17]	Y	Y	Y	N	N	N	Y
PathFinder[40]	Y	N	N	Y	N	Y	Y
Metient	Y	Y	Y	Y	Y	Y	Y

Table 1. Summary of previous methods which perform some aspect of migration history inference. Y = yes, N = no. Labels clone tree refers to whether the method infers the labels of the internal vertices of a clone tree (e.g. labeling clone AB as originating in lymph in Figure 1c, solution A). Estimates clone proportions in sites refers to whether the method infers the leaf nodes (witness nodes) (e.g. identifying that clone ABC is present in both lymph and liver in Figure 1c, solution A). Multiple solutions indicates whether a method outputs multiple possible migration histories.

bioRxiv preprint doi: https://doi.org/10.1101/2024.07.09.602790; this version posted September 3, 2024. The copyright holder for this preprint (which was not certified by peer review) is the author/funder, who has granted bioRxiv a license to display the preprint in perpetuity. It is made available under aCC-BY 4.0 International license.

Parsimony model	Migration number weight (w_m)	Comigration number weight (w_c)	Seeding site number weight (w_s)
$w_m >> w_c >> w_s$ (MACHINA model)	1000	100	1
$w_c >> w_m >> w_s$	100	1000	1
$w_s >> w_m >> w_c$	100	1	1000
$w_s >> w_c >> w_m$	1	100	1000
$w_c >> w_s >> w_m$	1	1000	100
$w_m >> w_s >> w_c$	1000	1	100

Table 2. The multiple parsimony models that Metient uses to build a Pareto front of solutions for a patient's data. Each parsimony model has a different relative weighting on each parsimony metric.

Average migration graph F1-scores

Method	Primary-only	Met-to-met	Macro-F1	Micro-F1
Evaluate (MP)	0.930	0.688	0.809	0.736
Evaluate (MP) + polyres	**0.983**	0.648	0.816	0.715
Evaluate (GD)	0.857	0.691	0.774	0.724
Evaluate (GD) + polyres	0.829	0.649	0.739	0.685
Calibrate	0.930	**0.716**	**0.823**	**0.759**
Calibrate + polyres	**0.983**	0.662	**0.823**	0.726
MACHINA	0.968	0.643	0.806	0.708

Table 3. Average F1-scores of migration graph for each broad seeding pattern (primary-only seeding or metastasis-to-metastasis seeding) on simulated data. All Metient models were run with a sample size of 1024. When multiple solutions are found for a given input, all lowest loss solutions were taken. Evaluate (MP): Metient in evaluate mode with maximum parsimony only. Evaluate (GD): Metient in evaluate mode with genetic distance only. Calibrate: Metient in calibrate mode, using genetic distance as the metastasis prior. polyres: polytomy resolution is used. mS: monoclonal single-source seeding. pS: polyclonal single-source seeding. pM: polyclonal multi-source seeding. pR: polyclonal reseeding.

Average migrating clone F1-scores

Method	Primary-only	Met-to-met	Macro-F1	Micro-F1
Evaluate (MP)	0.795	0.781	0.788	0.784
Evaluate (MP) + polyres	0.873	0.791	0.832	0.808
Evaluate (GD)	0.954	0.876	0.915	0.892
Evaluate (GD) + polyres	**0.979**	**0.928**	**0.954**	**0.939**
Calibrate	0.961	0.916	0.938	0.925
Calibrate + polyres	0.961	0.890	0.926	0.905
MACHINA	0.954	0.876	0.915	0.892

Table 4. Average F1-scores of migrating clones for each broad seeding pattern (primary-only seeding or metastasis-to-metastasis seeding) on simulated data. All Metient models were run with a sample size of 1024. When multiple solutions are found for a given input, all lowest loss solutions were taken. Evaluate (MP): Metient in evaluate mode with maximum parsimony only. Evaluate (GD): Metient in evaluate mode with genetic distance only. Calibrate: Metient in calibrate mode, using genetic distance as the metastasis prior. polyres: polytomy resolution is used.

bioRxiv preprint doi: https://doi.org/10.1101/2024.07.09.602790; this version posted September 3, 2024. The copyright holder for this preprint (which was not certified by peer review) is the author/funder, who has granted bioRxiv a license to display the preprint in perpetuity. It is made available under aCC-BY 4.0 International license.

Supplementary Information

A. Evaluating migration histories

We present our technique for optimizing migration histories in the context of variational inference. Our goal is to approximate the conditional density of latent variable $\mathbf{V}$ given observed variables $\mathbf{U}$ and $\mathbf{T}$: $p(\mathbf{V} \mid \mathbf{U}, \mathbf{T})$. $\mathbf{U}$ has been optimized as described in the section "Estimating observed clone proportions" in Methods. $p(\mathbf{V} \mid \mathbf{U}, \mathbf{T})$ can be written as:

$$p(\mathbf{V} \mid \mathbf{U}, \mathbf{T}) = \frac{p(\mathbf{U}, \mathbf{T} \mid \mathbf{V}) p(\mathbf{V})}{p(\mathbf{U}, \mathbf{T})} \tag{S1}$$

We cannot calculate the denominator, or the evidence, as its derivation is intractable (there are many possible values of $\mathbf{V}$):

$$p(\mathbf{U}, \mathbf{T}) = \sum_{\mathbf{V}} p(\mathbf{U}, \mathbf{T}, \mathbf{V}) \tag{S2}$$

We approximate the posterior distribution $p(\mathbf{V} \mid \mathbf{U}, \mathbf{T})$ with a simpler distribution $q(\mathbf{V})$, and we aim to minimize the Kullback-Leibler (KL) divergence between $q(\mathbf{V})$ and the true posterior $p(\mathbf{V} \mid \mathbf{U}, \mathbf{T})$. The Evidence Lower Bound (ELBO) is given by:

$$\text{ELBO}(q) = \mathbb{E}_{q(\mathbf{V})}[\log p(\mathbf{U}, \mathbf{T}, \mathbf{V})] + \mathbb{H}(\mathbf{V}) \tag{S3}$$

Where the second term is the entropy term.

To handle the categorical nature of $\mathbf{V}$, we use the Gumbel-Softmax reparameterization trick to optimize $\mathbf{V}$. Starting with a matrix $\psi \in \{0,1\}^{K \times C}$, of randomly initialized values, where K is the number of anatomical sites and C is the number of clones, and each column represents the unnormalized log probabilities of clone c being labeled in site k:

1. At every iteration, for each clone c, we sample $g_{1c}...g_{kc}$, k i.i.d. samples from Gumbel(0,1) and compute $y_{ic} = \psi_{ic} + g_{ic}$. Where a sample g from the Gumbel is computed as:

$$g = -\log(-\log(u)) \quad \text{where} \quad u \sim \text{Uniform}(0,1) \tag{S4}$$

2. We then sample from the categorical distribution represented by the column vector $\psi_{:c}$ by setting $i^* = \text{argmax}_i\, y_{ic}$ and represent that sample with a one-hot encoding in $\mathbf{V}$, i.e., $\mathbf{V}_{ic} = 1$ if $i = i^*$, 0 otherwise.

3. Then we evaluate the ELBO(ν) where

$$\nu_{ic} = \frac{\exp(y_{ic}/\tau)}{\sum_{j=1}^{k} \exp(y_{jc}/\tau)} \qquad \text{for } i = 1, ..., k,$$

   using a stochastic approximation based on $\mathbf{V}$, and take the gradient of this ELBO in the backward pass, thus implementing the straight-through estimator.

bioRxiv preprint doi: https://doi.org/10.1101/2024.07.09.602790; this version posted September 3, 2024. The copyright holder for this preprint (which was not certified by peer review) is the author/funder, who has granted bioRxiv a license to display the preprint in perpetuity. It is made available under aCC-BY 4.0 International license.

4. During training, start with a high τ to permit exploration, then gradually anneal τ to a small but non-zero value so that the Gumbel-Softmax distribution, ν resembles a one-hot vector.

At the end of training, as τ approaches 0, then the gradient becomes unbiased and ν approaches $\mathbf{V}$. In order to capture multiple modes of the posterior distribution, each representing different hypotheses about the migration history, we optimize multiple $\mathbf{V}$s in parallel. To do this, we set up steps 1-3 such that x ψs are solved for in parallel[58] (with a different random initialization for each parallel process), where x is equal to the sample size and is calculated according to the size of the inputs ($\propto K^C$).

Using the Gumbel-Softmax reparameterization as described above, we approximate the expectation in the ELBO with a sample of $\mathbf{V}$, which we denote $\tilde{\mathbf{V}}$:

$$\mathbb{E}_{q(\mathbf{V})}[\log p(\mathbf{U}, \mathbf{T}, \mathbf{V})] \approx \log p(\tilde{\mathbf{V}}, \mathbf{U}, \mathbf{T}) \quad \textbf{(S5)}$$

$$\mathbb{H}(\mathbf{V}) \approx -\sum_{j=1}^{C}\sum_{k=1}^{K} q(\tilde{\mathbf{V}}_{jk}) \log q(\tilde{\mathbf{V}}_{jk}) \quad \textbf{(S6)}$$

In the following sections, we describe how we calculate $p(\tilde{\mathbf{V}}, \mathbf{U}, \mathbf{T})$, which is broken down into (1) $p_m(\tilde{\mathbf{V}}, \mathbf{U}, \mathbf{T})$, i.e., the scoring of $\tilde{\mathbf{V}}$ using maximum parsimony, (2) $p_g(\tilde{\mathbf{V}}, \mathbf{U}, \mathbf{T})$, i.e., the scoring of $\tilde{\mathbf{V}}$ using genetic distance, and (3) $p_o(\tilde{\mathbf{V}}, \mathbf{U}, \mathbf{T})$, i.e., the scoring of $\tilde{\mathbf{V}}$ using organotropism.

A.1. Evaluating maximum parsimony. As previously described by MACHINA[17], the maximum parsimony metrics are defined as:

- **migration number** m: Given clone tree $\mathbf{T}$ and clone tree labeling $\mathbf{V}$, the migration number is the number of edges in $\mathbf{T}$ where the outgoing node and incoming node have a different label. It is the number of edges in migration graph $\mathbf{G}$.

- **comigration number** c: Given clone tree $\mathbf{T}$ and clone tree labeling $\mathbf{V}$, the comigration number is a subset of the migration edges between two anatomical sites, such that the migration edges occur on distinct branches of the clone tree. It is the number of multi-edges in migration graph $\mathbf{G}$ if $\mathbf{G}$ does not contain cycles.

- **seeding site number** s: Given a clone tree $\mathbf{T}$ and clone tree labeling $\mathbf{V}$, the seeding site number is the number of unique anatomical sites with an outgoing edge. It is the number of edges in migration graph $\mathbf{G}$ with an outgoing edge.

bioRxiv preprint doi: https://doi.org/10.1101/2024.07.09.602790; this version posted September 3, 2024. The copyright holder for this preprint (which was not certified by peer review) is the author/funder, who has granted bioRxiv a license to display the preprint in perpetuity. It is made available under aCC-BY 4.0 International license.

Maximum parsimony scoring calculates the number of migrations m , comigrations c, and seeding sites s.

$$p_m(\tilde{\mathbf{V}}, \mathbf{U}, \mathbf{T}) = w_m \cdot m + w_c \cdot c + w_s \cdot s \tag{S7}$$
$$m = \sum_{ij} \mathbf{G} - Trace(\mathbf{G})$$
$$s = \sum_{j=1}^{n} \left(\left(\sum_{i=1}^{m} (\mathbf{G} \odot (\mathbf{J}_K - \mathbf{I}_K))_i \right)^* \right)_j$$
$$c = \sum_{ij} \mathbf{G}^*_{ij} - Tr(\mathbf{G}^*) + \sum_{ij} \left(\sum_{l=1}^{m} (\mathbf{P} \odot (\mathbf{W} \odot \mathbf{X}))_l \right)_{ij}$$

where $\mathbf{G} = \tilde{\mathbf{V}}\mathbf{T}\tilde{\mathbf{V}}^T, \mathbf{P} = \left(\mathbf{T} \vee \mathbf{I}_N\right)^{N-1}, \mathbf{X} = \tilde{\mathbf{V}}^T\tilde{\mathbf{V}}, \mathbf{Y} = \sum_{i=1}^{m} \left((\tilde{\mathbf{V}}\mathbf{T}\tilde{\mathbf{V}}^T \odot (\mathbf{J}_{CK} - \mathbf{V}^T)\right), \mathbf{Z}^* = \mathbf{sign}(\mathbf{Z})$. $\vee$ represents boolean matrix multiplication, $\mathbf{I_n}$ is a $n \times n$ identity matrix, $\odot$ is the Hadamard, i.e., element-wise product, and $\mathbf{J}_{mn}$ is a matrix of ones with dimensions $m \times n$.

A.2. Evaluating genetic distance. Genetic distance is a measure of the number of mutations between clones. Given a distance matrix $\mathbf{D}$ which has normalized genetic distances between every clone:

$$p_g(\tilde{\mathbf{V}}, \mathbf{U}, \mathbf{T}) = \frac{w_g}{m} \sum_{ij} -log(\mathbf{D}) \odot \mathbf{T} \odot (\mathbf{J}_C - \mathbf{X}) \tag{S8}$$

where $\mathbf{J}_C$ is a square matrix of ones, $\odot$ is the Hadamard, i.e., element-wise product, and $\mathbf{X} = \tilde{\mathbf{V}}^T\tilde{\mathbf{V}}$. The product $\mathbf{T} \odot \mathbf{J}_C - \mathbf{X}$ tells us if two nodes have an edge between them and they are in different sites. Taking the hadamard product of this with the negative log of $\mathbf{D}$ gives lower scores to edges with higher genetic distances. We normalize by the migration number m so we don't implicitly penalize migration histories with more migrations through this scoring.

A.3. Evaluating organotropism. Organotropism refers to the observation that certain cancers metastasize to specific organs. We penalize migration edges between organs that are less likely to occur based on clinical data. Given a vector $\mathbf{o}$ which contains the frequency that a primary tumor seeds other anatomical sites:

$$p_o(\tilde{\mathbf{V}}, \mathbf{U}, \mathbf{T}) = \frac{w_o}{m_p} \sum_{i=1}^{K} -log(\mathbf{o}) \odot (\mathbf{G} \odot (\mathbf{J}_K - \mathbf{I}_K))_{p,i} \tag{S9}$$

where $\mathbf{G} = \tilde{\mathbf{V}}\mathbf{T}\tilde{\mathbf{V}}^T$, $\odot$ is the Hadamard, i.e., element-wise product, $\mathbf{J}_K$ is a square matrix of ones, and $\mathbf{I_K}$ is the identity matrix. The product $(\mathbf{G} \odot (\mathbf{J}_K - \mathbf{I}_K))$ contains the number of migrations between different sites, and taking the Hadamard product of this with the negative log of $\mathbf{o}$ gives lower scores to migration edges with higher organotropism frequencies. The subscript p, i represents taking the row of $(\mathbf{G} \odot (\mathbf{J}_K - \mathbf{I}_K))$ which represents the primary site index and summing over the columns at every other anatomical site i. We normalize by m_p, the number of migrations originating from the primary site, so we don't implicitly penalize migration histories with more migrations through this scoring.

bioRxiv preprint doi: https://doi.org/10.1101/2024.07.09.602790; this version posted September 3, 2024. The copyright holder for this preprint (which was not certified by peer review) is the author/funder, who has granted bioRxiv a license to display the preprint in perpetuity. It is made available under aCC-BY 4.0 International license.

B. Calibrate alignment

A parsimony model is represented by a set of parsimony weights – w_m, w_c, and w_s – assigned, respectively, to the number of migrations (indicated by m), comigrations (c), seeding sites (s). A migration history's parsimony score, p, is the model-weighted average of these three parsimony metrics, i.e., $p = w_m m + w_c c + w_s s$ (Equation S7). Different parsimony models favor different histories on the Pareto front. To fit a parsimony model to a cancer type-specific cohort, we look at how well the maximum parsimony distribution aligns with the genetic distance distribution of each patient's migration history trees.

Take a cohort of N patients, where each patient, n, is associated with a set,

$$S^{(n)} = \left\{ t_i^{(n)} \right\}_{i=1}^{T^{(n)}},$$

of $T^{(n)}$ migration histories. Each migration history t is associated with a genetic distance g_t (or, alternatively, an organotropism score), and a vector of parsimony metrics $\mathbf{x}_t = [m_t \; c_t \; s_t]$ (i.e., the counts of migrations, comigrations, and seeding sites, respectively). The goal is to set the parameters, $\theta = [w_m \; w_c \; w_s]$ of the parsimony prior $q(t) \propto \exp\left(-\mathbf{x}_t^T \theta\right)$ so that it matches, as best as possible, a target distribution, $p(t)$, over the migration histories t implied by the g_t, where $p(t) \propto \exp(-\tau g_t)$ and τ is a user-defined "temperature" hyper-parameter.

To fit these parameters, we define patient-specific categorical distributions $p^{(n)}(t)$ and $q^{(n)}(t)$ as follows. Let $\mathbf{g}^{(n)}$ be the vector of length $T^{(n)}$ of genetic distances of the migration histories for patient n, where $g_i^{(n)}$ is the genetic distance for the i-th tree. And let the column vector $\mathbf{x}_i^{(n)}$ be the parsimony metrics for the i-th migration history associated with patient n. We will append the $T^{(n)}$ vectors $\mathbf{x}_i^{(n)}$ to make a $3 \times T^{(n)}$ design matrix $X^{(n)}$. Also we define the vector-valued softmax function in the typical way, i.e.,

$$\text{softmax}(\mathbf{v})_i = \frac{\exp(v_i)}{\sum_{j=1}^{|\mathbf{v}|} \exp(v_j)}$$

where softmax$(\mathbf{v})_i$ is the i-th element of the vector output by softmax$(\mathbf{v})$. Then the "parsimony" probability distribution over the trees for patient n is represented by the vector $\mathbf{q}^{(n)}$

$$\mathbf{q}^{(n)} = \text{softmax}(-\theta^T X^{(n)})$$

and the target distribution by the vector $\mathbf{p}^{(n)}$

$$\mathbf{p}^{(n)} = \text{softmax}(-\tau \mathbf{g}^{(n)}).$$

bioRxiv preprint doi: https://doi.org/10.1101/2024.07.09.602790; this version posted September 3, 2024. The copyright holder for this preprint (which was not certified by peer review) is the author/funder, who has granted bioRxiv a license to display the preprint in perpetuity. It is made available under aCC-BY 4.0 International license.

Then we define the cohort calibration objective $E(\theta)$ as an average cross-entropy over the patient cohort, i.e.,

$$E(\theta) = \sum_{n=1}^{N} w_n \left(\sum_{i=1}^{T^{(n)}} p_i^{(n)} \log q_i^{(n)} \right)$$

and the MLE estimate of the parameters is $\theta^* = \text{argmax}_\theta E(\theta)$. w_n is set to $log(E/(r \cdot b))$, where E is the number of internal edges of a patient's clone tree, r is the number of possible primaries for the patient, and b is the number of possible clone trees for a given patient (so as not to bias towards patients with multiple possible primaries or multiple possible clone trees). Since the number of edges is equal to the maximum number of migrations possible in a tree, it is also equal to the number of possible genetic distance observations that that tree can provide in the genetic distance scoring of that tree. Therefore, w_n is representative of the information content that a patient can provide when fitting θ.

B.1. Specifying the target distribution by setting the temperature parameter. The use of $E(\theta)$ to set θ requires that for a patient n that, generally speaking, the genetic distance $g_i^{(n)}$ for a potential migration history, represented by a tree i, is lower for more probable histories. However, because $E(\theta)$ is minimized when $\tau \mathbf{g}^{(n)} = \theta X^{(n)} + c\mathbf{1}$ for some constant c, this could be a very strong assumption, one that we might not always be comfortable making.

Fortunately, we can set τ to increase the correctness of this assumption. Notice that in the limit of large τ that

$$\lim_{\tau \to \infty} E(\theta) = \sum_{n=1}^{N} w_n \ \log q_{i_n^*}^{(n)}$$

where $i_n^* = \text{argmin}_i \ g_i^{(n)}$, assuming that the minimum is unique. If the minimum is not unique then the above is true if we replace $\log q_{i_n^*}^{(n)}$ with the average of $\log q_t^{(n)}$ of all the trees t that have the minimum genetic distance for patient n.

So, in other words, if we set τ to be very large, then $E(\theta)$ is just the (weighted) sum of the log probabilities of the minimum genetic distance trees in each patient, and optimizing $E(\theta)$ corresponds to maximizing the parsimony probabilities of the best scoring trees per patient under the genetic distance score.

$$\prod_i \frac{exp(X^{(i)^\tau} \theta)}{\sum_{j|rank(j) \geq rank(i)} exp(X^{(j)^\tau} \theta)}$$

So, we set τ to be large, such that τ is multiple times the maximum genetic distance (assuming that the genetic distance is always positive). We do the same for the organotropism prior.

C. Case-by-case differences to expert annotations

C.1. Comparisons to Melanoma patients from Sanborn et al. Migration histories generated for the metastatic melanoma cohort using Metient-calibrate agree with the expert analysis that most melanoma patients exhibit primary single-source seeding (7/7 patients; Supplementary Figure S4). For patient F (Supplementary Figure S4c), our

bioRxiv preprint doi: https://doi.org/10.1101/2024.07.09.602790; this version posted September 3, 2024. The copyright holder for this preprint (which was not certified by peer review) is the author/funder, who has granted bioRxiv a license to display the preprint in perpetuity. It is made available under aCC-BY 4.0 International license.

reconstruction of the clone tree and observed clones does not suggest that a lymph node to distant metastasis seeding event is likely, but that this patient also likely exhibits a primary-only seeding pattern. In the second best solution predicted for patient D, Metient predicts that a locoregional skin metastasis from the right ankle could have given rise to subsequent metastases, supporting one of the possible paths (in dotted lines) that the original authors propose (Supplementary Figure S4d). We also predict a primary single-source solution on the Pareto front which is another possible path proposed by the authors (Supplementary Figure S4d).

C.2. Comparisons to HGSOC patients from McPherson et al.. In the seven HGSOC patients, predicted migration histories by McPherson et al.[4] were made available using an algorithm that only minimizes migrations (Sankoff algorithm[60]). We find that four out of seven patients are in complete agreement (Supplemental Figure S5). For patient 1, by resolving polytomies, we offer an explanation with less migrations and comigrations, and predict that the left fallopian tube rather than the small bowel served as a possible intermediate site before further metastatic dissemination (Supplemental Figure S5a). For patient 3, we offer an explanation with less migrations, comigrations and seeding sites, suggesting that all metastases were seeded from the primary (Supplemental Figure S5c). Finally for patient 7, solving for polytomies allows us to reduce the migration number by 1 from the right uterosacral to left ovary, although the overall seeding pattern is in agreement (Supplemental Figure S5d).

C.3. Comparisons to HR-NB patients from Gundem et al.. Because the HR-NB annotations only indicate the presence of a migration between two sites and not the directionality, we compared our site-to-site migrations (i.e., a binarized representation of migration graph $\mathbf{G}$ (Figure 1c)) to those that were previously reported. We looked at the 14 HR-NB patients for which there were manual expert annotations from Gundem et al.[9], and found that we predict the same overall site-to-site migrations for 10 out of 14 cases. For patient H103207, we predict their before therapy pattern on the Pareto front (Solution 3 in Figure S2a), but we prioritize two solutions with metastasis-to-metastasis seeding between the lung and the liver. A subset of this seeding between the liver and two lobes of the lung is suggested in their after therapy hypothesis of spread (Figure S2a). While Gundem et al. suggest seeding between the two lobes of the lung as well as from each lobe of the lung to the liver, we infer a simpler, serial progression, where the right lung lower lobe seeds the liver, which subsequently seed the left lung lower lobe (Solution 1 in Figure S2a). For patient H132396, Metient prioritizes migration histories with fewer migrations (Solutions 1 and 2 in Figure S2g), but presents the expert annotation on the Pareto front (Solution 3 in Figure S2g). For patient H132384, Metient proposes bone-to-bone secondary metastasis formation (Solution 1 in Figure S3d), but again presents the expert annotation on the Pareto front (Solution 2 in Figure S3d). For patient H134821, we infer the same pancreas to hilar lymph node seeding proposed by the authors as spread after therapy, but suggest that all other metastases were seeded directly by the primary (Solution 1 in Figure S3f). However, we report the same metastasis-to-metastasis seeding between the cervical and thoracic lymph nodes and cervical metastases as the authors in alternative solutions on the Pareto front (Solutions 3-5 in Figure S3f).

bioRxiv preprint doi: https://doi.org/10.1101/2024.07.09.602790; this version posted September 3, 2024. The copyright holder for this preprint (which was not certified by peer review) is the author/funder, who has granted bioRxiv a license to display the preprint in perpetuity. It is made available under aCC-BY 4.0 International license.

D. Model choice impacts downstream analyses

As we were analyzing different aspects of metastatic dissemination, we asked how these answers might change if a seeding model is enforced when reconstructing a patient's migration history. To highlight how the choice of seeding model can impact the analysis and interpretation of metastatic dissemination, we compared the migration histories produced by three models: (1) assumption of primary, single-source seeding, (2) the MACHINA assumptions, which first minimize migrations, and then break ties based on comigration number followed by seeding site number, and finally (3) the adaptive Metient model fit to each cohort. As expected, a primary, single-source seeding model chooses a primary, single-source dissemination pattern for 100% of patients (Supplementary Figure S1c). The migration penalizing model chooses a primary single-source seeding explanation in 82.6% of patients, and Metient falls in between the two, choosing a primary single-source seeding explanation in 86.2% of patients (Supplementary Figure S1d). Importantly, since Metient can recover and evaluate the relative trade-offs of the parsimony metrics, when choosing a primary single-source solution, our model has either not found a plausible metastasis-to-metastasis explanation for a patient's data on the Pareto front, or has used the metastasis priors to deem such an explanation less likely. In contrast, previous models do not automatically recover multiple possible hypotheses, therefore reducing confidence in these algorithms' choice of best history.

In addition to having an impact on the inferred seeding patterns, a model that assumes primary single-source seeding also changes other interpretations of metastatic seeding. We asked two questions about the best migration histories produced by the two extremes of models, i.e. the assumption of primary, single-source seeding and Metient: (1) the frequency in which a new seeding site is added, and (2) the frequency of polyclonal migrations between two sites. As expected, a model which assumes primary, single-source seeding promotes migration histories with only one seeding site (Supplementary Figure S1e). In turn, such a model infers a higher fraction of polyclonal migrations (Supplementary Figure S1a) compared to the histories prioritized by Metient. The trade-off between polyclonality and seeding sites occurs because additional seeding sites reduce the number of migration edges that must be placed between the primary and all other metastases. Balancing this trade-off correctly is important as it impacts the interpretation of seeding clonality as well as which clones perform seeding. Specifically, 9% (15/167) of patients have differing colonizing clones between the two models, changing the inference of which clones, and therefore which mutations, have metastatic competence.

E. Bulk DNA sequencing pre-processing

E.1. Variant read probability calculation (ω). In order to account for non-diploid copy number and tumor purities, we require a variant read probability ω to be input for every genomic locus in each sample. For a given sample s and variant allele j, the variant read probability ω_{js} is the probability of observing a read with the variant allele at that locus in a cell with the mutation, and is calculated as:

bioRxiv preprint doi: https://doi.org/10.1101/2024.07.09.602790; this version posted September 3, 2024. The copyright holder for this preprint (which was not certified by peer review) is the author/funder, who has granted bioRxiv a license to display the preprint in perpetuity. It is made available under aCC-BY 4.0 International license.

$$\omega_{js} = M_{js}/N_{js} \tag{S10}$$

where M_{js} is the number of copies of the mutant allele j in sample s in the cells that contain the mutant allele, and N_{js} is the average number of copies at the genomic locus of the mutation j in all cells in s.

To account for the fact that cancer cells frequently have different numbers of copies at genomic loci compared to normal cells, N_{js} is calculated as:

$$N_{js} = \rho_s N_{js}^{(c)} + (1 - \rho_s) N_{js}^{(h)} \tag{S11}$$

where:

- $N_{js}^{(c)}$ is the population average copy number of the locus which contains mutant allele j in the cancer cell population
- $N_{js}^{(h)}$ is the copy number at the genomic locus of mutation j in the normal cell population. In diploid cells this is 2, and in haploid cells this is 1.
- ρ_s is the tumor purity of sample s

ρ_s and $N_{js}^{(c)}$ (and sometimes N_{js}) are normal outputs from a copy number calling pipeline. We suggest setting $M_{js} = 1$ unless there is strong evidence that the j allele has been amplified. In this case, allele-specific copy number callers provide the major allele copy number A_{js} and minor allele copy number B_{js}, where $N_{js}^{(c)} = A_{js} + B_{js}$, and $M_{js} = A_{js}$. When a locus is impacted by many different CNAs, accurately estimating M_{js} is challenging since there are likely subclonal changes in the multiplicity of the j allele, in which case we recommend excluding these mutations. For additional information on how to estimate M_{js} and N_{js} please refer to Tarabichi et al.[61].

If clustering is used, we have to properly combine multiple SNV loci with different potential variant read probabilites. To do this, we rescale the reference and variant allele read counts for each locus and then set its variant read probability to 0.5 before combining variants within a cluster (where we add the reference and variant allele read counts for all variants within a cluster). This rescaling allows us to effectively treat the variant as coming from a diploid locus. To achieve this, we use the following rescaling formulas, which has been previously described in Wintersinger et al.[53]:

bioRxiv preprint doi: https://doi.org/10.1101/2024.07.09.602790; this version posted September 3, 2024. The copyright holder for this preprint (which was not certified by peer review) is the author/funder, who has granted bioRxiv a license to display the preprint in perpetuity. It is made available under aCC-BY 4.0 International license.

$$T_{js} = V_{js} + R_{js}$$

$$\hat{T}_{js} = 2\omega_{js} T_{js}$$

$$\hat{V}_{js} = \min(V_{js}, \hat{T}_{js})$$

$$\hat{R}_{js} = \hat{T}_{js} - \hat{V}_{js}$$

$$\hat{\omega}_{js} = \frac{1}{2}$$

Where T_{js} is the input count of total reads, V_{js} is the input count of variant reads, R_{js} is the input count of reference reads, and ω_{js} is the variant read probability at a genomic locus j in anatomical site s. The rescaled total, reference, and variant allele read counts and variant read probability are $\hat{T}_{js}, \hat{V}_{js}, \hat{R}_{js}$ and $\hat{\omega}_{js}$, respectively.

E.2. Breast Cancer Dataset. The single nucleotide variant calls from two breast cancer patients with whole genome sequencing data were taken from Hoadley et al.[20]. The variant calls were in copy number neutral variant positions and tumor purity was not reported, so reference and variant counts along with defaults for tumor purity, major copy number and minor copy number (defaults are 1.0, 1, 1, respectively) were inputted into PyClone-0.13.1 clonal analysis[62]. PyClone's MCMC chain was run for 100,000 iterations, discarding the first 50,000 as burnin. Orchard was run using the PyClone clusters as input with -p flag to force trees to be monoprimary (come from a singular root cancer clone) and all variant read probabilities set to the default of 0.5, since SNVs from regions with CNAs were excluded, and tumor purity was not reported and thus assumed to be 1. We ran Metient-evaluate on this data using all default configurations (dynamically calculated sample size based on size of input clone tree and number of anatomical sites).

E.3. High-grade Serous Ovarian Cancer Dataset. To better compare to McPherson et al.'s own migration history analysis, we used the mutation clusters, clone trees and cellular prevalences of each clone that they estimate and report[4]. Metient was run with the $\mathbf{U}$ matrix inputted, and we solve for $\mathbf{V}$ for each patient. We ran Metient-calibrate on this data using all default configurations (dynamically calculated sample size based on size of input clone tree and number of anatomical sites) and with polytomy resolution.

E.4. Melanoma Dataset. The single nucleotide variant and copy number calls from eight melanoma patients with whole exome sequencing data were taken from Sanborn et al.[3], along with estimated tumor purity. Only SNVs in copy number neutral regions were considered. Patient H was excluded due to a lack of copy number neutral SNVs. Reference and variant read counts along with major and minor copy number and tumor purity were inputted into PyClone-VI 0.1.3 for clonal analysis[63]. PyClone-VI's fit command was run with all default parameters. Orchard was run using the PyClone clusters as input with -p flag to force trees to be monoprimary (come from a singular root cancer clone). Variant read probabilities for Orchard were calculated using major copy number, minor copy number and tumor purity according to Equation S10. We ran Metient-calibrate with the clonal proportions estimated

bioRxiv preprint doi: https://doi.org/10.1101/2024.07.09.602790; this version posted September 3, 2024. The copyright holder for this preprint (which was not certified by peer review) is the author/funder, who has granted bioRxiv a license to display the preprint in perpetuity. It is made available under aCC-BY 4.0 International license.

by running Orchard (i.e., η in Orchard's output) using all default configurations and with polytomy resolution.

E.5. Neuroblastoma Dataset. Access to multi-WGS data for 45 neuroblastoma patients was provided through dbGaP accession phs03111[9]. Of these 45 patients, 27 patients had at least one primary and one metastatic tumor sample with a tumor purity of >10%, and all analysis was conducted on this patient subset. Single nucleotide variant, copy number calls and tumor purities were collected from this dataset, and clusters produced from the original paper using DPClust[64] were used. Multiple samples for the same anatomical site and sample time (i.e., diagnosis, therapy-naive re-resection, therapy resection during induction chemotherapy, relapse or further relapse) were combined by pooling reference and variant allele counts. Orchard was run using the DPClust clusters as input with -p flag to force trees to be monoprimary (come from a singular root cancer clone). Variant read probabilities for Orchard and Metient were calculated using major copy number, minor copy number and tumor purity according to Equation S10. We ran Metient-calibrate with the clonal proportions estimated by running Orchard (i.e., η in Orchard's output) using all default configurations and with polytomy resolution.

For three patients (H103207, H132388, H134822), multiple primary tumor samples were collected at different time points (diagnosis and resection during therapy). For these patients, we treated the therapy resection and diagnosis tumor as multiple samples from the same anatomical site if the anatomical site was labeled the same, and as two different primaries if the anatomical sites were different. The therapy resections were usually taken a few months after diagnosis tumor samples.

E.6. Non-small Cell Lung Cancer Dataset. We used the clustered SNVs, clone trees and observed clone proportions made available by the TRACERx consortium for 126 non-small cell lung cancer (NSCLC) patients (downloaded from https://zenodo.org/record/7649257). When samples for multiple regions of a tumor were available, the reference and variant allele counts were summed together to generate reference and variant allele counts for the entire tumor. Since we model variant allele counts as binomially distributed with n total reads (variant + reference) and p probability of generating a variant read, this summing assumes that each sampled region of a tumor has the same probability p. Metient was run with the $\mathbf{U}$ matrix inputted, and we solve for $\mathbf{V}$ for each patient. We ran Metient-calibrate on this data using all default configurations (dynamically calculated sample size based on size of input clone tree and number of anatomical sites) and with polytomy resolution.
